# Supplementary material for: FADS1/2 control lipid metabolism and ferroptosis susceptibility in triple-negative breast cancer
Source: EMBO Mol Med. 2024 Jun 26;16(7):5. doi: 10.1038/s44321-024-00090-6 (PMC11251055; doi:10.1038/s44321-024-00090-6)
Supplement: Supplementary file 1 — Appendix [file 44321_2024_90_MOESM1_ESM.pdf]

Appendix Figures S1-S3

FADS1/2 control lipid metabolism and ferroptosis susceptibility in triple-negative breast cancer

Lorito, Subbiani et al., 2024

The Appendix Figures file contains:

Appendix Figure S1	page 2
Appendix Figure S2	page 3
Appendix Figure S3	page 5

Correspondence should be addressed to andrea.morandi@unifi.it

Appendix Figure S1

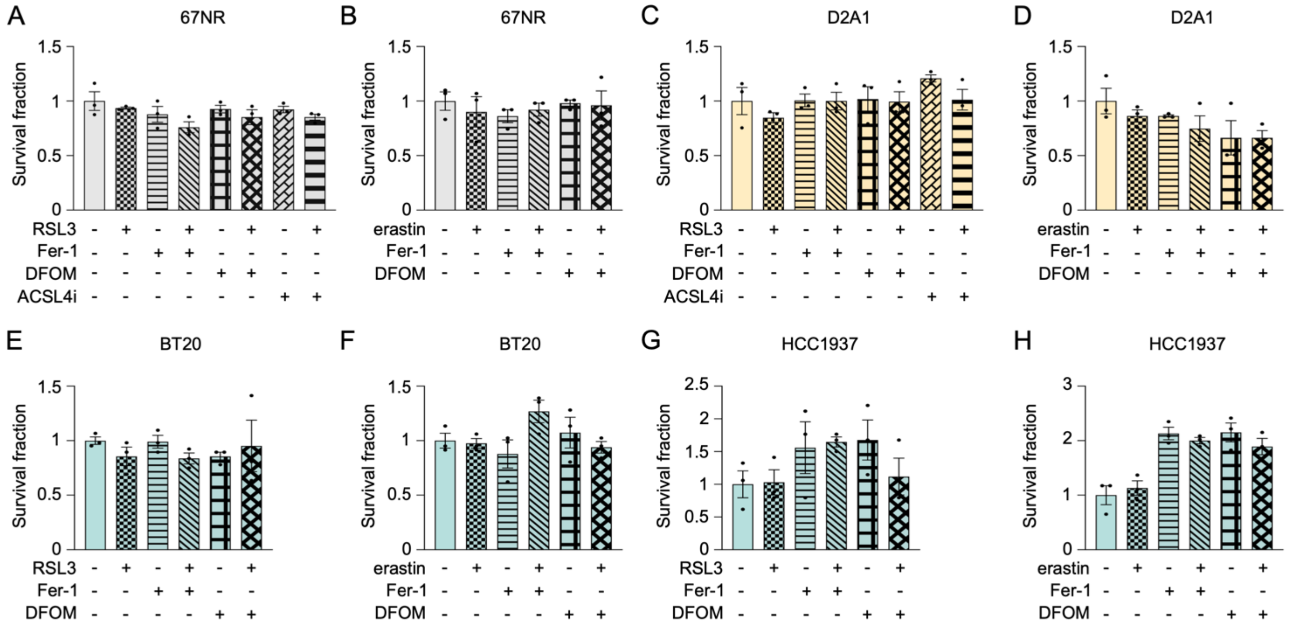

Appendix Figure S1. Weakly metastatic TNBC cell lines show resistance to ferroptosis-inducing agents.

(**A-D**) Murine 67NR (A,B) and D2A1 (C,D) poorly metastasizing cells were pre-treated with 15 μM Fer-1, 5 μM DFOM, or 10 μM ACSL4i for 4 hours and then administrated ON with 0.1 μM (67NR) or 0.25 μM (D2A1) RSL3 (A,C) and with 0.5 μM of erastin (B,D). After 24 hours cells were subjected to cell viability assay; (*n* = 3 biological replicates). The RSL3- or erastin-treated condition was used as comparator in the statistical analysis. (**E-H**) Human BT20 (E,F) and HCC1937 (G,H) poorly metastasizing cells were pre-treated with 15 μM Fer-1, 5 μM DFOM, or 10 μM ACSL4i for 4 hours and then administrated ON with 0.25 μM RSL3 (E,G) and with 0.5 μM erastin (F,H). After 24 hours cells were subjected to cell viability assay; (*n* = 3 biological replicates). The RSL3- or erastin-treated condition was used as comparator in the statistical analysis. Data information: data are presented as mean ± SEM. Statistical analysis was performed using One-way ANOVA followed by Dunnett's correction.

Appendix Figure S2

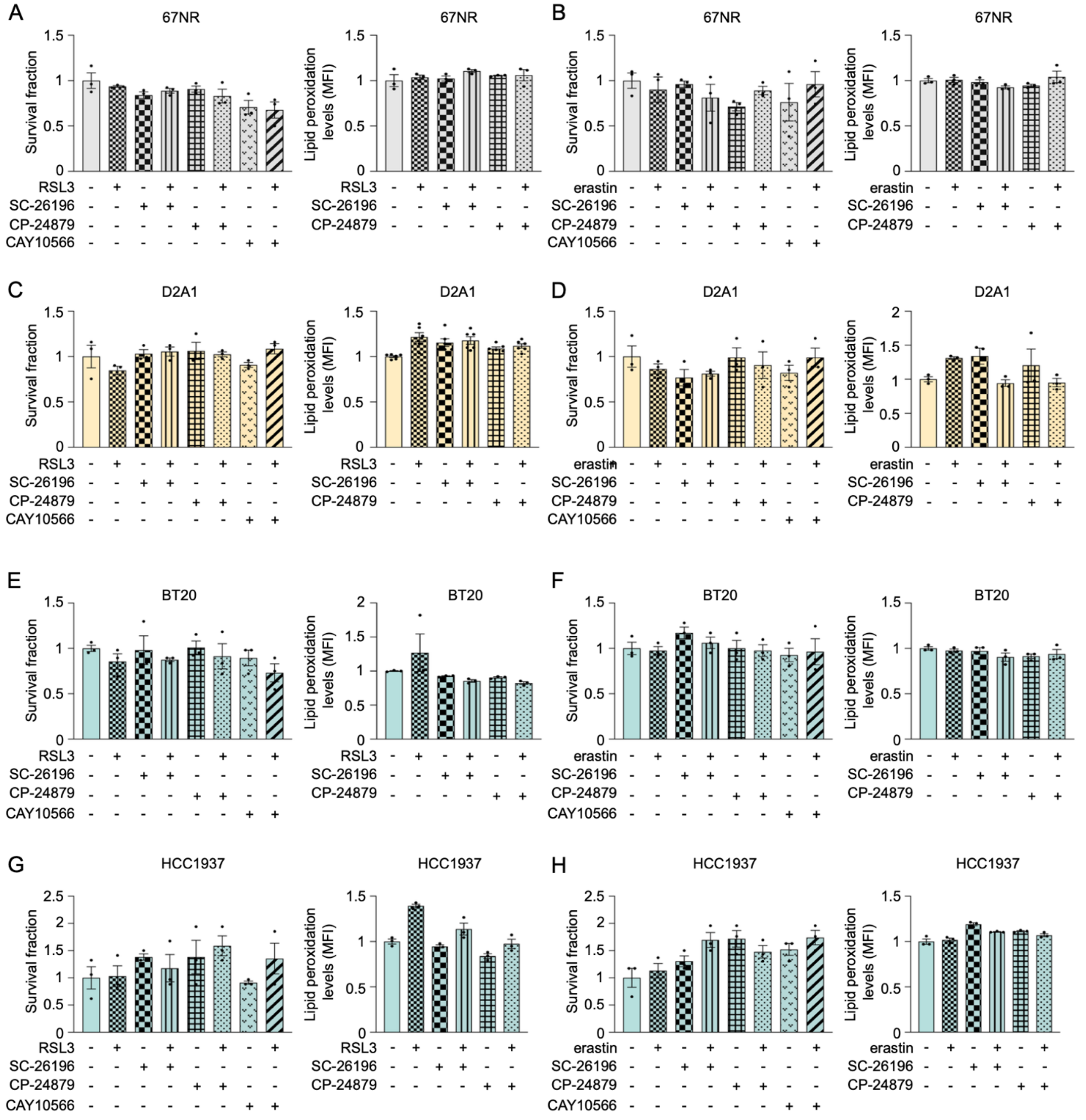

Appendix Figure S2. Weakly metastatic TNBC cell lines are insensitive to FADS1/2 targeting.

(**A,B**) Murine 67NR poorly metastasizing cells were pre-treated with 10 μM FADS2i (SC-26196), FADS1/2i (CP-24879), and SCD1i (CAY10566) for 4 hours, then administrated (i) ON with 0.1 μM RSL3 (A, left) or 0.5 μM erastin (B, left) before being subjected to cell viability assay, and (ii) for 2 hours with 1 μM RSL3 (A, right) or 5 μM erastin (B, right) before being subjected to cytofluorimetric analysis to measure lipid peroxidation; (*n* = 3 biological replicates). The RSL3- or erastin-treated condition was used as comparator in the statistical analysis. (**C,D**) Murine D2A1 poorly metastasizing cells were pre-treated with 10 μM FADS2i (SC-26196), FADS1/2i (CP-24879), and SCD1i (CAY10566) for 4 hours, then

administrated (i) ON with 0.25 μM RSL3 (C, left) or 0.5 μM erastin (D, left) before being subjected to cell viability assay, and (ii) for 2 hours with 1 μM RSL3 (C, right) or 5 μM erastin (D, right) before being subjected to cytofluorimetric analysis to measure lipid peroxidation; (*n* = 3 biological replicates in either single or technical duplicate). The RSL3- or erastin-treated condition was used as comparator in the statistical analysis. (**E,F**) Human BT20 poorly metastasizing cells were pre-treated with 10 μM FADS2i (SC-26196), FADS1/2i (CP-24879), and SCD1i (CAY10566) for 4 hours, then administrated (i) ON with 0.25 μM RSL3 (E, left) or 0.5 μM erastin (F, left) before being subjected to cell viability assay, and (ii) for 2 hours with 1 μM RSL3 (E, right) or 5 μM erastin (F, right) before being subjected to cytofluorimetric analysis to measure lipid peroxidation; (*n* = 3 biological replicates). The RSL3- or erastin-treated condition was used as comparator in the statistical analysis. (**G,H**) Human HCC1937 poorly metastasizing cells were pre-treated with 10 μM FADS2i (SC-26196), FADS1/2i (CP-24879), and SCD1i (CAY10566) for 4 hours, then administrated (i) ON with 0.25 μM RSL3 (G, left) or 0.5 μM erastin (H, left) before being subjected to cell viability assay, or (ii) for 2 hours with 1 μM RSL3 (G, right) or 5 μM erastin (H, right) before being subjected to cytofluorimetric analysis to measure lipid peroxidation; (*n* = 3 biological replicates). The RSL3- or erastin-treated condition was used as comparator in the statistical analysis. Data information: data are presented as mean ± SEM. Statistical analysis was performed using One-way ANOVA followed by Dunnett's correction.

Appendix Figure S3

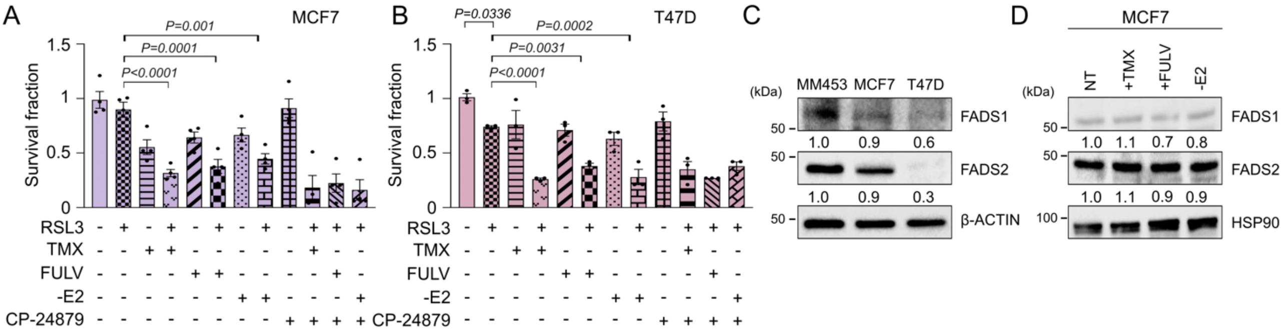

Appendix Figure S3. Impairing Estrogen Receptor (ER) activity in ER+ breast cancer cells does not alter FADS1/2 expression.
(**A,B**) ER+ MCF7 (A) and T47D (B) breast cancer cells were pre-treated with 10 μM FADS1/2i (CP-24879) for 4 hours and then exposed to 1 μM RSL3, 1 μM TMX, 100 nM FULV, or subjected to estrogen deprivation (-E2) for 72 hours before cell viability assay; (*n* = 3 biological replicates in either single or technical duplicate). The RSL3-treated condition was used as comparator in the statistical analysis. (**C**) Total protein lysates from TNBC MDA-MB-453 and ER+ MCF7 and T47D cells were subjected to WB analysis with the antibodies indicated. (**D**) ER+ MCF7 breast cancer cells were treated with 1 μM TMX, 100 nM FULV, or subjected to estrogen deprivation (-E2) for 72 hours and then analyzed by WB analysis using the antibodies described in the figure. Data information: In (A,B) data are presented as mean ± SEM. Statistical analysis was performed using One-way ANOVA followed by Dunnett's correction.
