# Supplementary material for: FADS1/2 control lipid metabolism and ferroptosis susceptibility in triple-negative breast cancer
Source: EMBO Mol Med. 2024 Jun 26;16(7):5. doi: 10.1038/s44321-024-00090-6 (PMC11251055; doi:10.1038/s44321-024-00090-6)
Supplement: Supplementary file 5 — Source data Fig. 1 [file 44321_2024_90_MOESM5_ESM.zip › Figure 1/1E.pptx]

## Slide 1
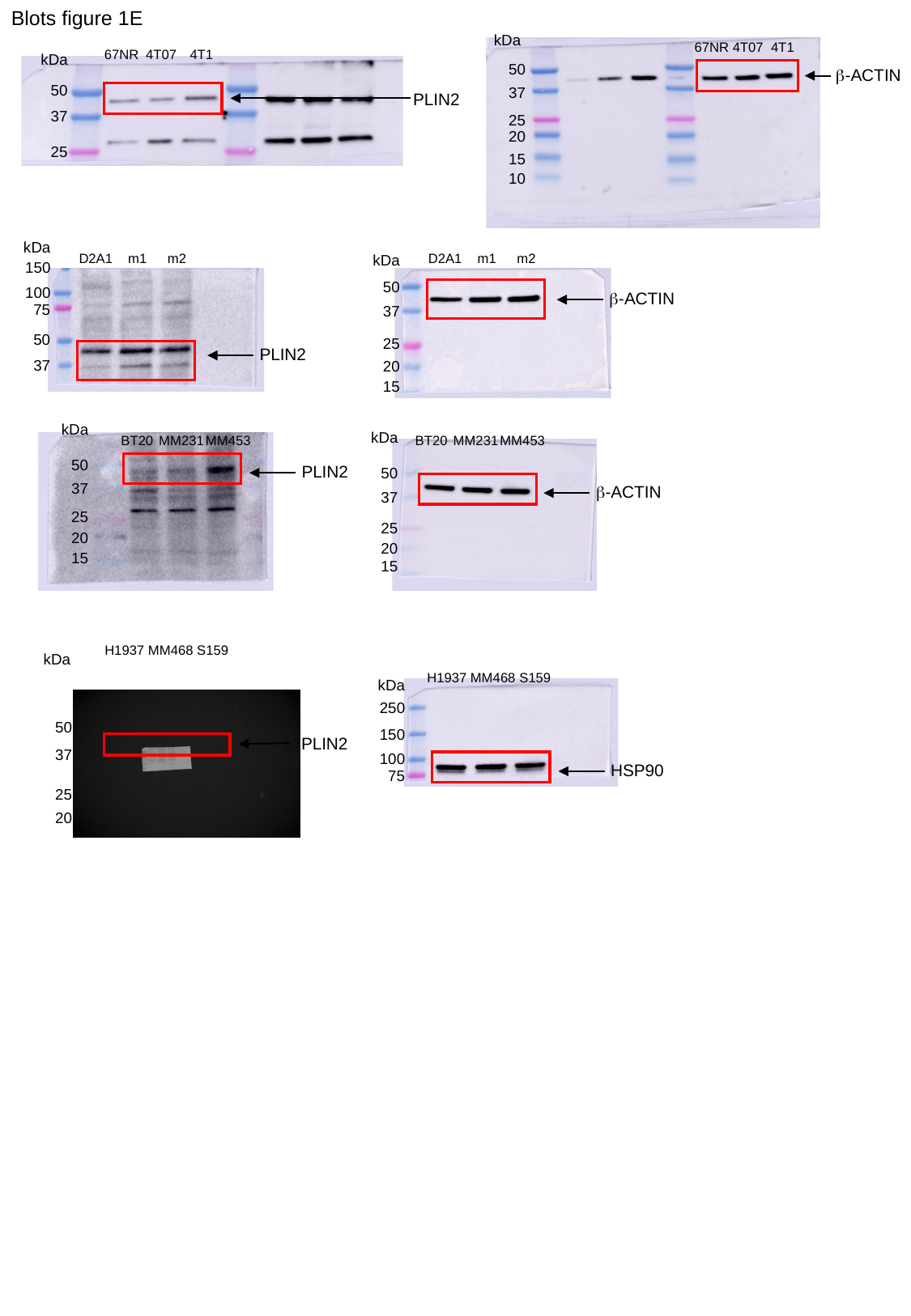

Blots figure 1E
kDa
67NR
4T07
4T1
67NR
4T07
4T1
kDa
50
β-ACTIN
50
37
PLIN2
37
25
20
25
15
10
kDa
D2A1
m1
m2
D2A1
m1
m2
kDa
150
50
100
β-ACTIN
75
37
50
25
PLIN2
37
20
15
kDa
kDa
BT20
MM231
MM453
BT20
MM231
MM453
50
PLIN2
50
37
β-ACTIN
37
25
25
20
20
15
15
H1937
MM468
S159
kDa
H1937
MM468
S159
kDa
250
50
150
PLIN2
37
100
HSP90
75
25
20
