# Supplementary material for: FADS1/2 control lipid metabolism and ferroptosis susceptibility in triple-negative breast cancer
Source: EMBO Mol Med. 2024 Jun 26;16(7):5. doi: 10.1038/s44321-024-00090-6 (PMC11251055; doi:10.1038/s44321-024-00090-6)
Supplement: Supplementary file 5 — Source data Fig. 1 [file 44321_2024_90_MOESM5_ESM.zip › Figure 1/1C.pptx]

## Slide 1
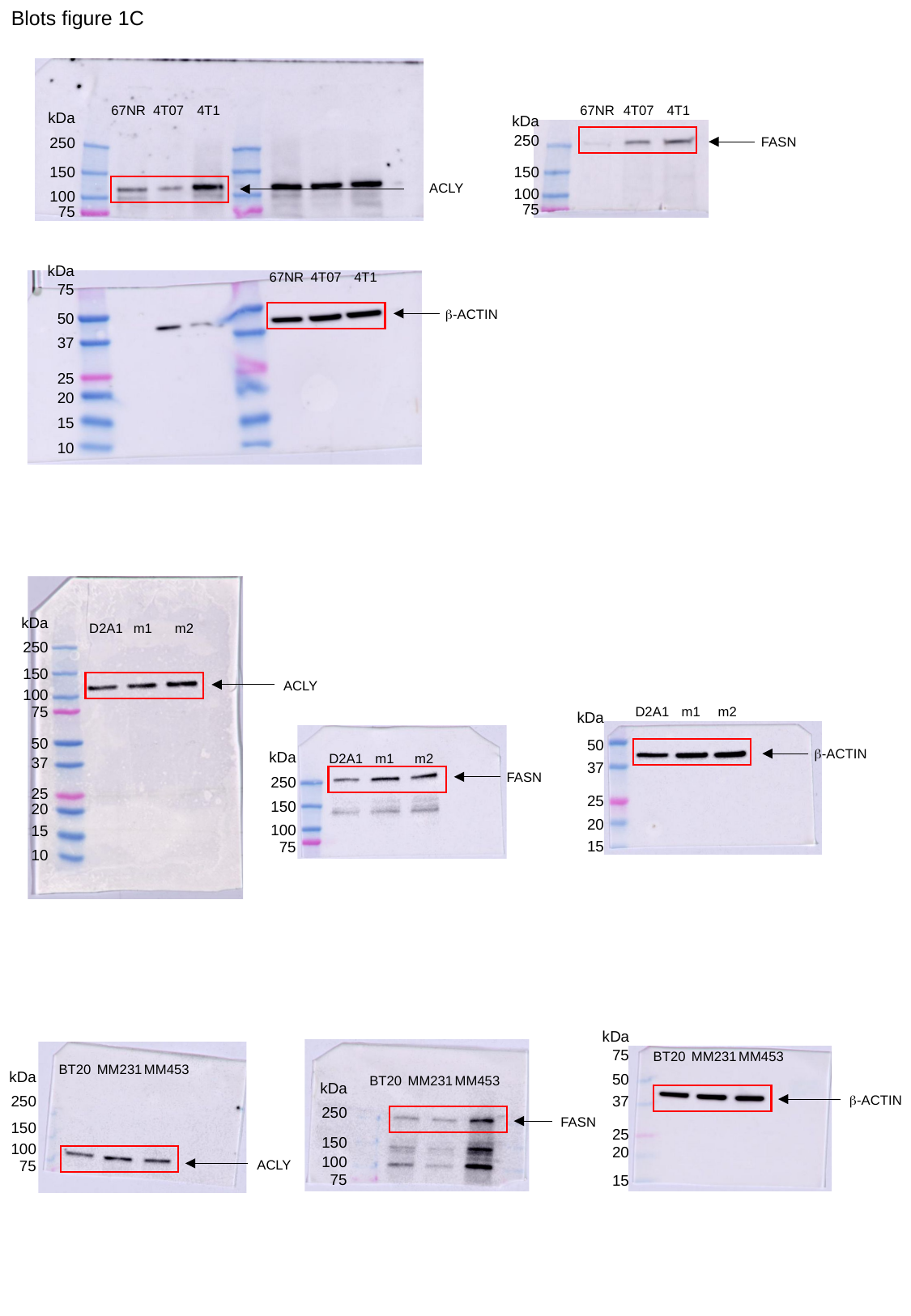

Blots figure 1C
67NR
4T07
4T1
kDa
250
150
ACLY
100
75
67NR
4T07
4T1
kDa
250
FASN
150
100
75
kDa
67NR
4T07
4T1
75
50
37
25
20
15
10
β-ACTIN
kDa
D2A1
m1
m2
250
150
ACLY
100
75
50
37
25
20
15
10
D2A1
m1
m2
kDa
50
37
25
20
15
β-ACTIN
kDa
D2A1
m1
m2
FASN
250
150
100
75
kDa
75
BT20
MM231
MM453
50
β-ACTIN
37
25
20
15
BT20
MM231
MM453
kDa
250
150
100
75
FASN
BT20
MM231
MM453
kDa
250
150
100
ACLY
75

## Slide 2
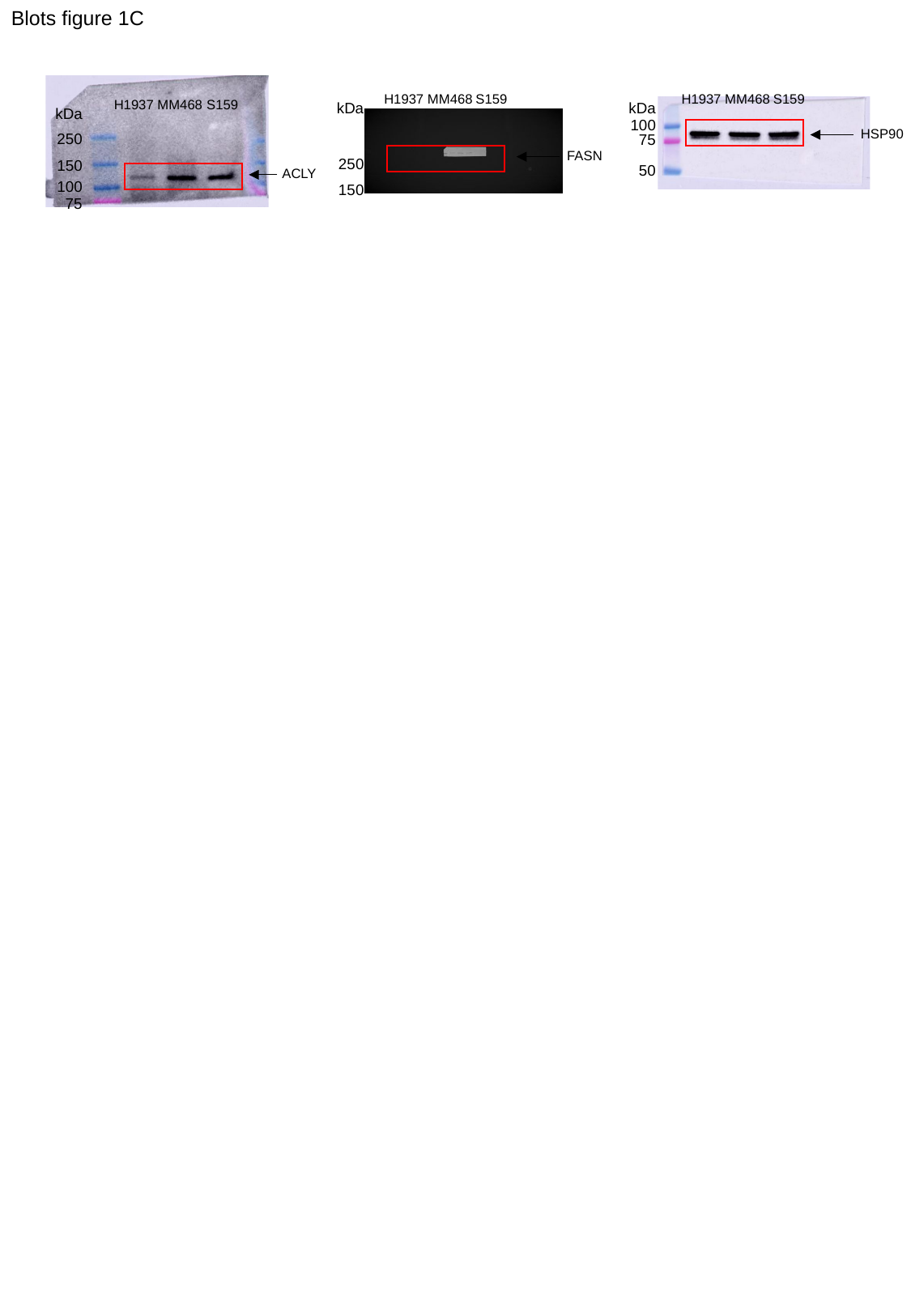

Blots figure 1C
H1937
MM468
S159
H1937
MM468
S159
H1937
MM468
S159
kDa
kDa
kDa
100
HSP90
250
75
FASN
250
150
50
ACLY
100
150
75
