# Supplementary figures and images for: FADS1/2 control lipid metabolism and ferroptosis susceptibility in triple-negative breast cancer
Source: EMBO Mol Med. 2024 Jun 26;16(7):5. doi: 10.1038/s44321-024-00090-6 (PMC11251055; doi:10.1038/s44321-024-00090-6)

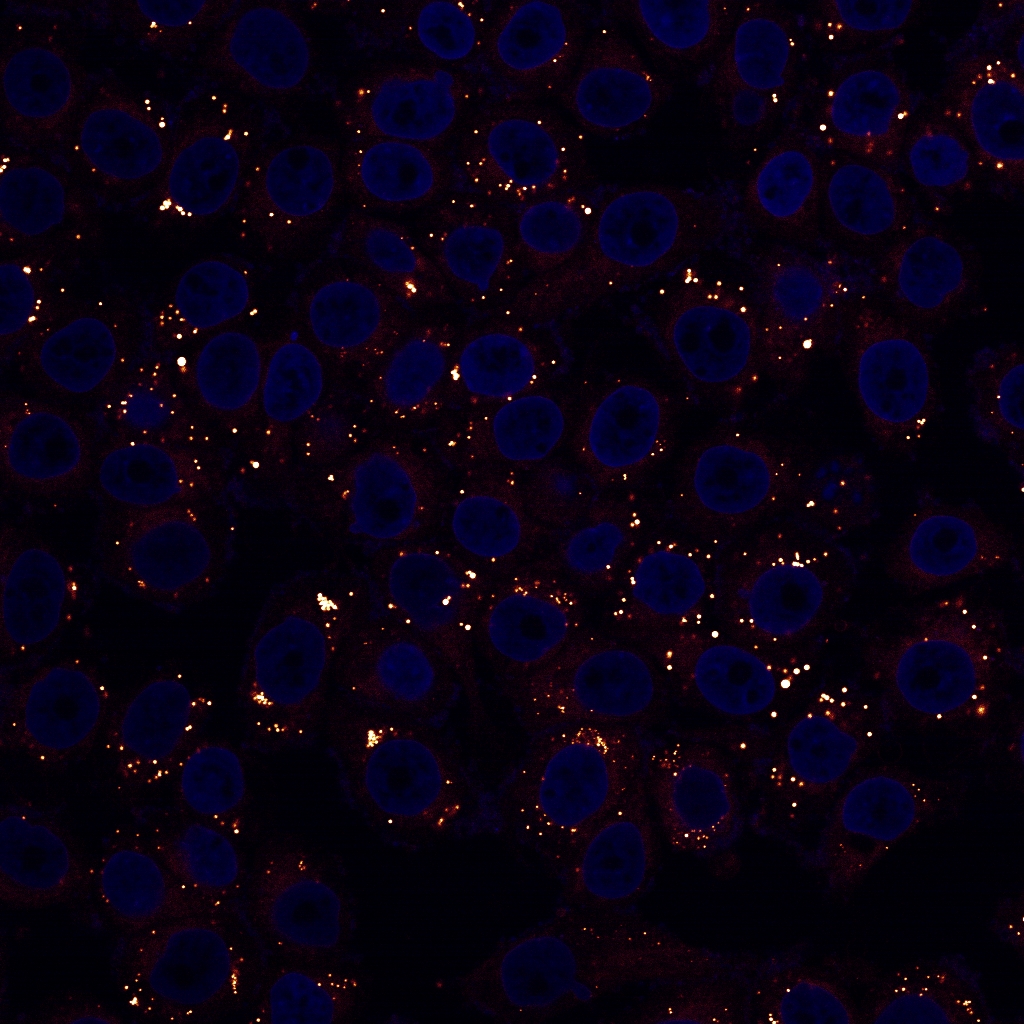

Supplement: Supplementary file 5 — Source data Fig. 1 [file 44321_2024_90_MOESM5_ESM.zip › Figure 1/1D/MDA-MB-468 LDs.jpg]

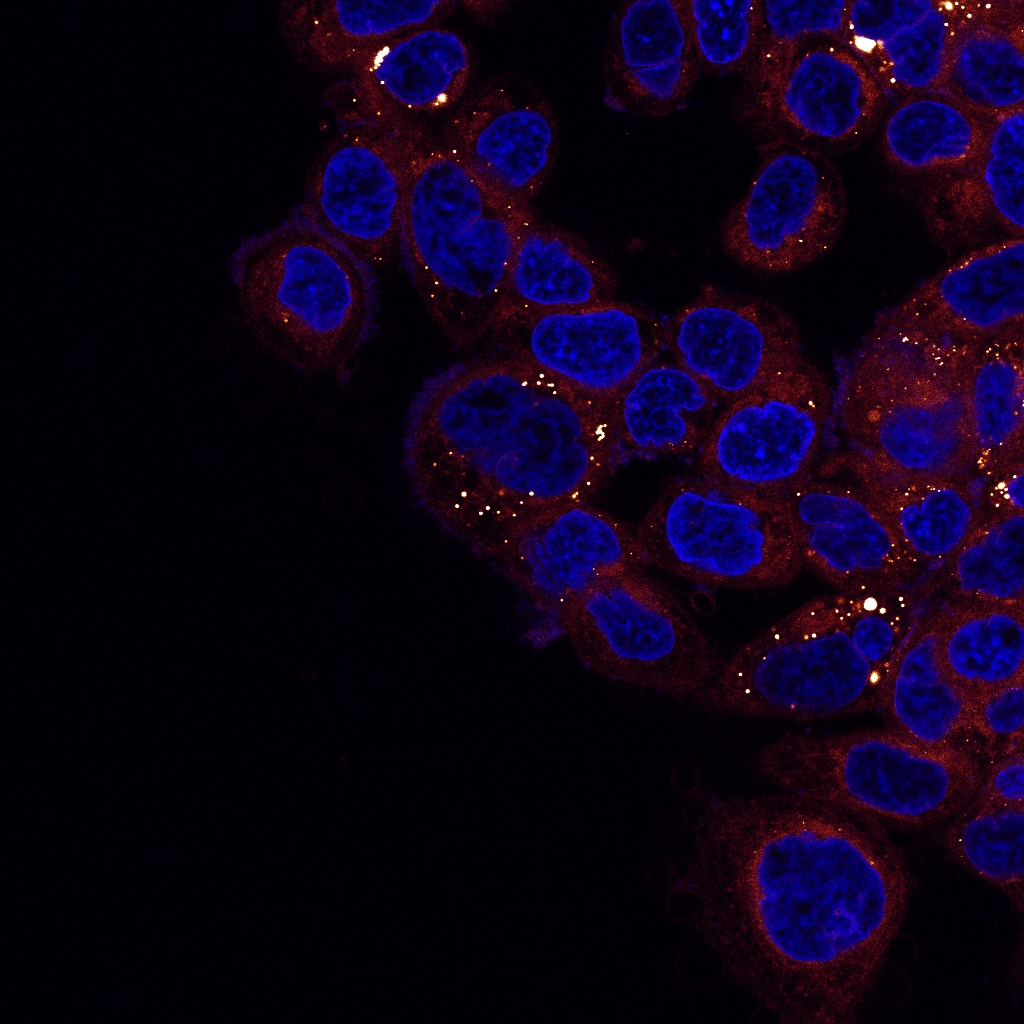

Supplement: Supplementary file 5 — Source data Fig. 1 [file 44321_2024_90_MOESM5_ESM.zip › Figure 1/1D/HCC1937 LDs.jpg]

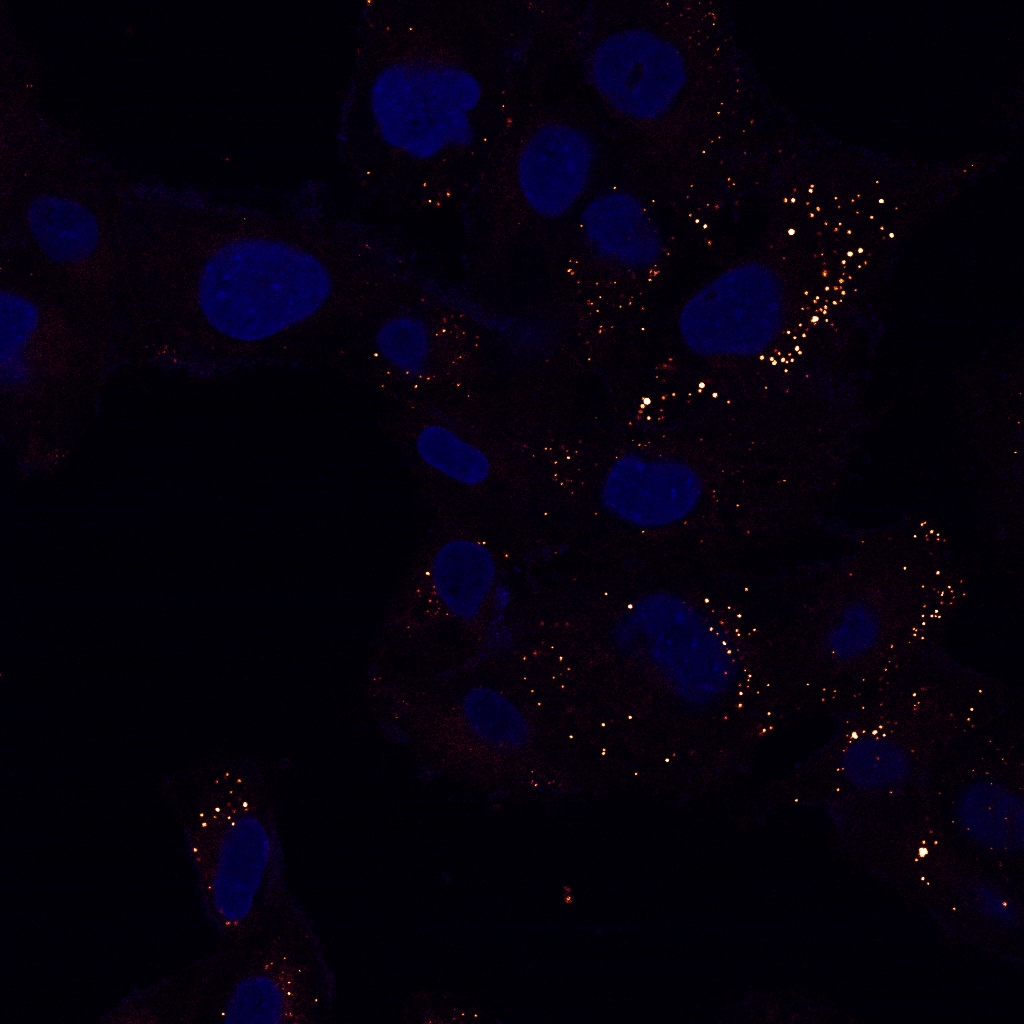

Supplement: Supplementary file 5 — Source data Fig. 1 [file 44321_2024_90_MOESM5_ESM.zip › Figure 1/1D/SUM159 LDs.jpg]

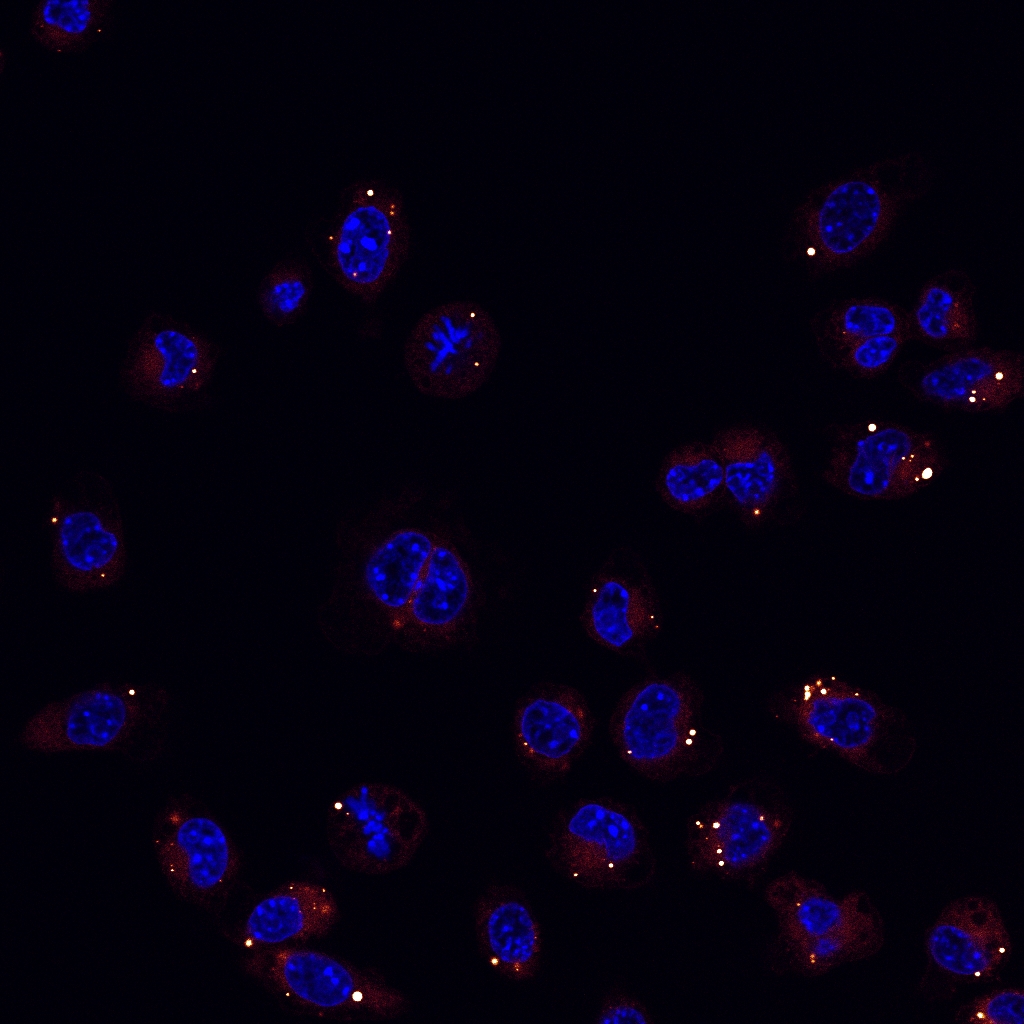

Supplement: Supplementary file 5 — Source data Fig. 1 [file 44321_2024_90_MOESM5_ESM.zip › Figure 1/1D/D2A1-m1 LDs.jpg]

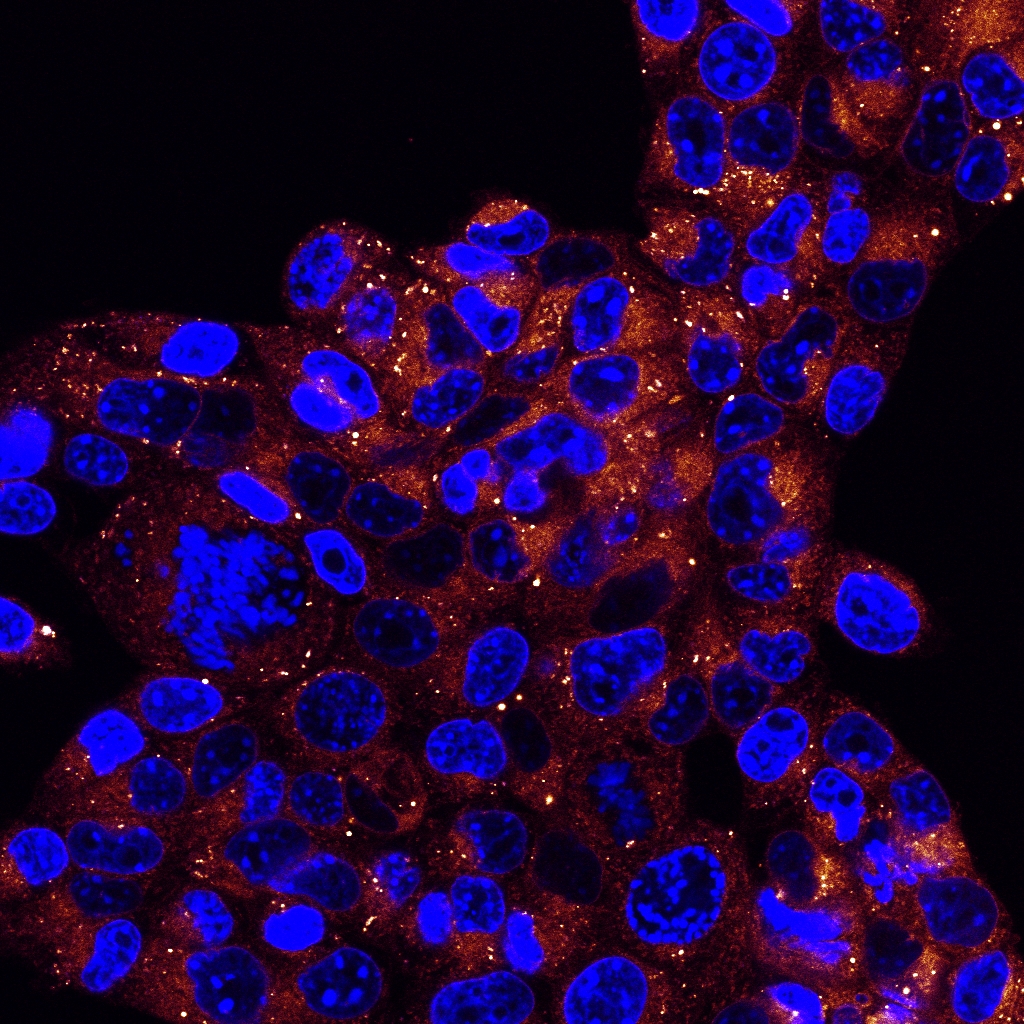

Supplement: Supplementary file 5 — Source data Fig. 1 [file 44321_2024_90_MOESM5_ESM.zip › Figure 1/1D/4T1 LDs.jpg]

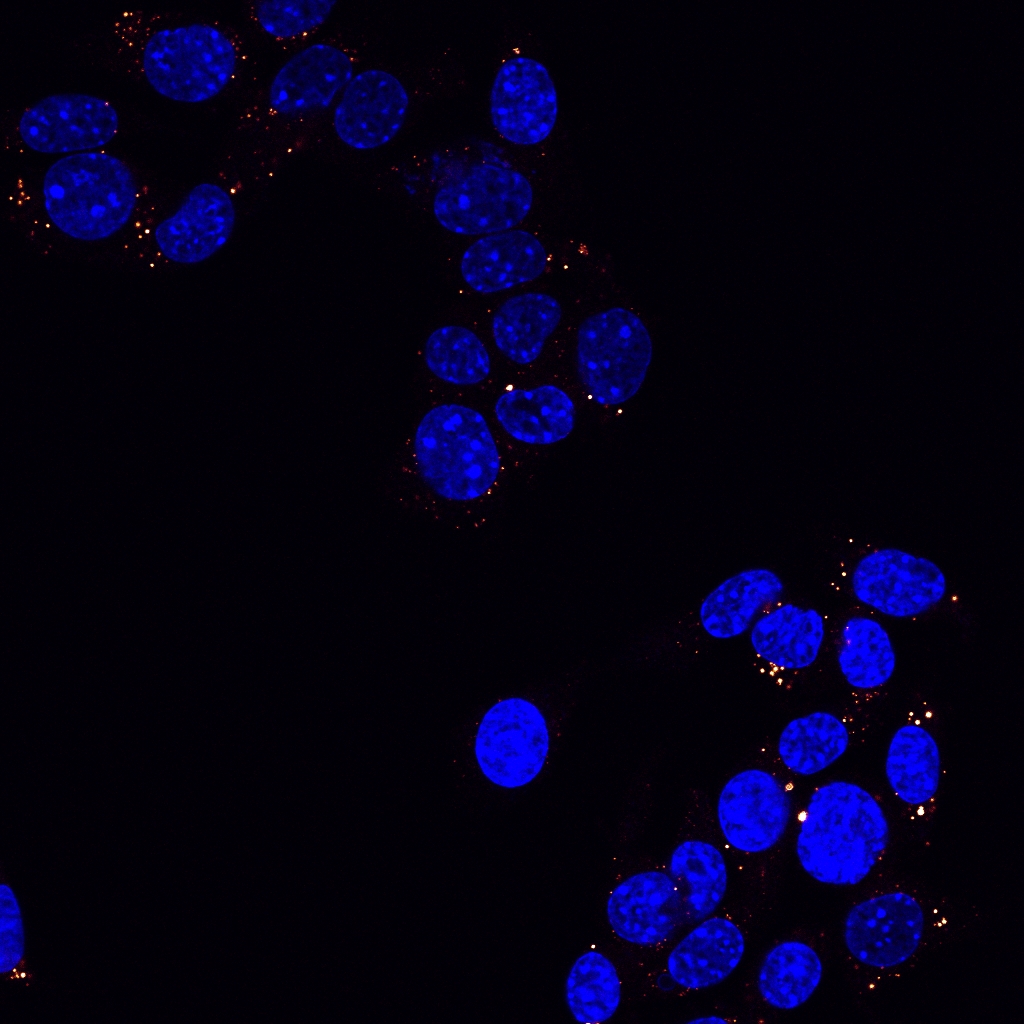

Supplement: Supplementary file 5 — Source data Fig. 1 [file 44321_2024_90_MOESM5_ESM.zip › Figure 1/1D/67NR LDs.jpg]

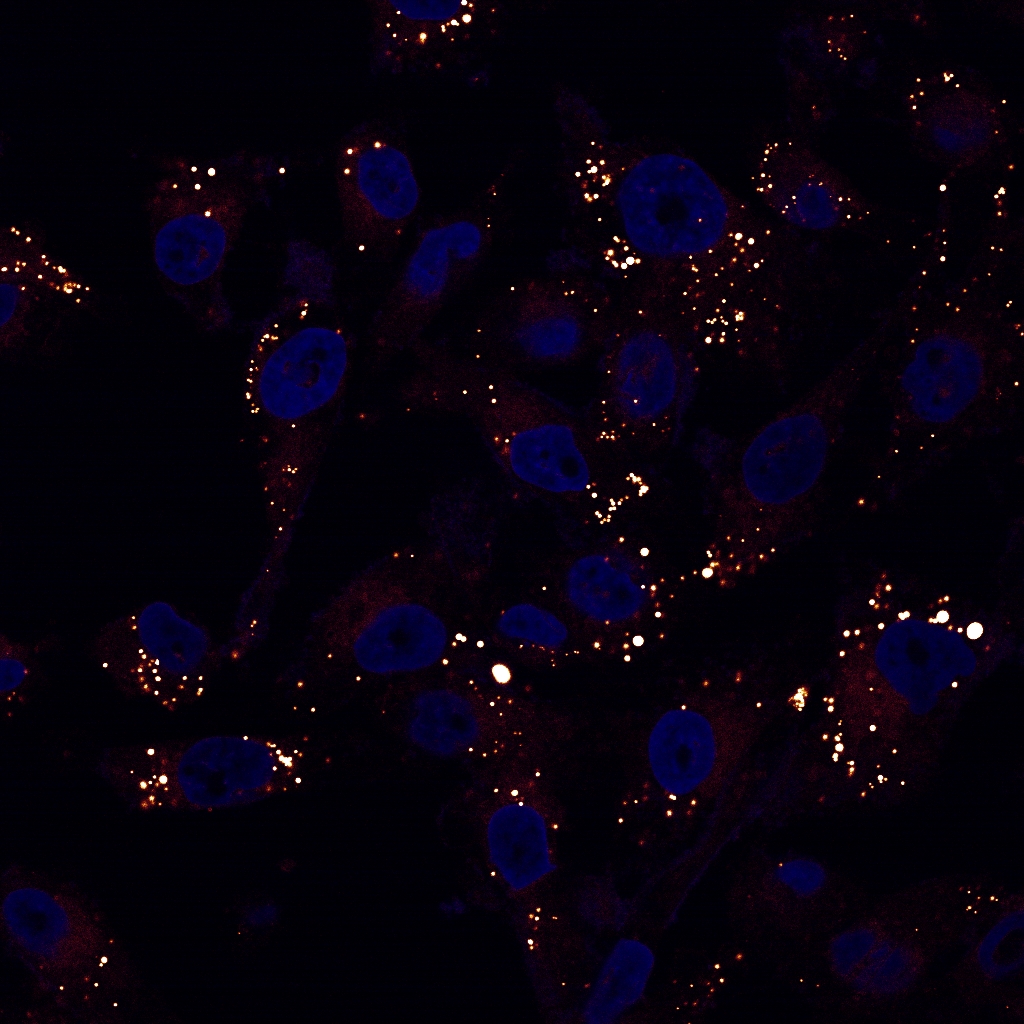

Supplement: Supplementary file 5 — Source data Fig. 1 [file 44321_2024_90_MOESM5_ESM.zip › Figure 1/1D/MDA-MB-231 LDs.jpg]

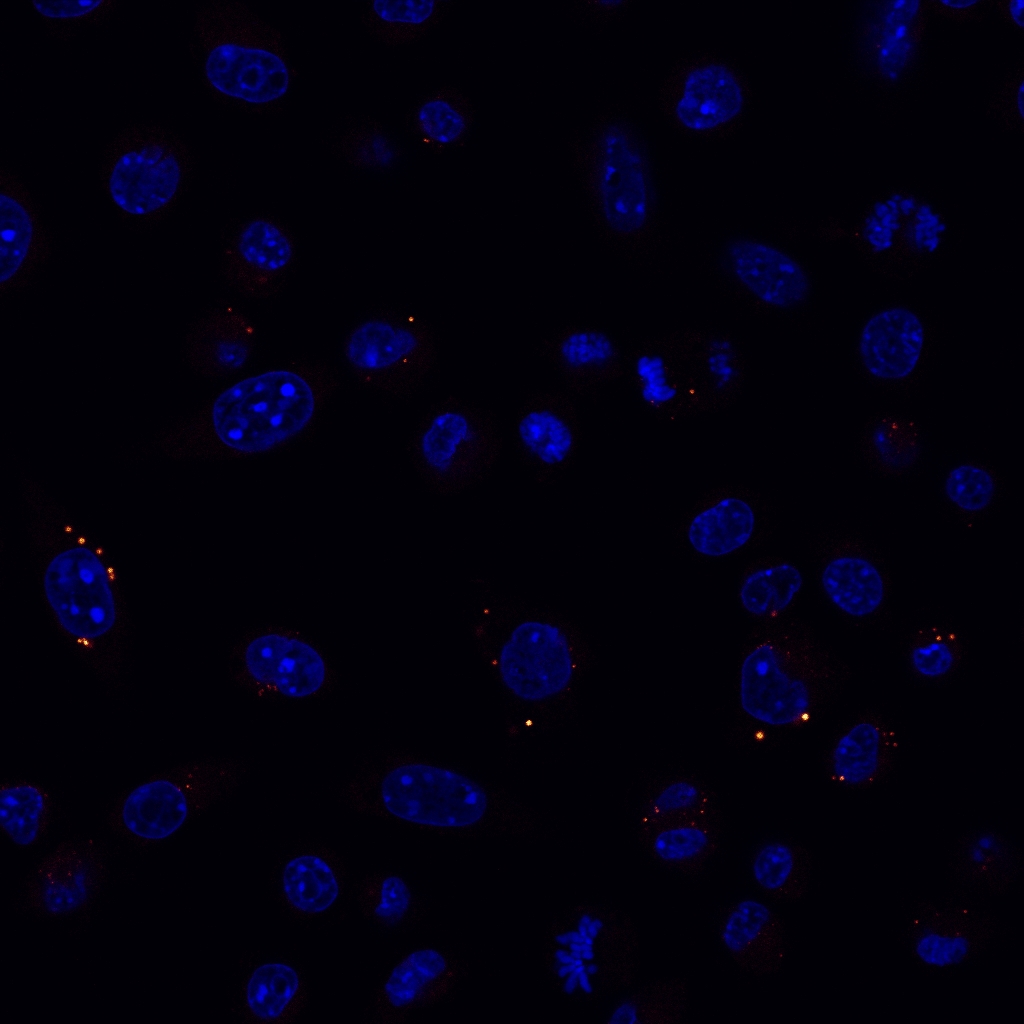

Supplement: Supplementary file 5 — Source data Fig. 1 [file 44321_2024_90_MOESM5_ESM.zip › Figure 1/1D/D2A1 LDs.jpg]

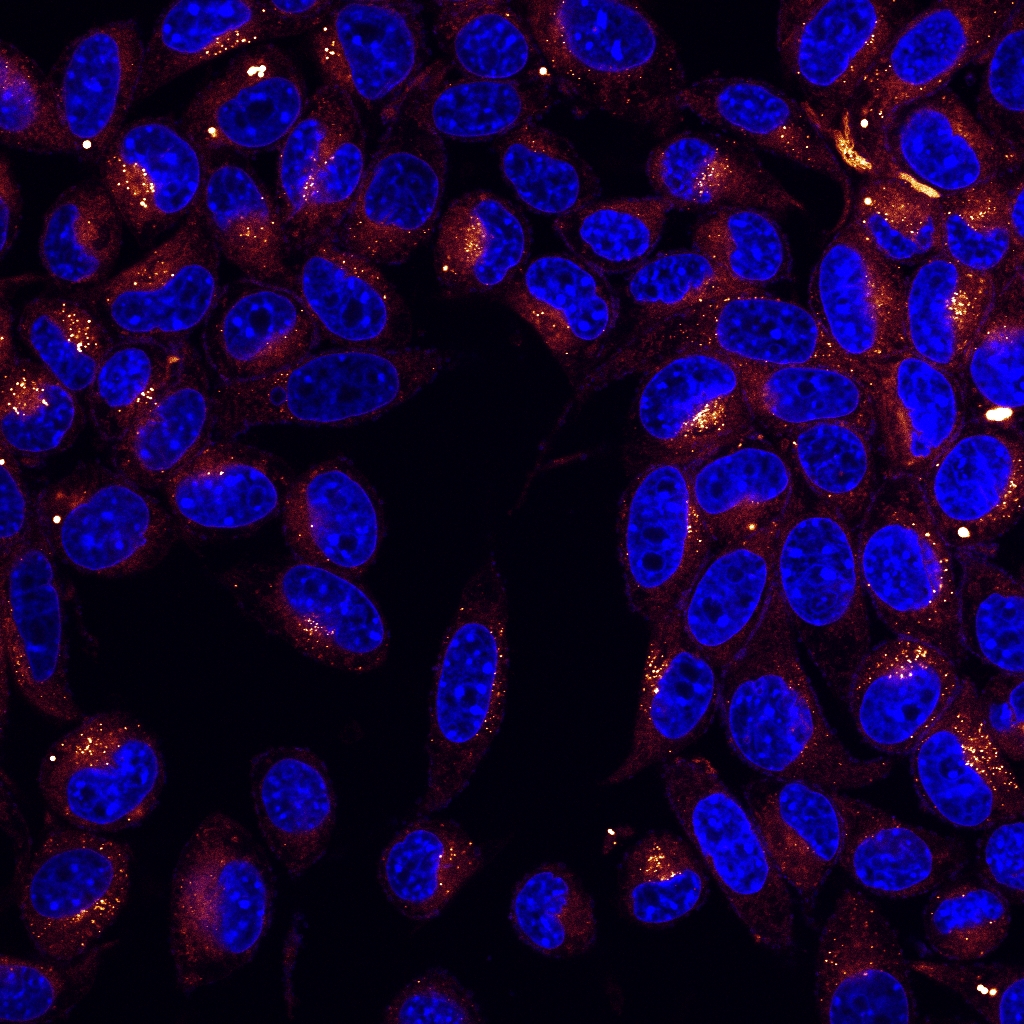

Supplement: Supplementary file 5 — Source data Fig. 1 [file 44321_2024_90_MOESM5_ESM.zip › Figure 1/1D/4T07 LDs.jpg]

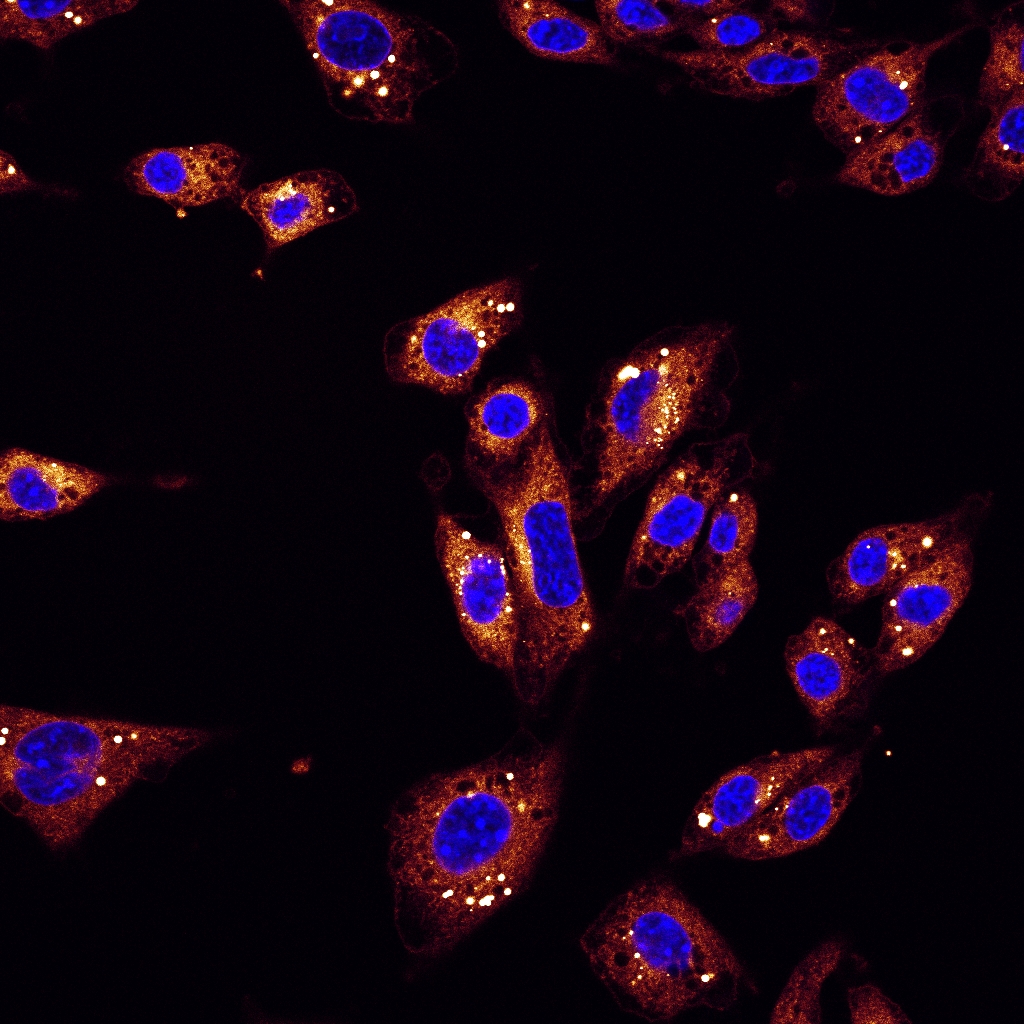

Supplement: Supplementary file 5 — Source data Fig. 1 [file 44321_2024_90_MOESM5_ESM.zip › Figure 1/1D/D2A1-m2 LDs.jpg]

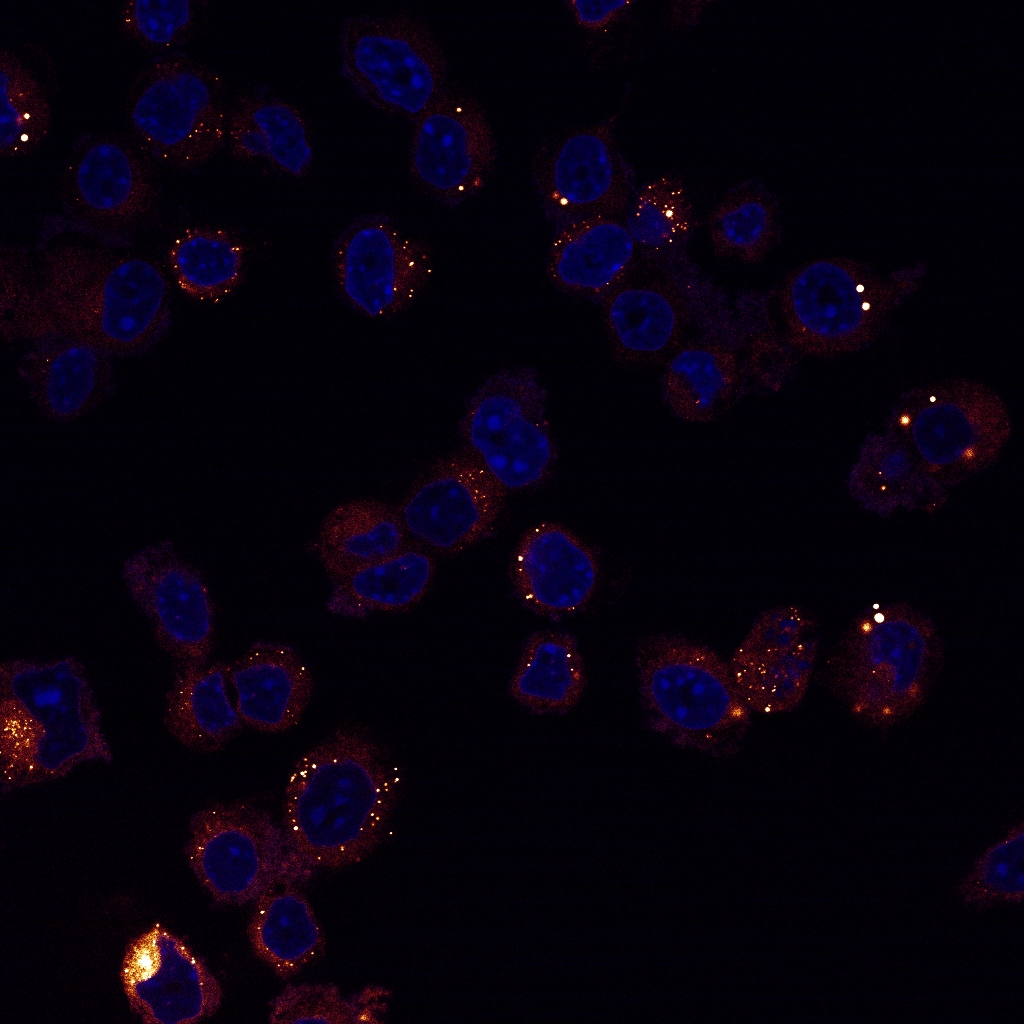

Supplement: Supplementary file 5 — Source data Fig. 1 [file 44321_2024_90_MOESM5_ESM.zip › Figure 1/1D/BT20 LDs.jpg]

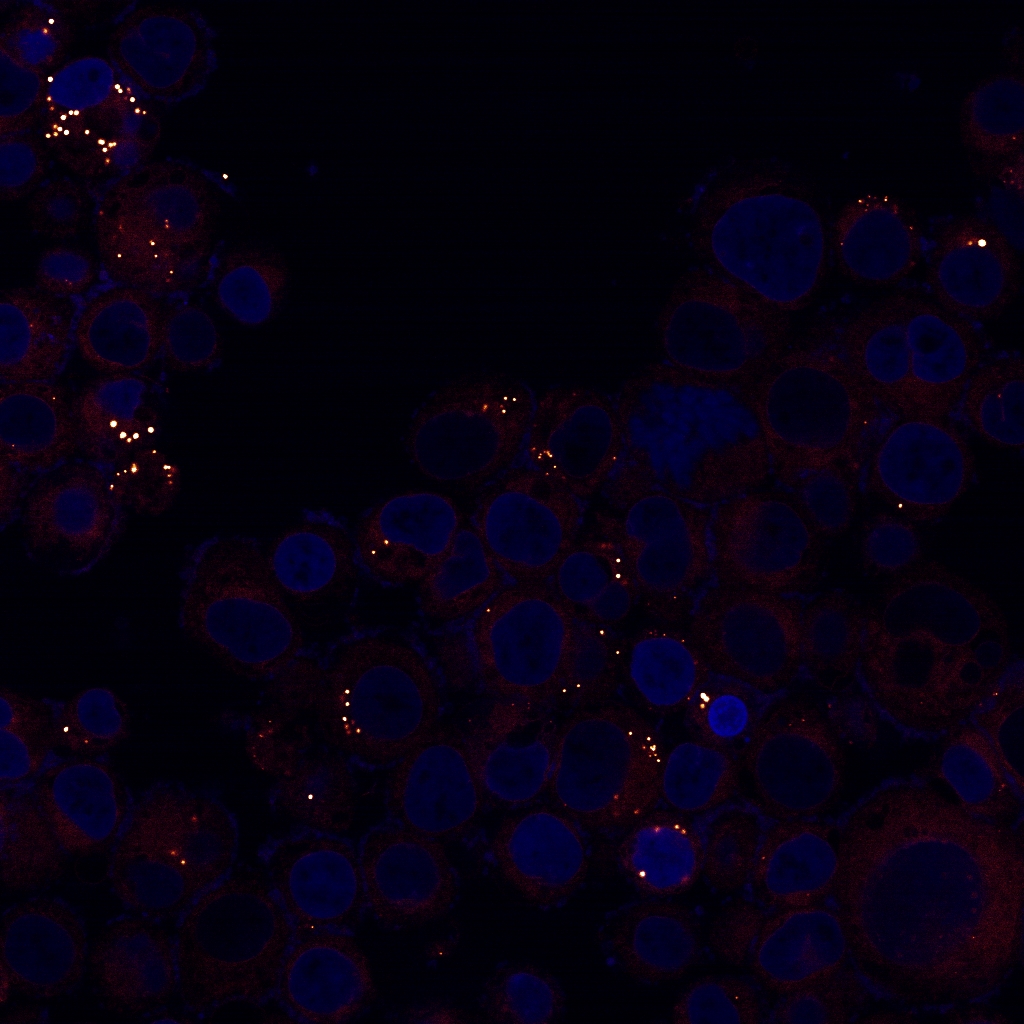

Supplement: Supplementary file 5 — Source data Fig. 1 [file 44321_2024_90_MOESM5_ESM.zip › Figure 1/1D/MDA-MB-453 LDs.jpg]

## Slide 1
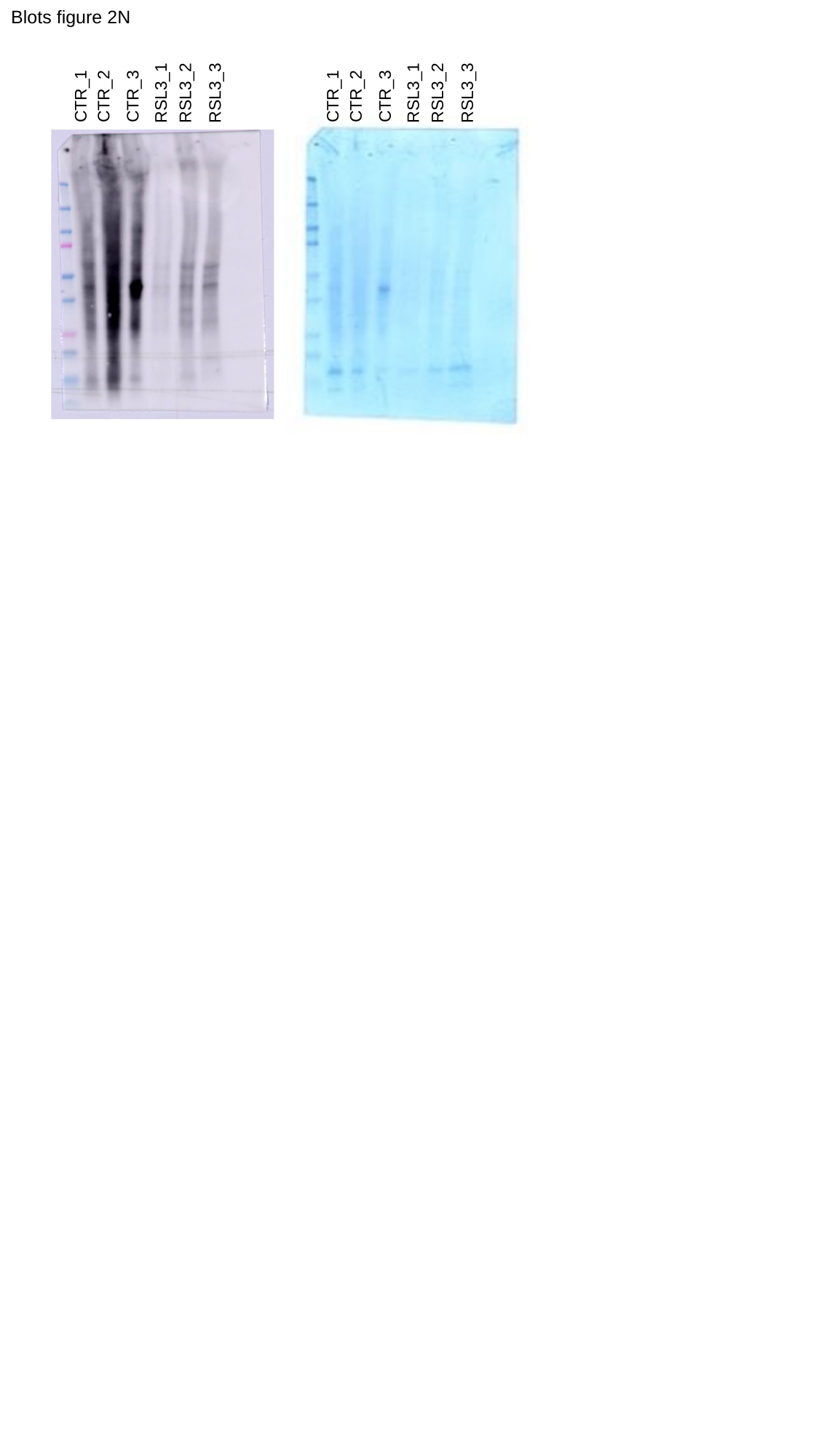

Blots figure 2N
RSL3_1
RSL3_2
RSL3_3
RSL3_1
RSL3_2
RSL3_3
CTR_1
CTR_2
CTR_3
CTR_1
CTR_2
CTR_3

Supplement: Supplementary file 6 — Source data Fig. 2 [file 44321_2024_90_MOESM6_ESM.zip › Figure 2/2N/2N.pptx]

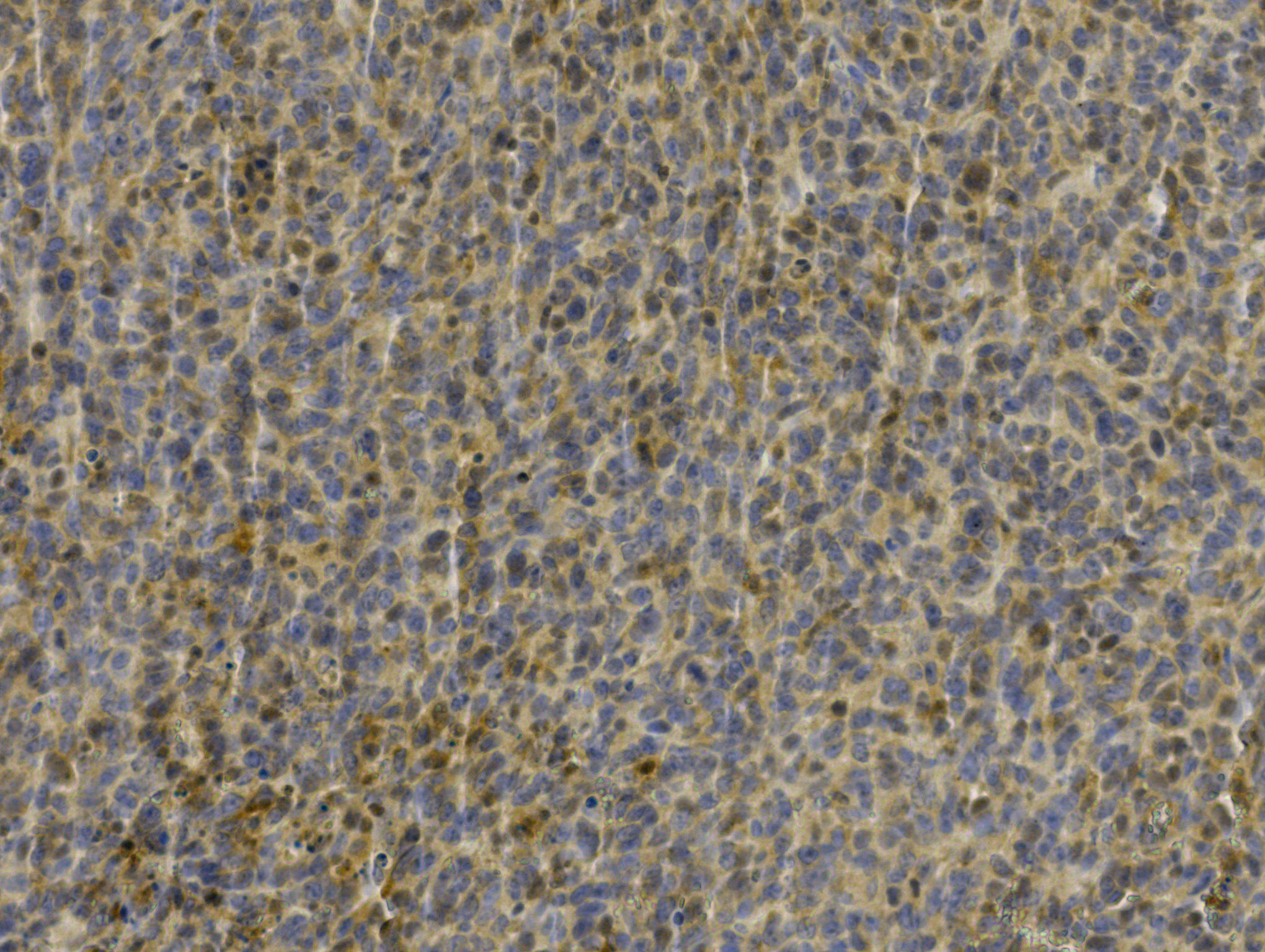

Supplement: Supplementary file 6 — Source data Fig. 2 [file 44321_2024_90_MOESM6_ESM.zip › Figure 2/2O/RSL3.tif]

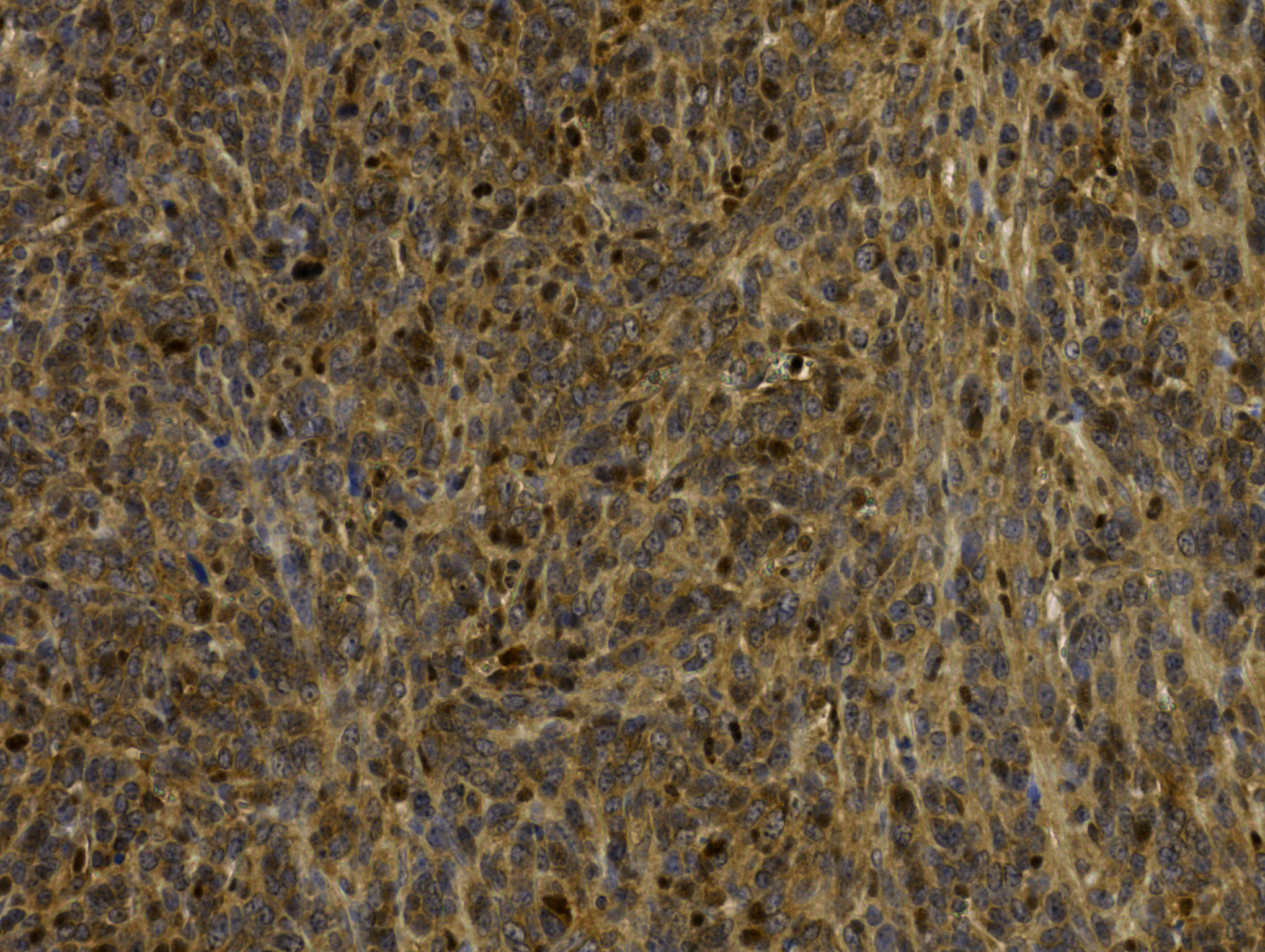

Supplement: Supplementary file 6 — Source data Fig. 2 [file 44321_2024_90_MOESM6_ESM.zip › Figure 2/2O/CTR.tif]

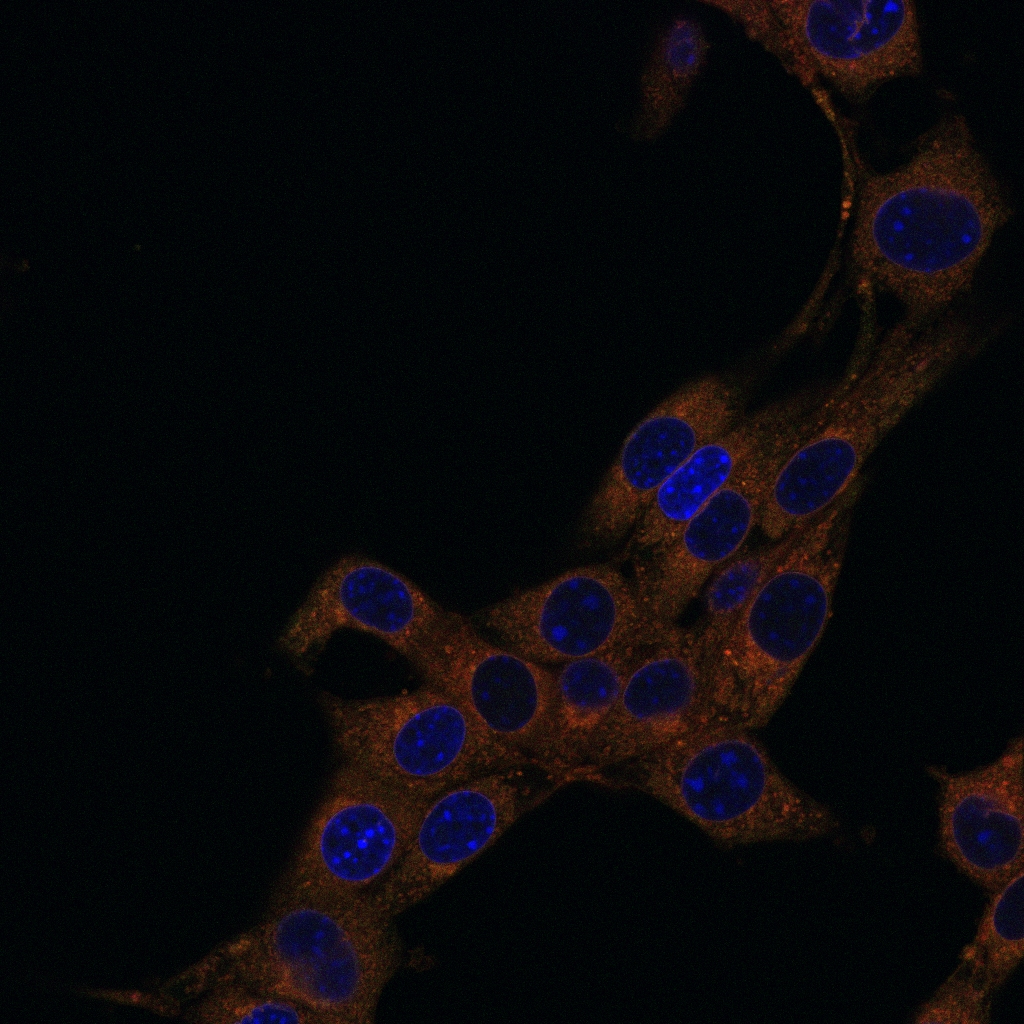

Supplement: Supplementary file 6 — Source data Fig. 2 [file 44321_2024_90_MOESM6_ESM.zip › Figure 2/2J/67NR NT C11.jpg]

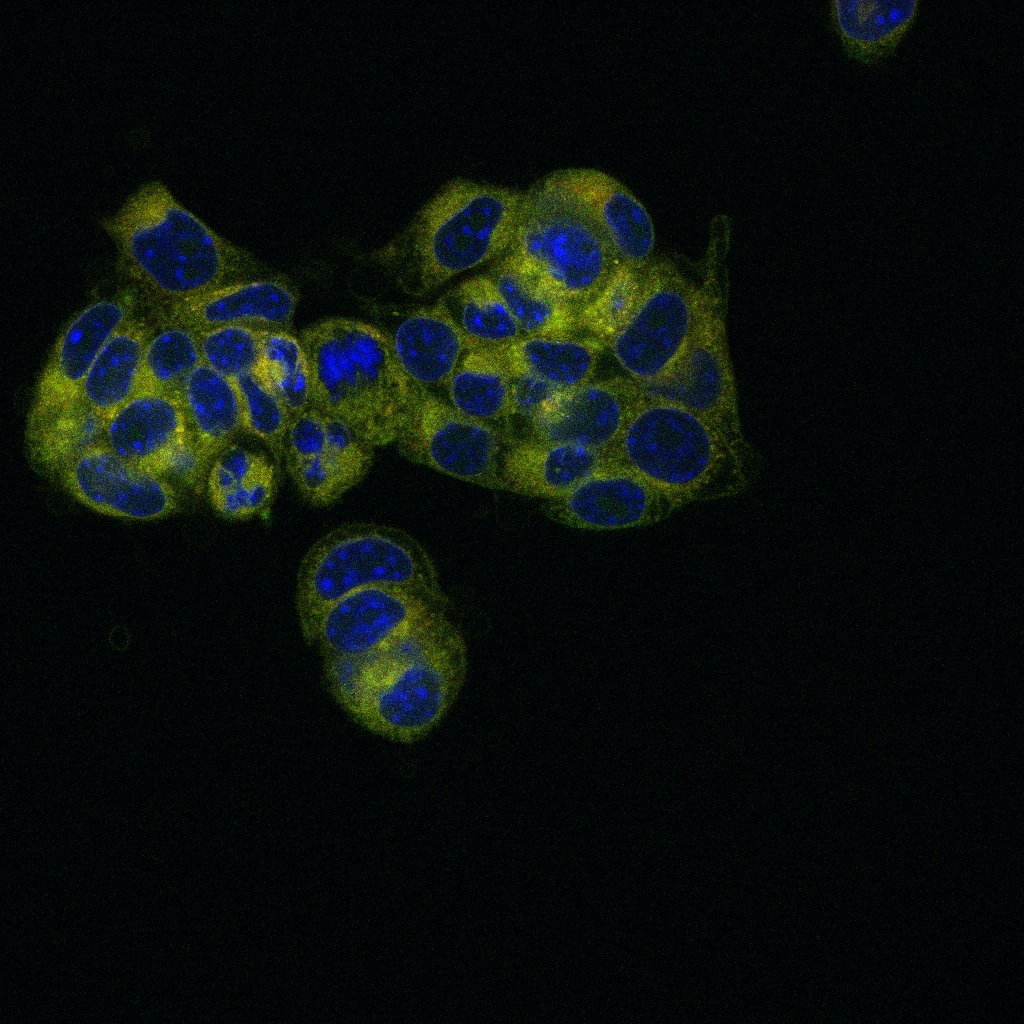

Supplement: Supplementary file 6 — Source data Fig. 2 [file 44321_2024_90_MOESM6_ESM.zip › Figure 2/2J/4T1 + RSL3 C11.jpg]

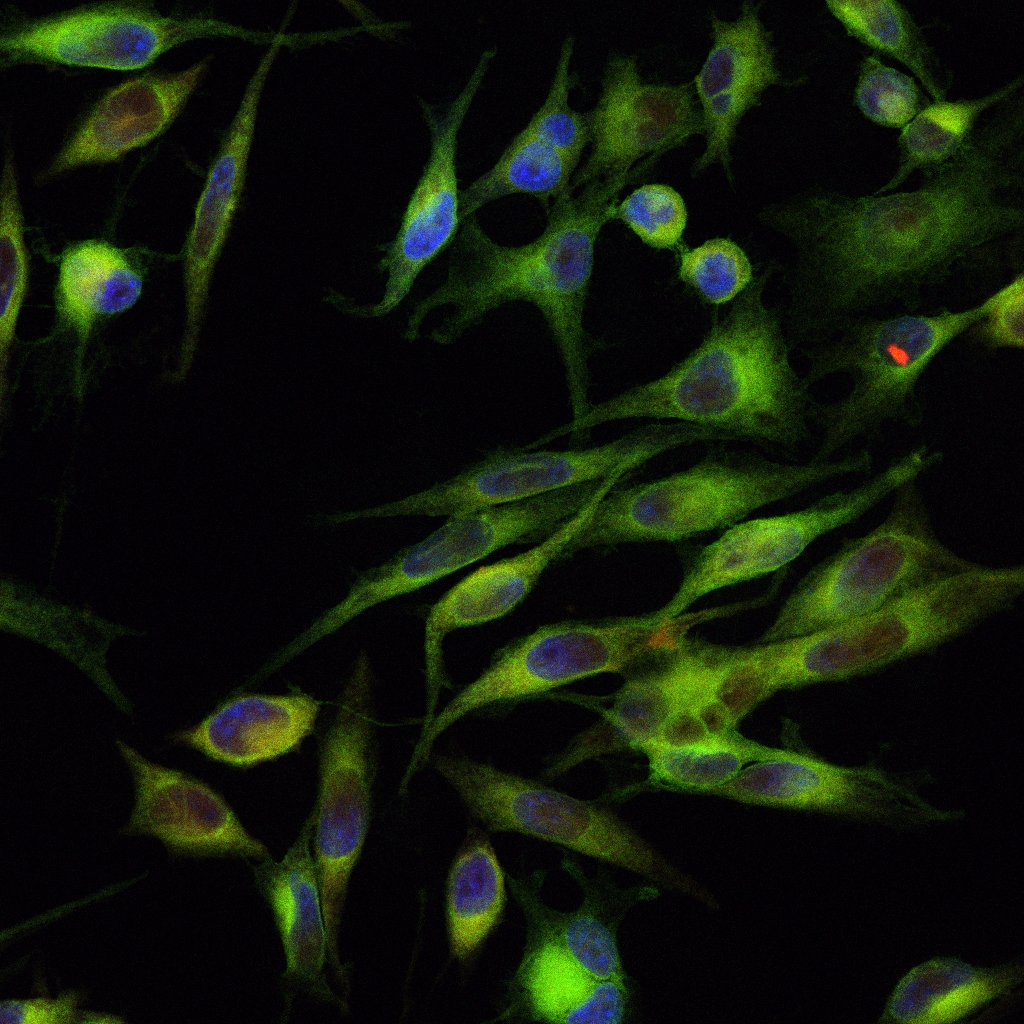

Supplement: Supplementary file 6 — Source data Fig. 2 [file 44321_2024_90_MOESM6_ESM.zip › Figure 2/2J/4T07 + RSL3 C11.jpg]

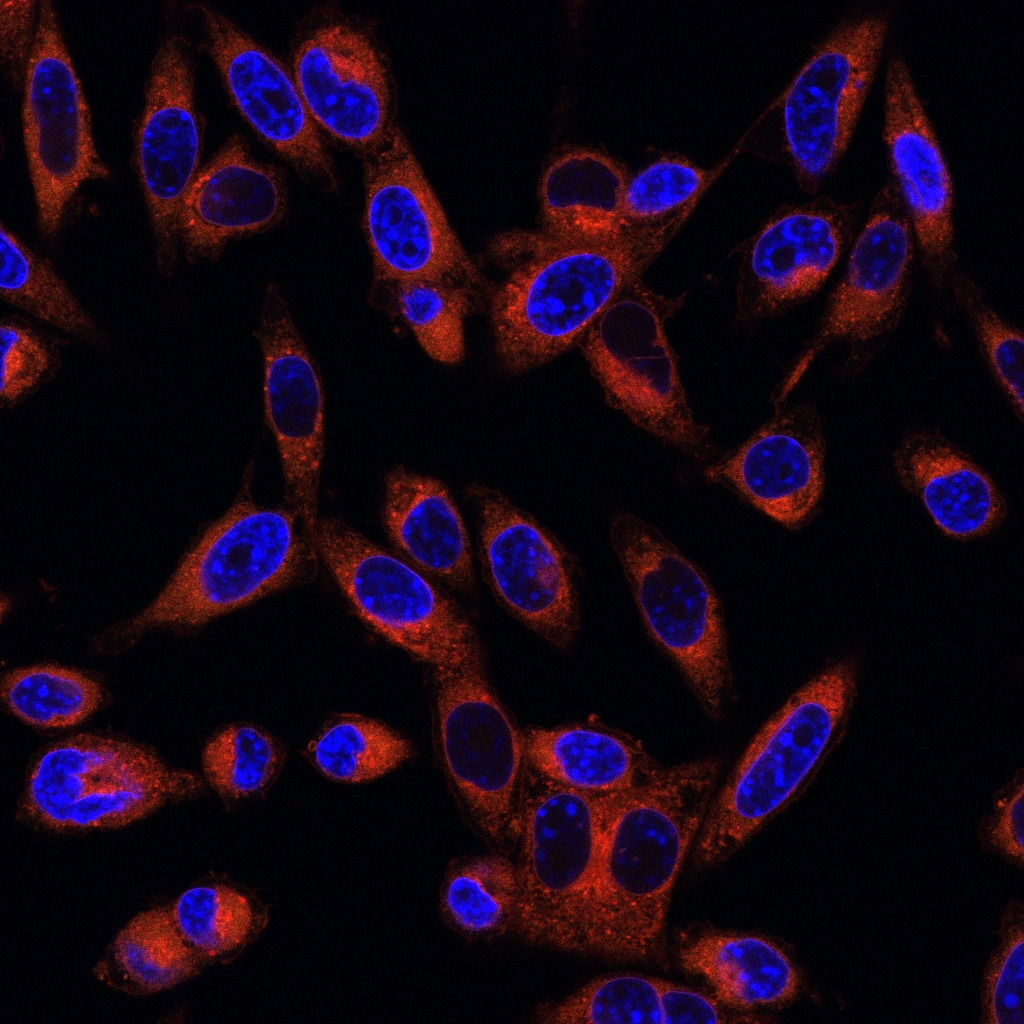

Supplement: Supplementary file 6 — Source data Fig. 2 [file 44321_2024_90_MOESM6_ESM.zip › Figure 2/2J/20220621_4T1 SERES_C11_+RSL3_4T07_NT_2.jpg]

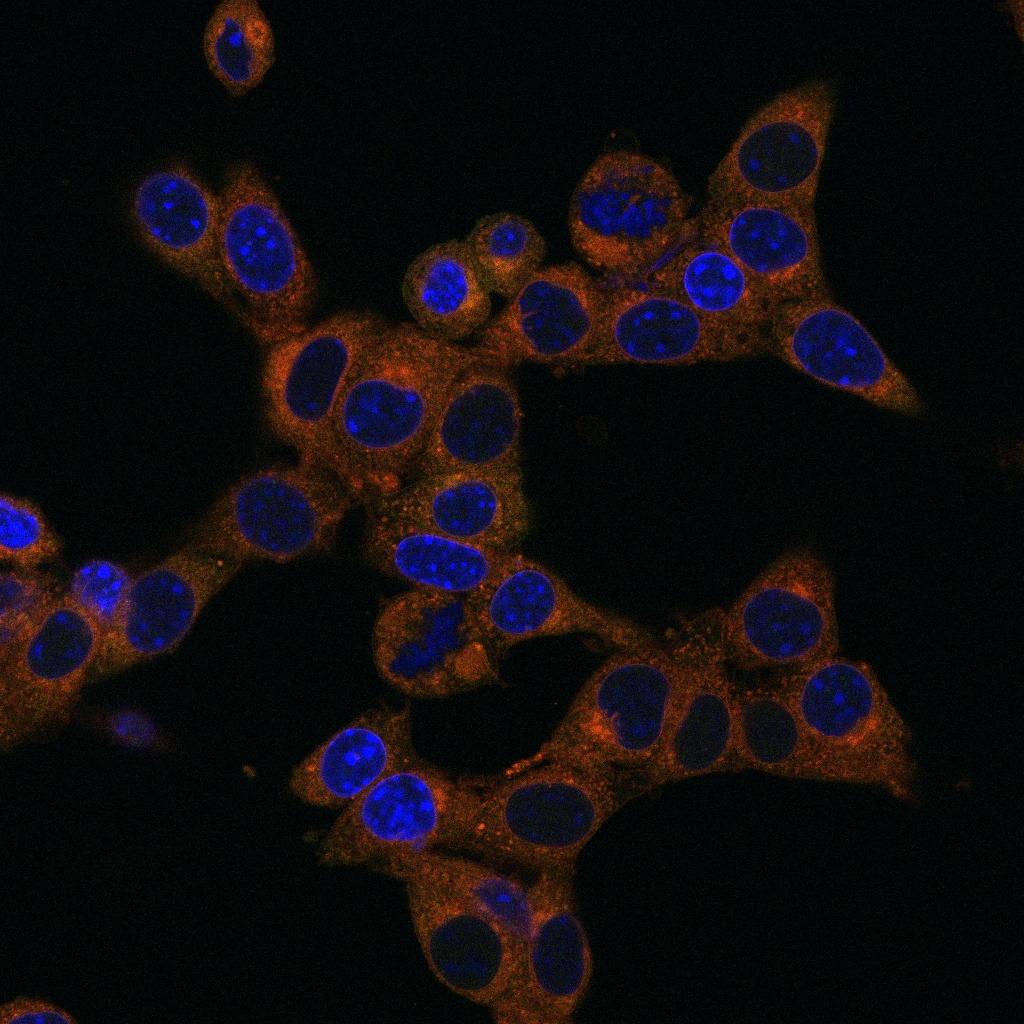

Supplement: Supplementary file 6 — Source data Fig. 2 [file 44321_2024_90_MOESM6_ESM.zip › Figure 2/2J/20220621_4T1 SERES_C11_+RSL3_67NR_+RSL3_1.jpg]

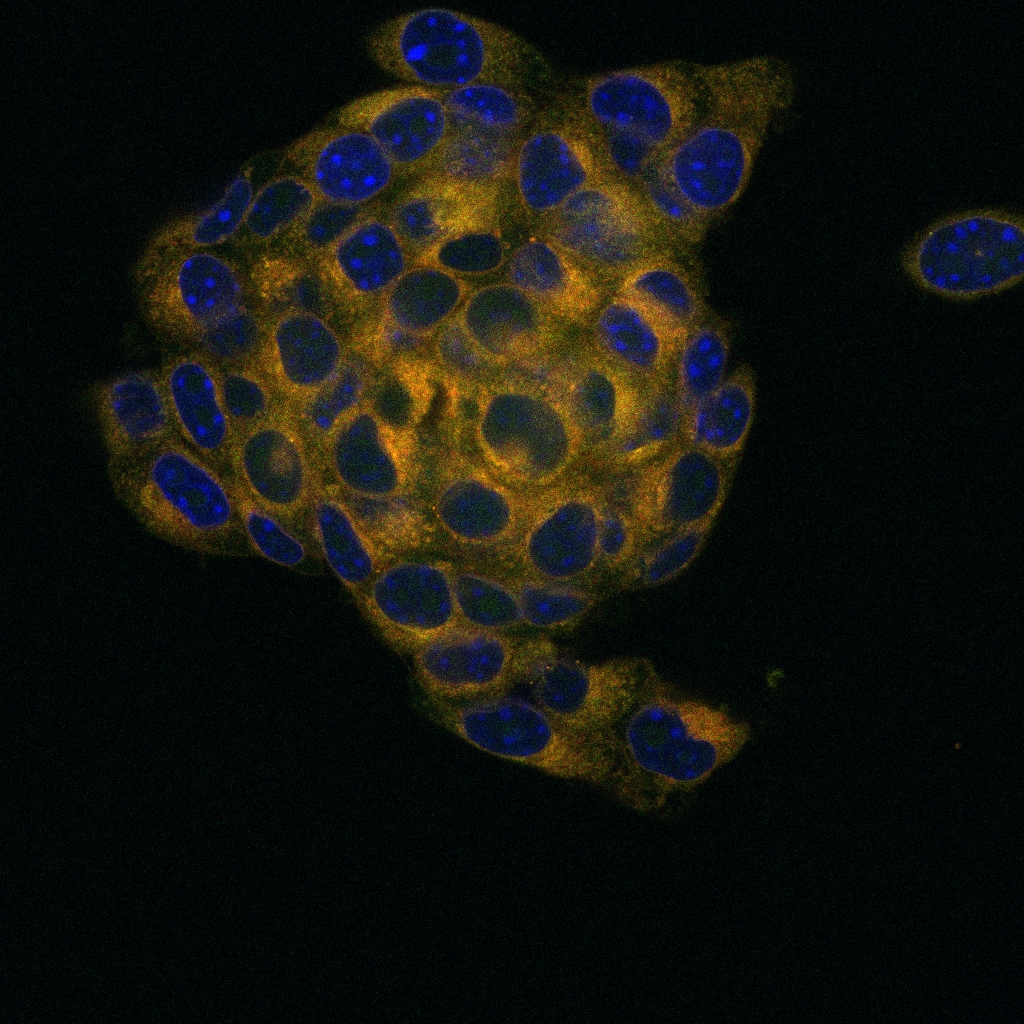

Supplement: Supplementary file 6 — Source data Fig. 2 [file 44321_2024_90_MOESM6_ESM.zip › Figure 2/2J/4T1 NT C11.jpg]

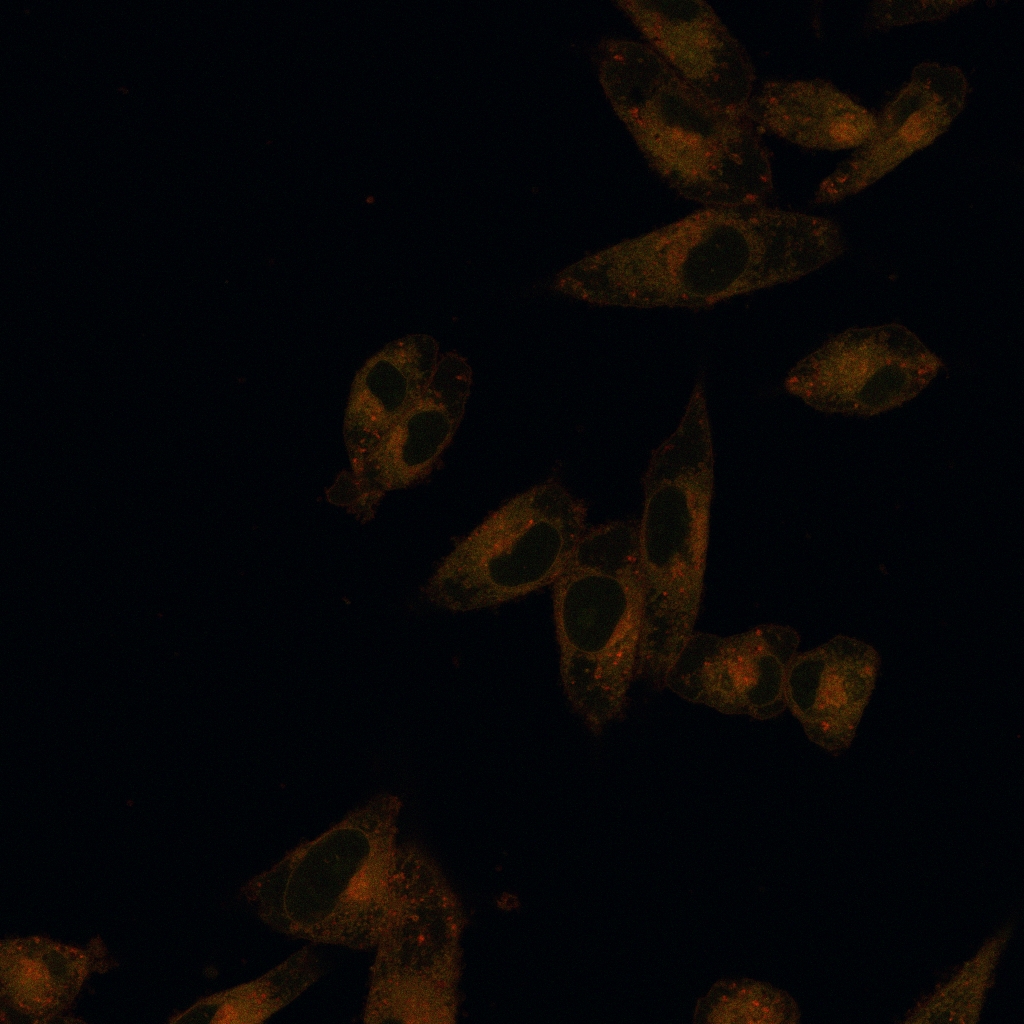

Supplement: Supplementary file 6 — Source data Fig. 2 [file 44321_2024_90_MOESM6_ESM.zip › Figure 2/2K/20230201_4T07_+ERASTIN 1-5micro_24h_C11_4T07_NT_2.jpg]

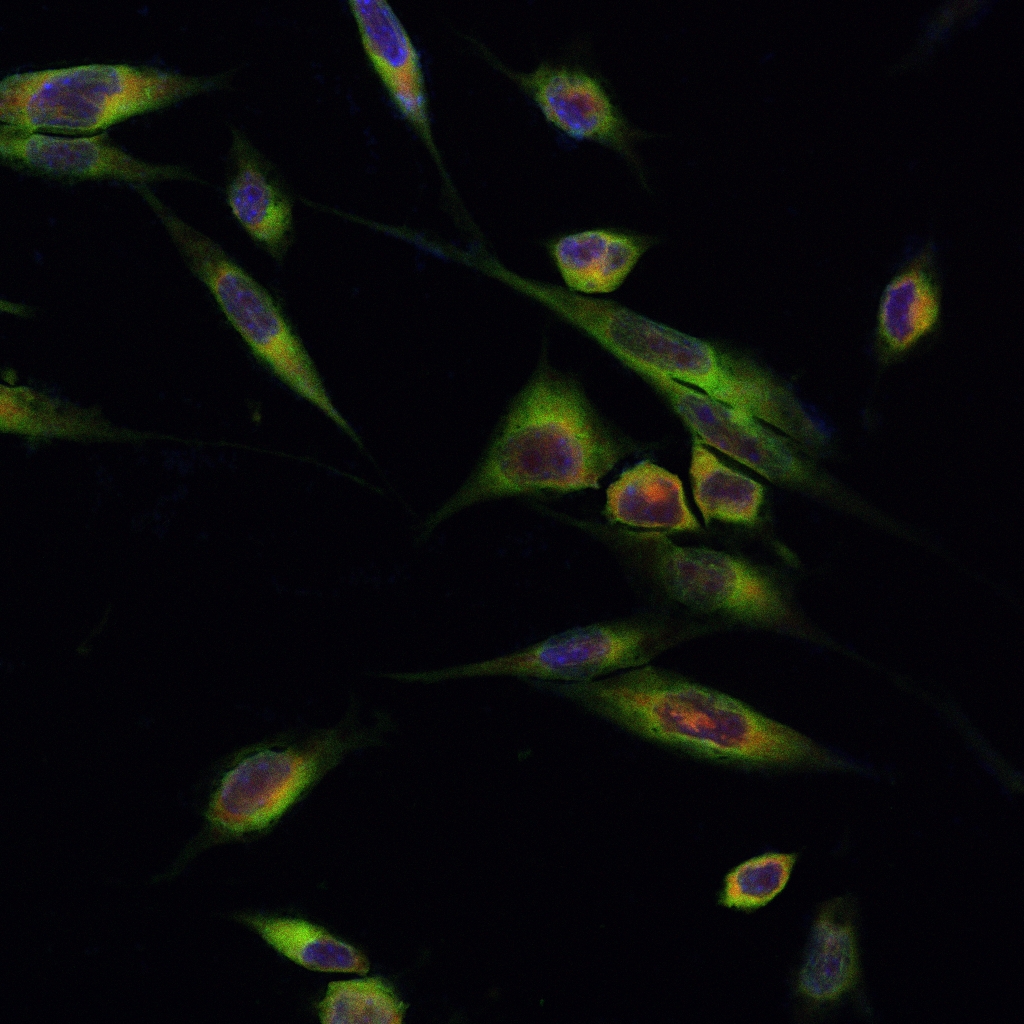

Supplement: Supplementary file 6 — Source data Fig. 2 [file 44321_2024_90_MOESM6_ESM.zip › Figure 2/2K/4T07 + erastin C11.jpg]

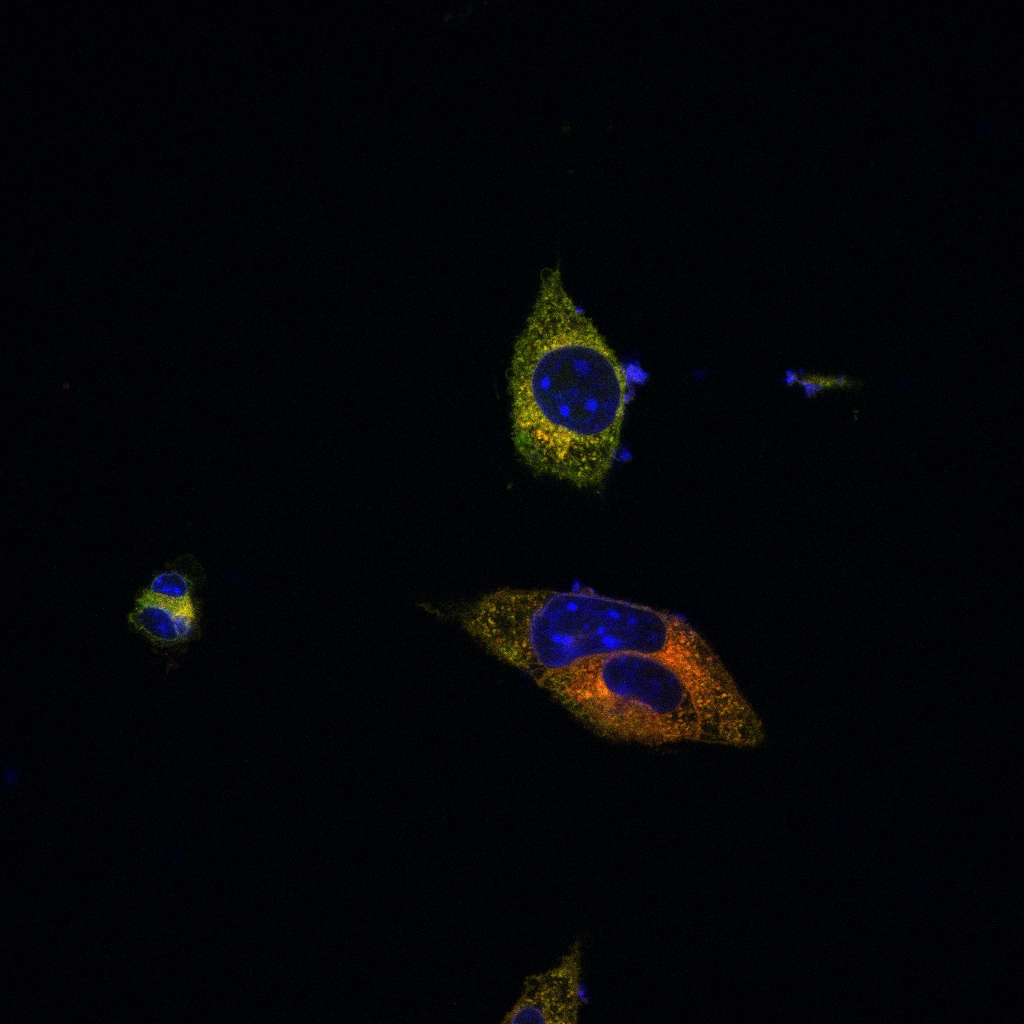

Supplement: Supplementary file 6 — Source data Fig. 2 [file 44321_2024_90_MOESM6_ESM.zip › Figure 2/2K/4T1 + erastin C11.jpg]

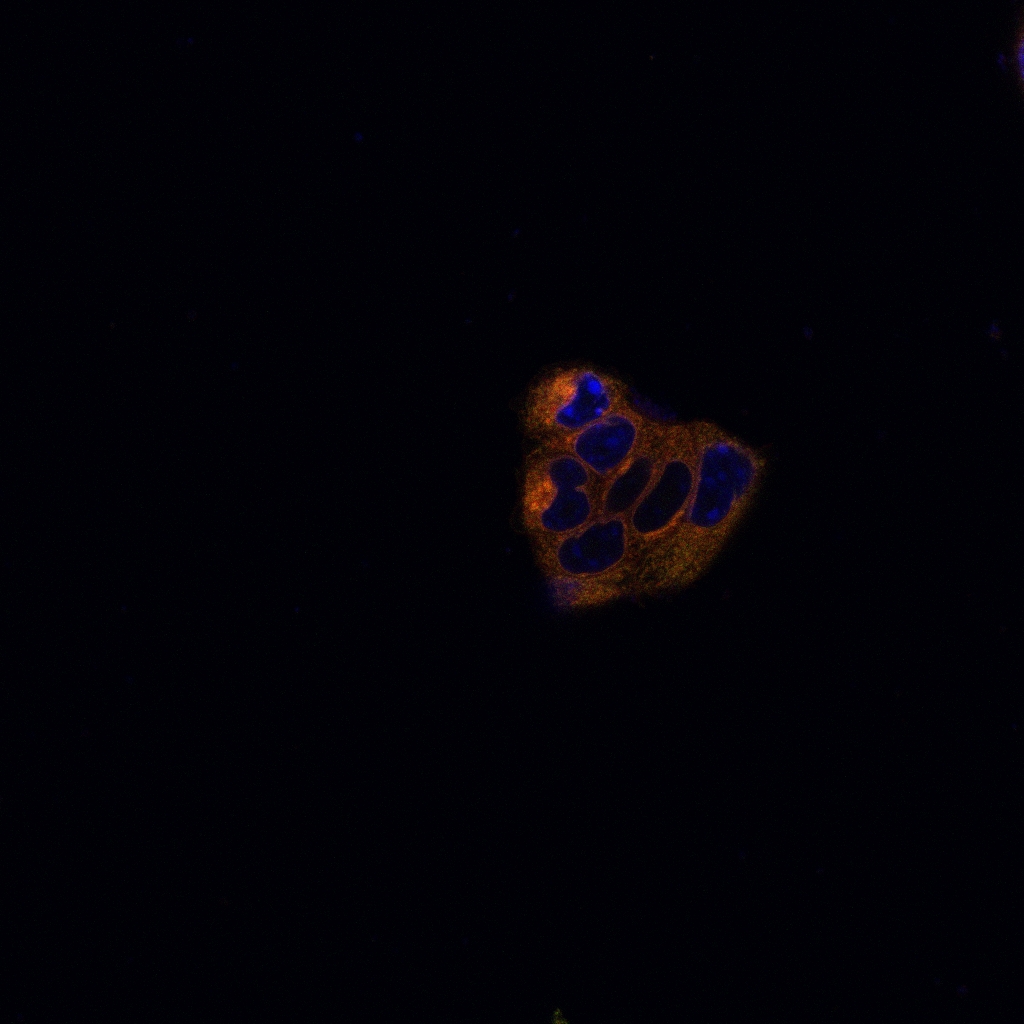

Supplement: Supplementary file 6 — Source data Fig. 2 [file 44321_2024_90_MOESM6_ESM.zip › Figure 2/2K/4T1 NT.jpg]

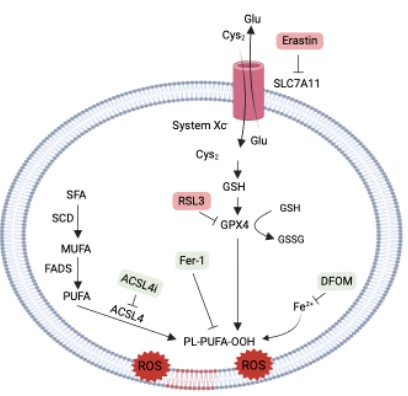

Supplement: Supplementary file 7 — Source data Fig. 3 [file 44321_2024_90_MOESM7_ESM.zip › Figure 3/3A.jpg]

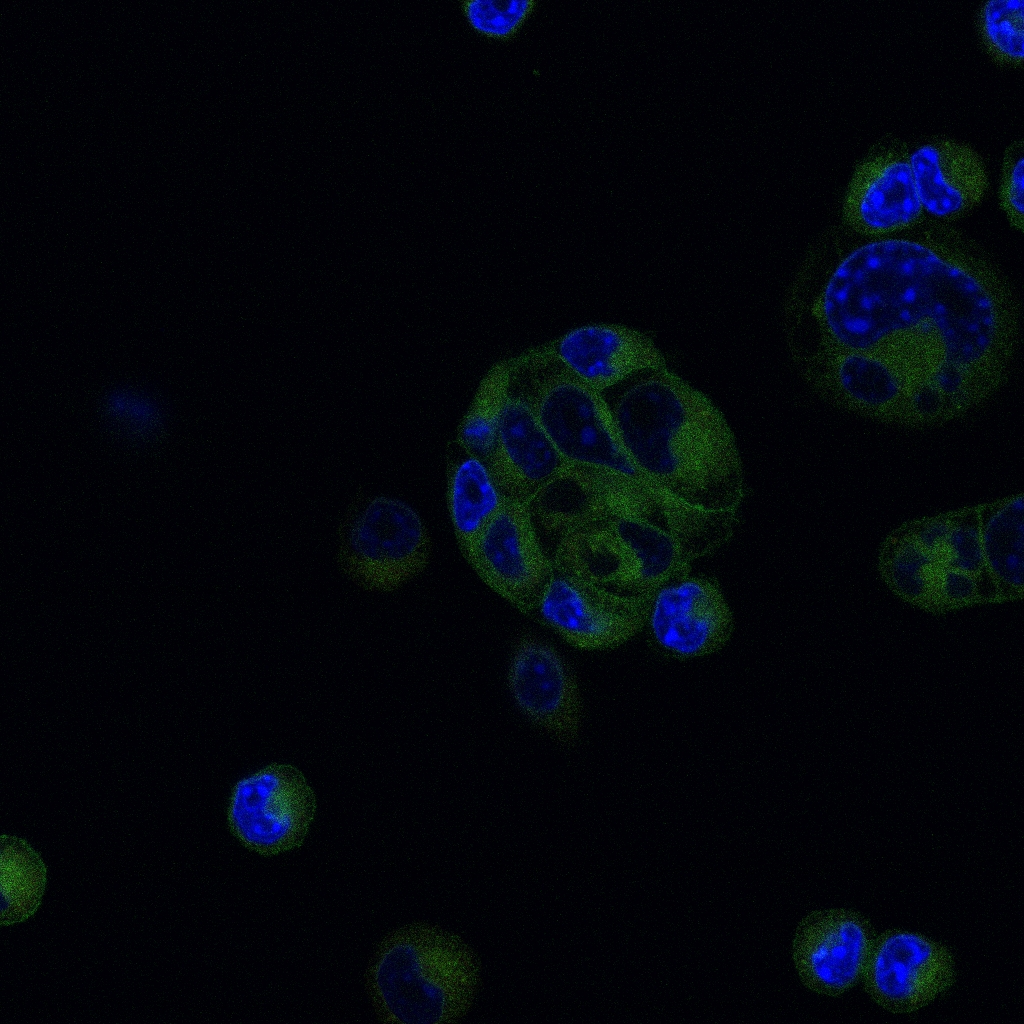

Supplement: Supplementary file 8 — Source data Fig. 4 [file 44321_2024_90_MOESM8_ESM.zip › Figure 4/4D/4T1 + RSL3 C11.jpg]

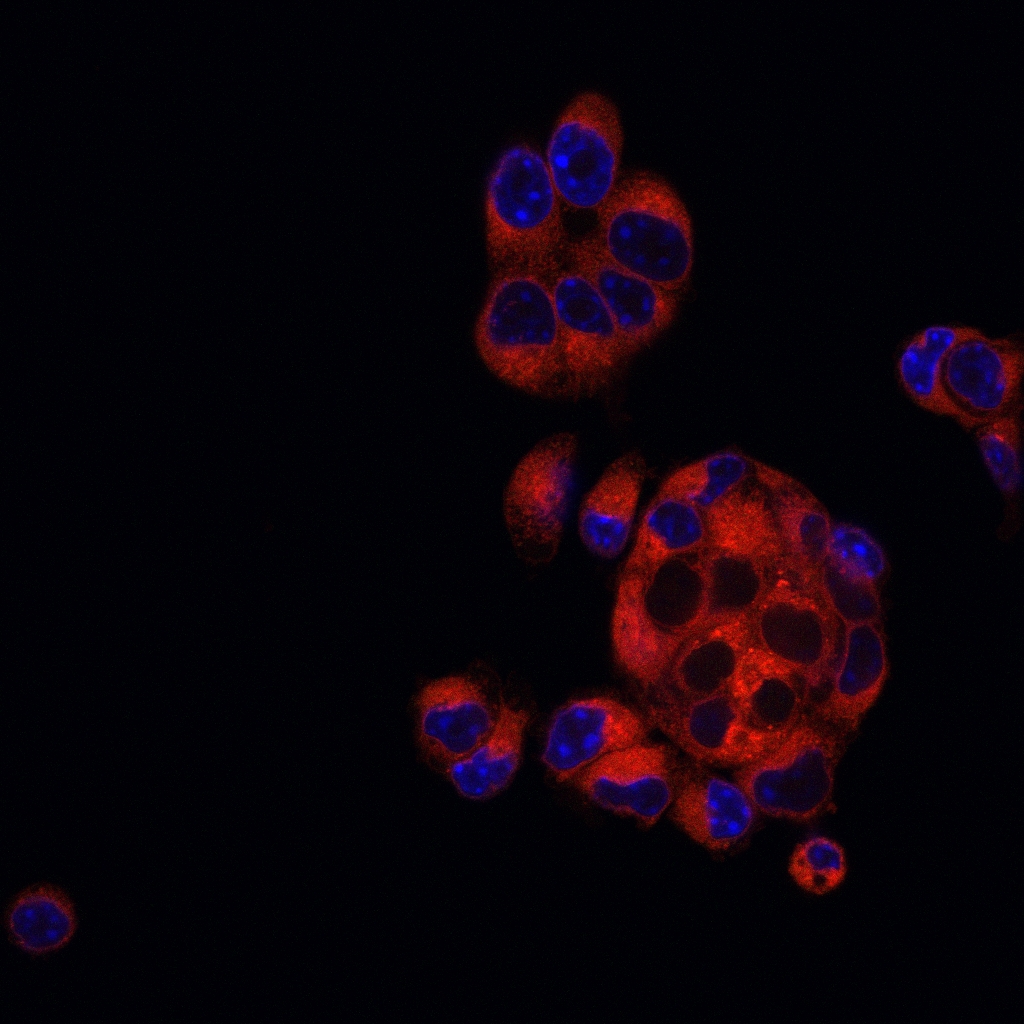

Supplement: Supplementary file 8 — Source data Fig. 4 [file 44321_2024_90_MOESM8_ESM.zip › Figure 4/4D/4T1 + SC-26196 C11.jpg]

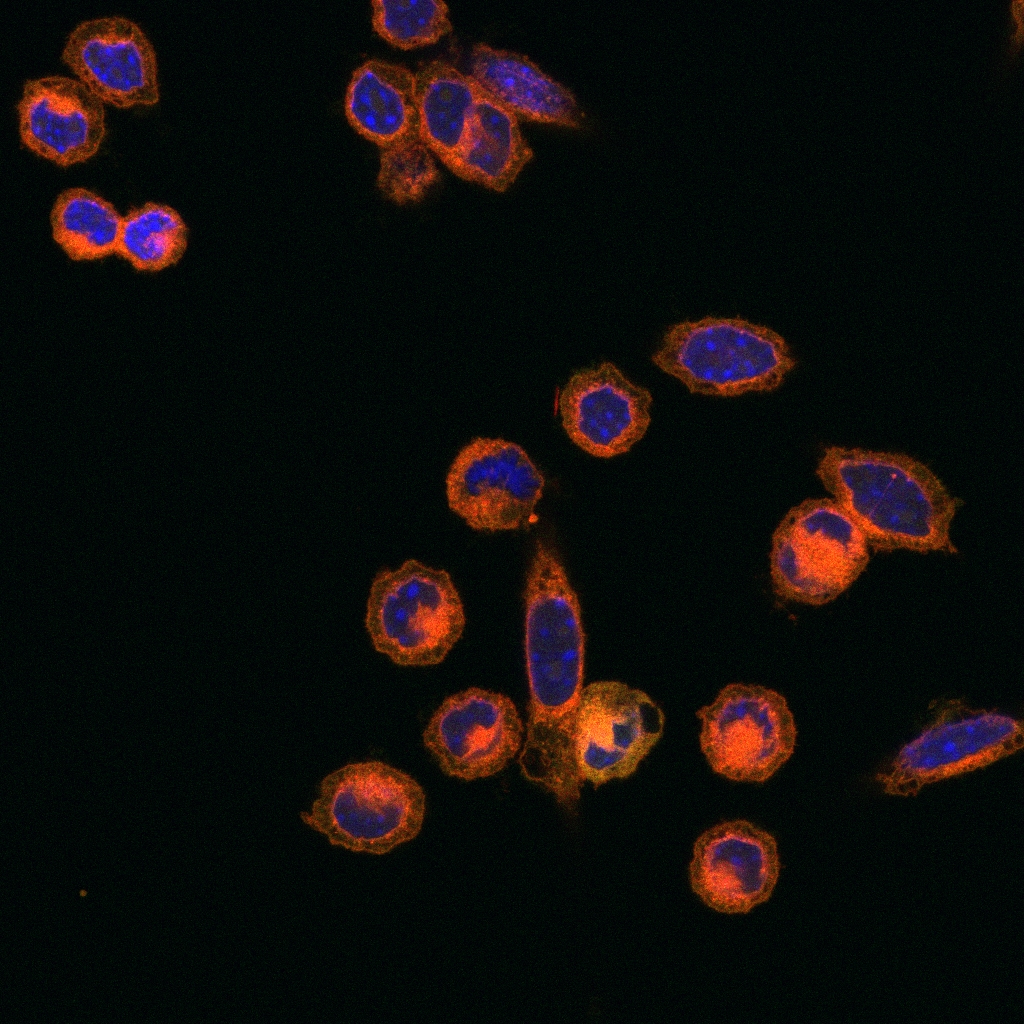

Supplement: Supplementary file 8 — Source data Fig. 4 [file 44321_2024_90_MOESM8_ESM.zip › Figure 4/4D/4T07 + SC-26196 C11.jpg]

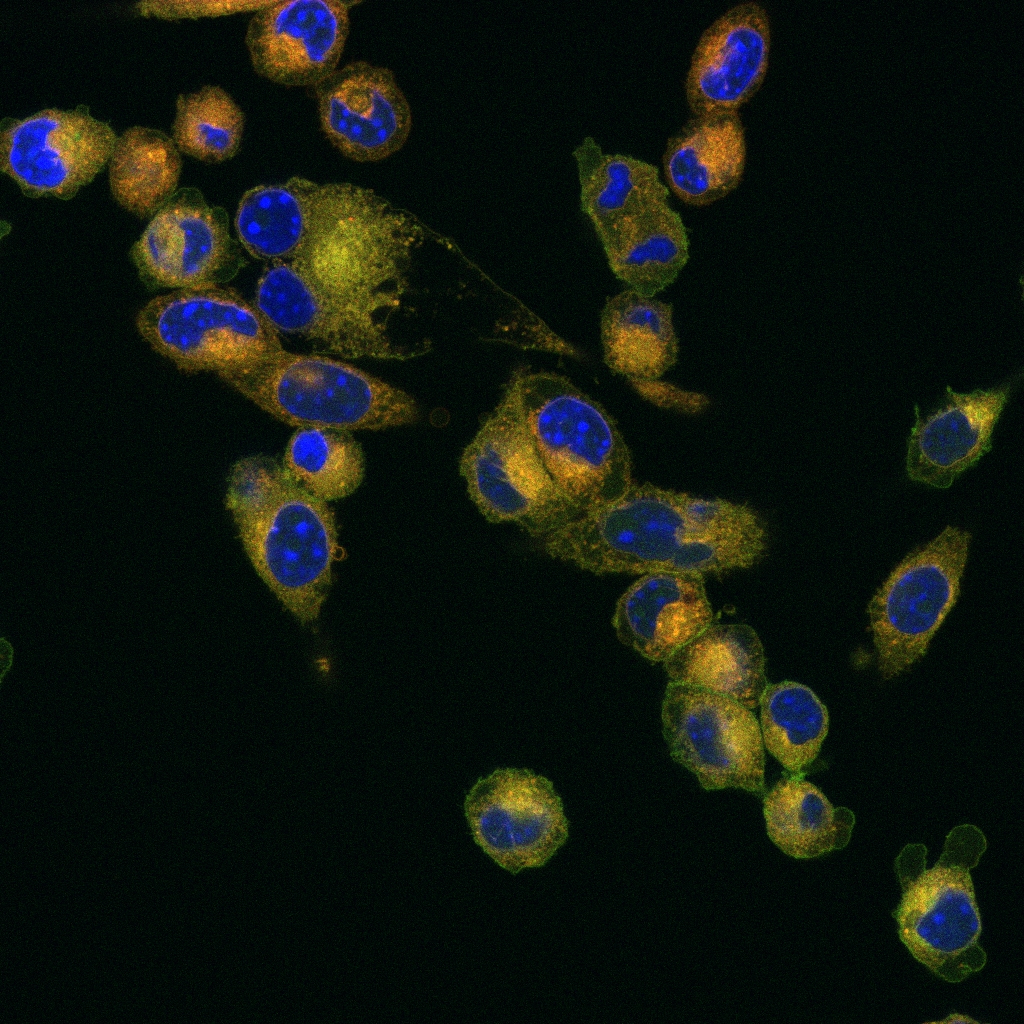

Supplement: Supplementary file 8 — Source data Fig. 4 [file 44321_2024_90_MOESM8_ESM.zip › Figure 4/4D/4T07 + RSL3 C11.jpg]

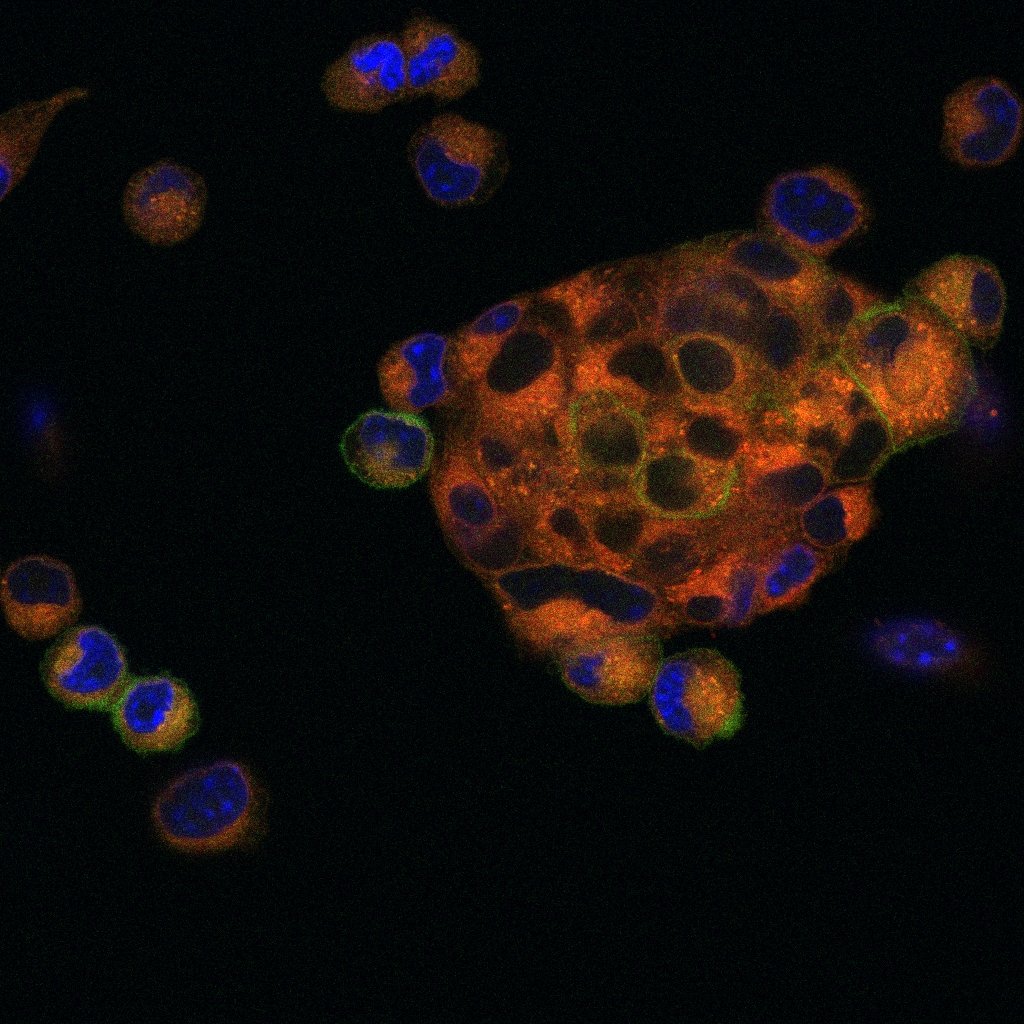

Supplement: Supplementary file 8 — Source data Fig. 4 [file 44321_2024_90_MOESM8_ESM.zip › Figure 4/4D/4T1 + CP-24879 + RSL3 C11.jpg]

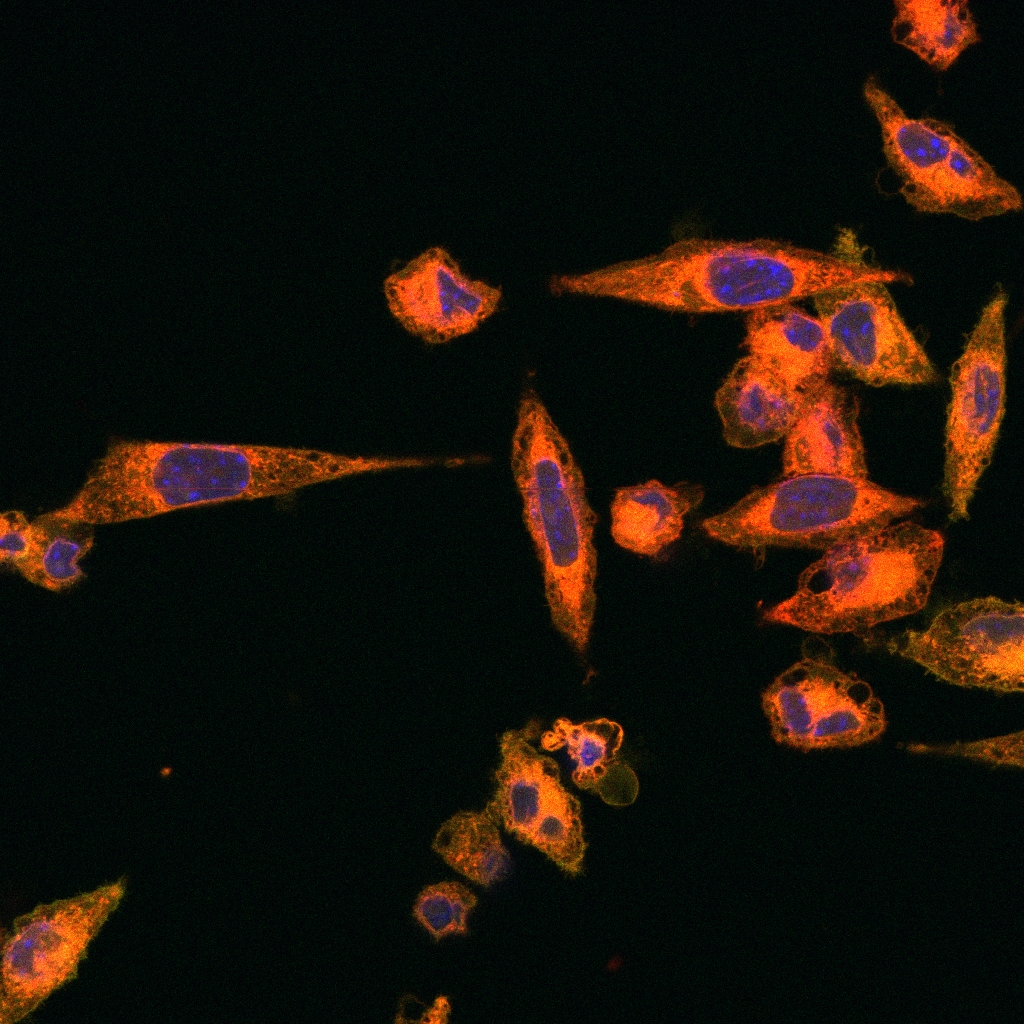

Supplement: Supplementary file 8 — Source data Fig. 4 [file 44321_2024_90_MOESM8_ESM.zip › Figure 4/4D/4T07 + SC-26196 + RSL3 C11.jpg]

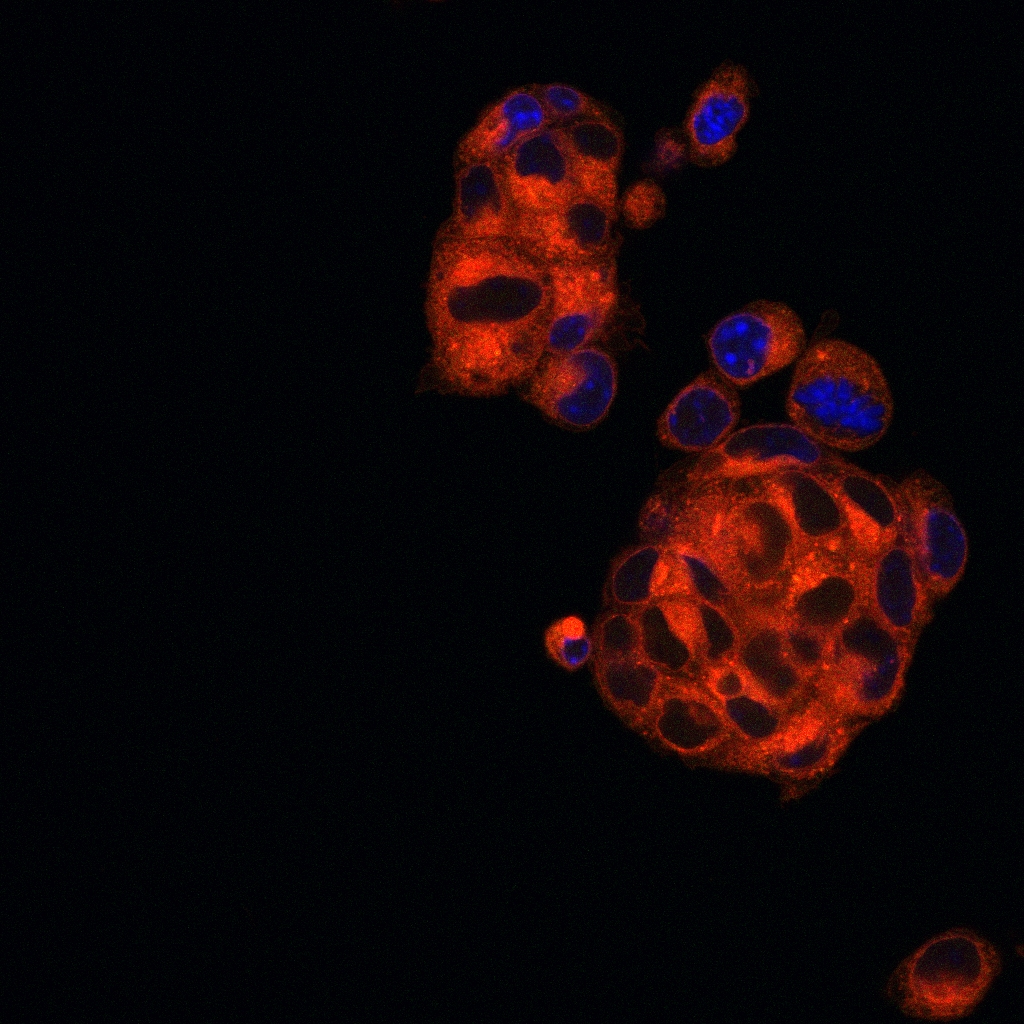

Supplement: Supplementary file 8 — Source data Fig. 4 [file 44321_2024_90_MOESM8_ESM.zip › Figure 4/4D/4T1 + CP-24879 C11.jpg]

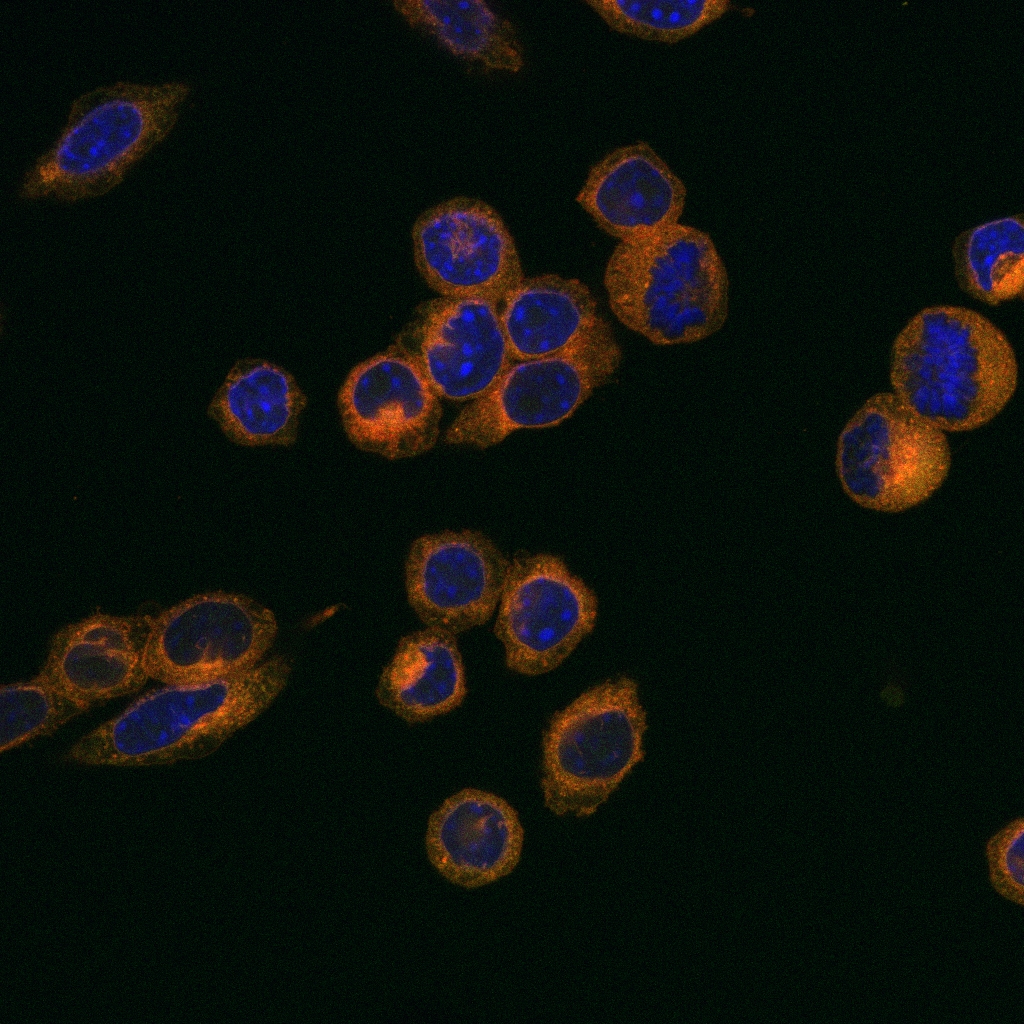

Supplement: Supplementary file 8 — Source data Fig. 4 [file 44321_2024_90_MOESM8_ESM.zip › Figure 4/4D/4T07 + CP-24879 C11.jpg]

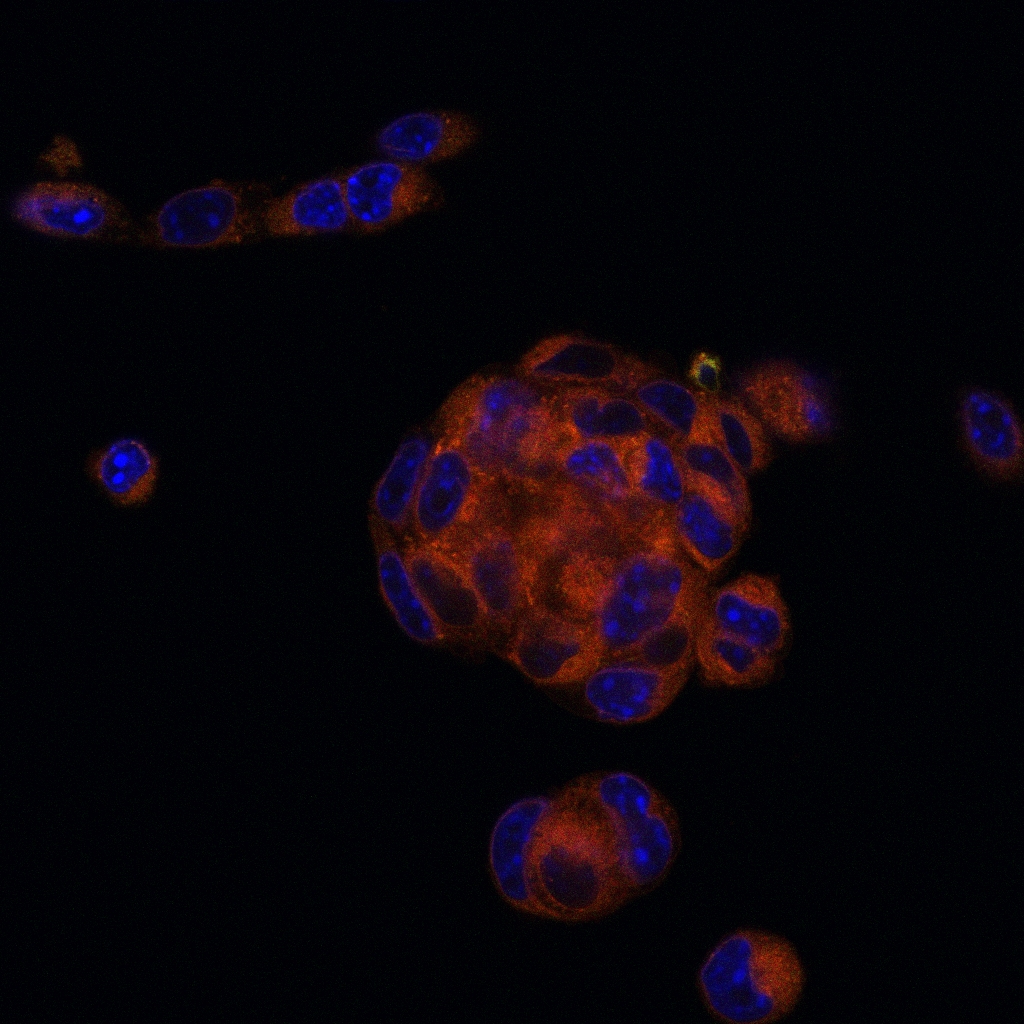

Supplement: Supplementary file 8 — Source data Fig. 4 [file 44321_2024_90_MOESM8_ESM.zip › Figure 4/4D/4T1 NT C11.jpg]

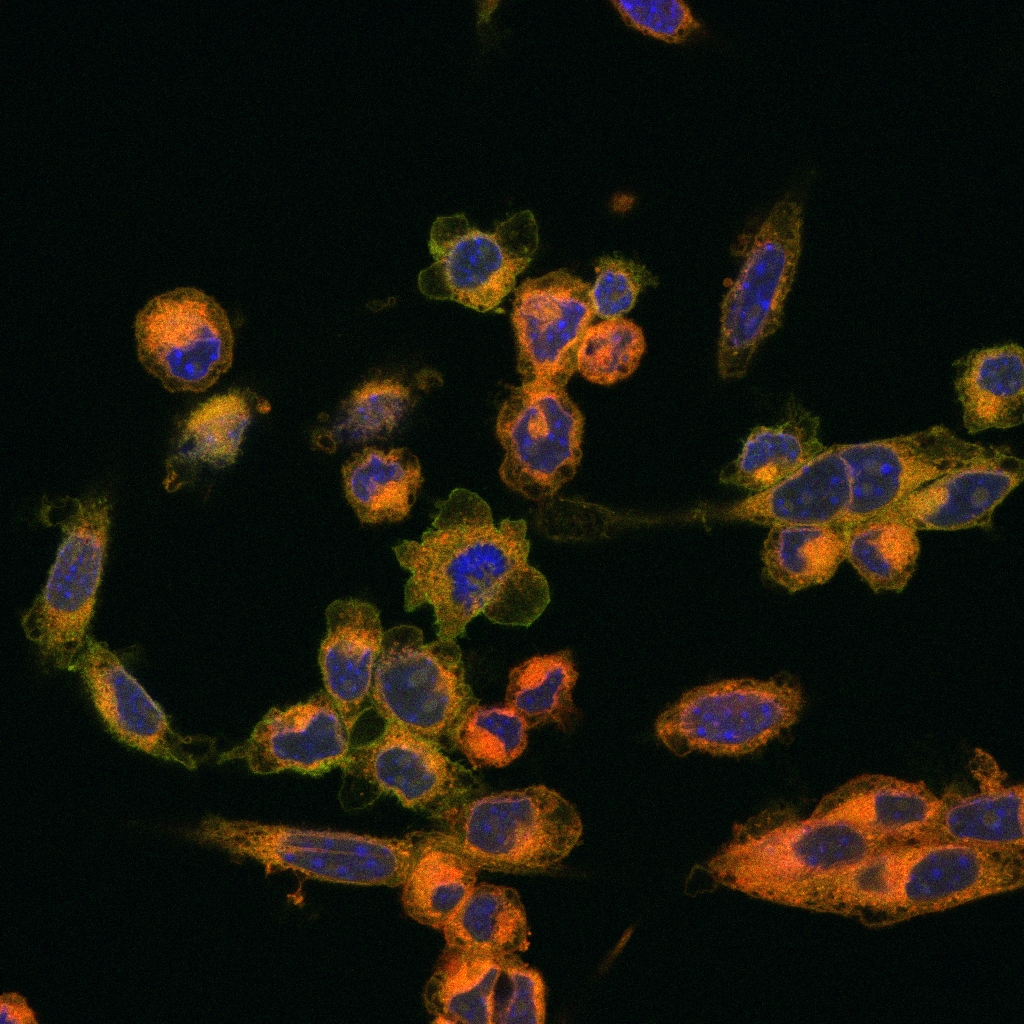

Supplement: Supplementary file 8 — Source data Fig. 4 [file 44321_2024_90_MOESM8_ESM.zip › Figure 4/4D/4T07 + CP-24879 + RSL3 C11.jpg]

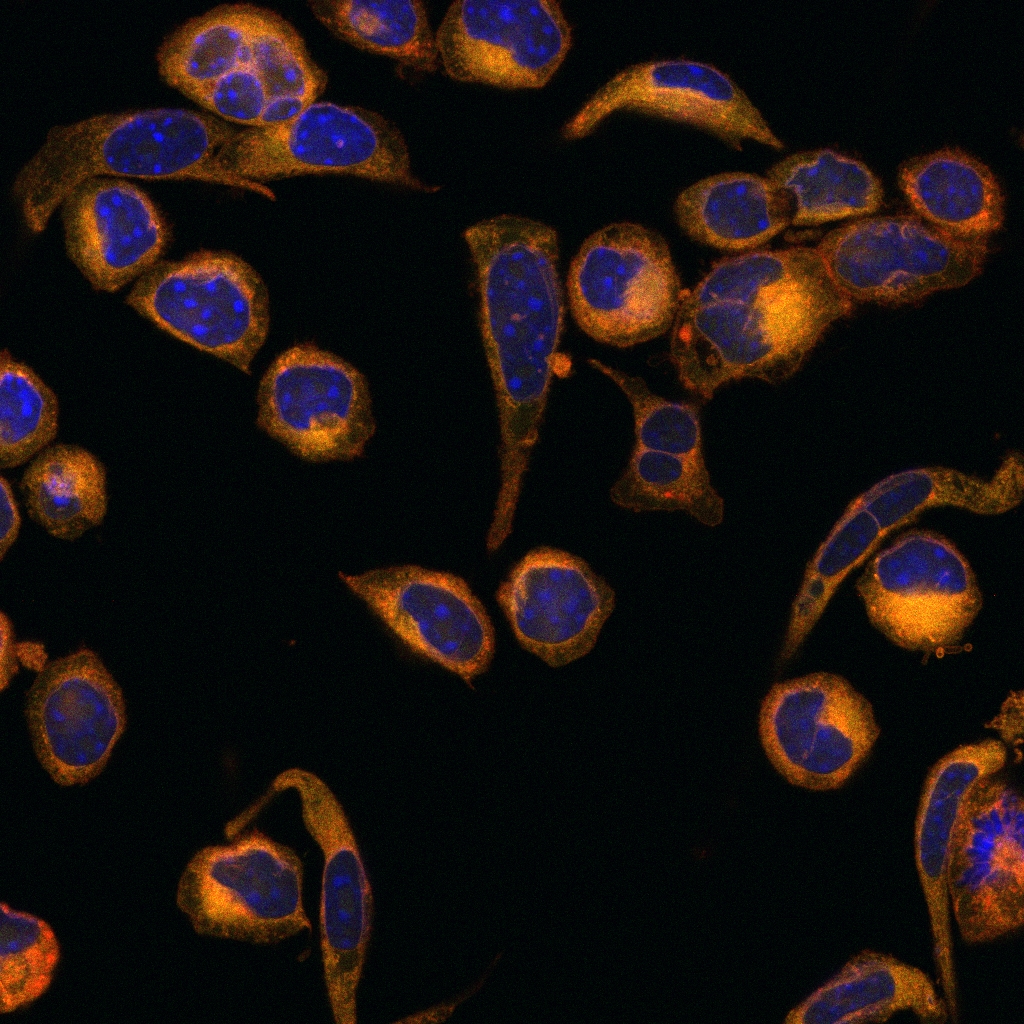

Supplement: Supplementary file 8 — Source data Fig. 4 [file 44321_2024_90_MOESM8_ESM.zip › Figure 4/4D/4T07 NT C11.jpg]

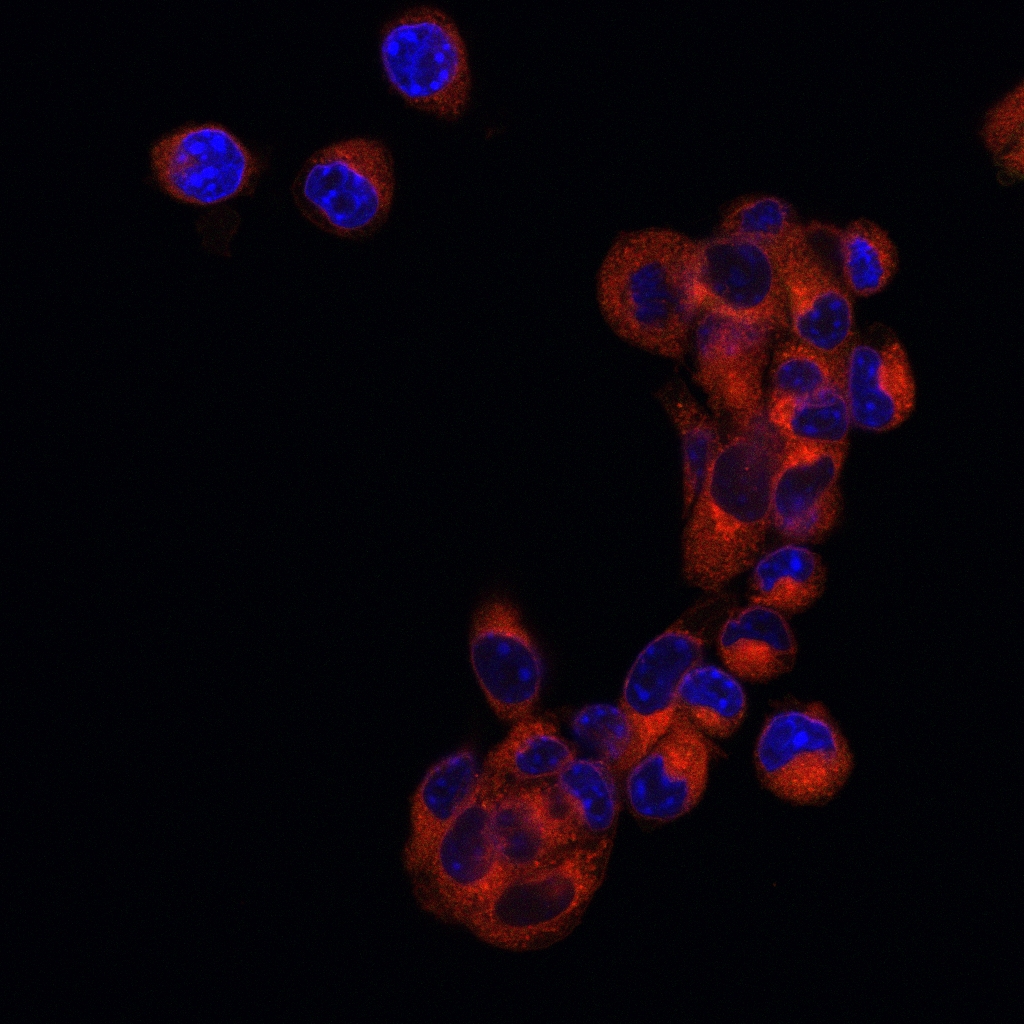

Supplement: Supplementary file 8 — Source data Fig. 4 [file 44321_2024_90_MOESM8_ESM.zip › Figure 4/4D/4T1 + SC-26196 + RSL3 C11.jpg]

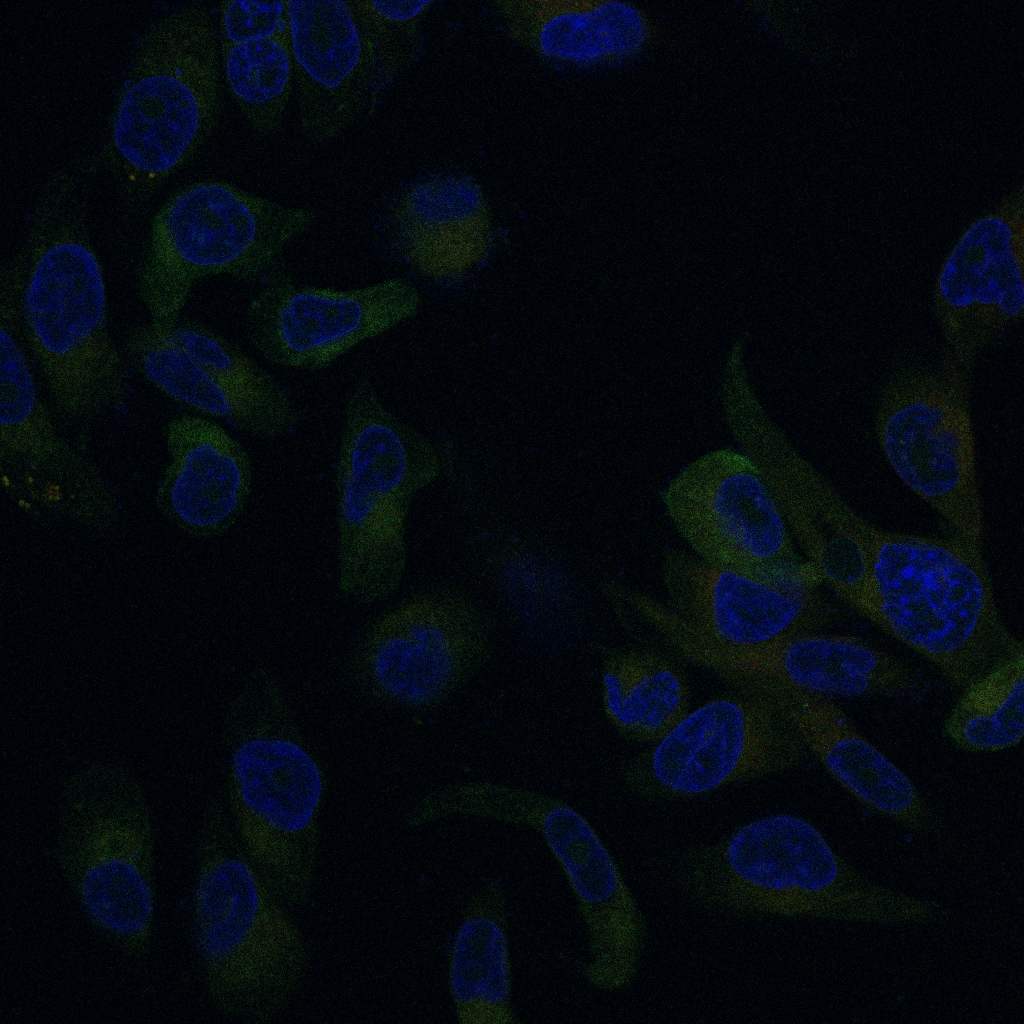

Supplement: Supplementary file 8 — Source data Fig. 4 [file 44321_2024_90_MOESM8_ESM.zip › Figure 4/4H/MDA-MB-231 NT + RSL3_C11.jpg]

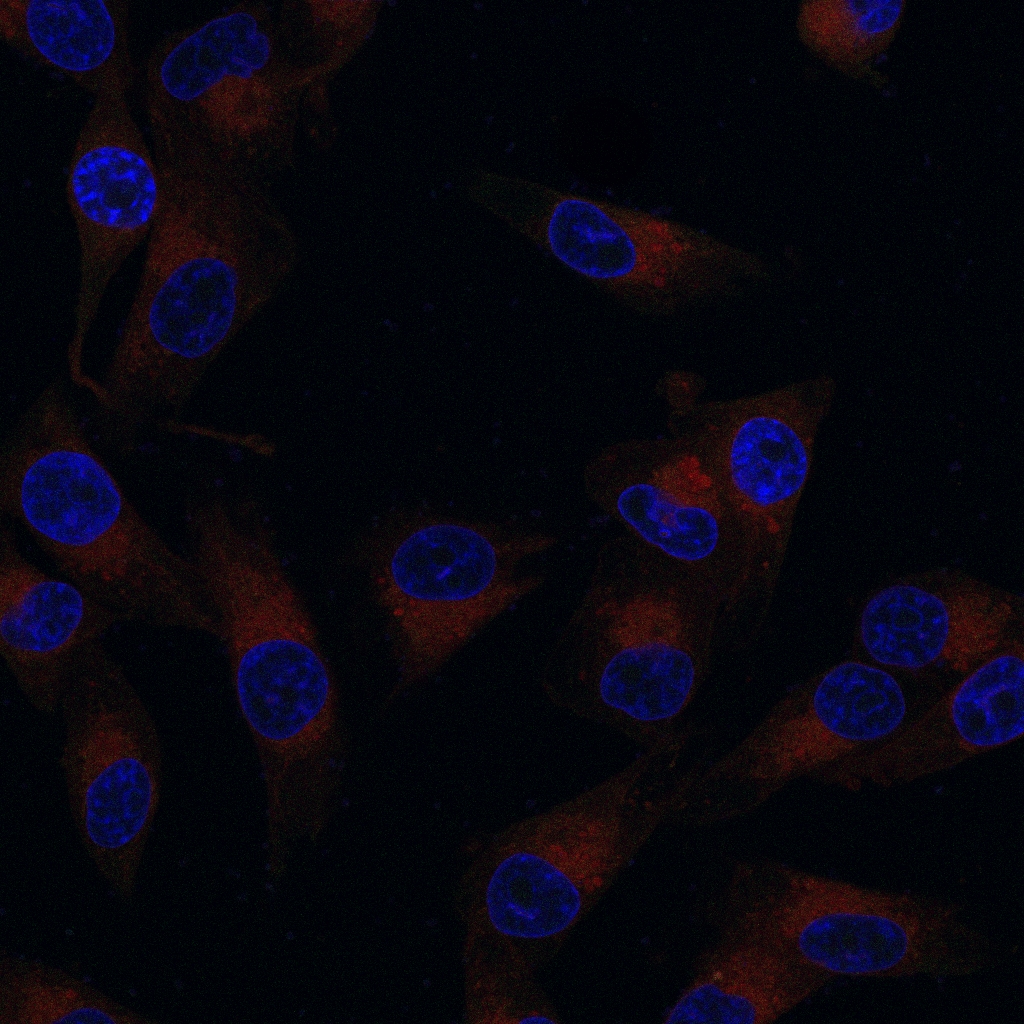

Supplement: Supplementary file 8 — Source data Fig. 4 [file 44321_2024_90_MOESM8_ESM.zip › Figure 4/4H/MDA-MB-231 SC-26196 + RSL3_C11.jpg]

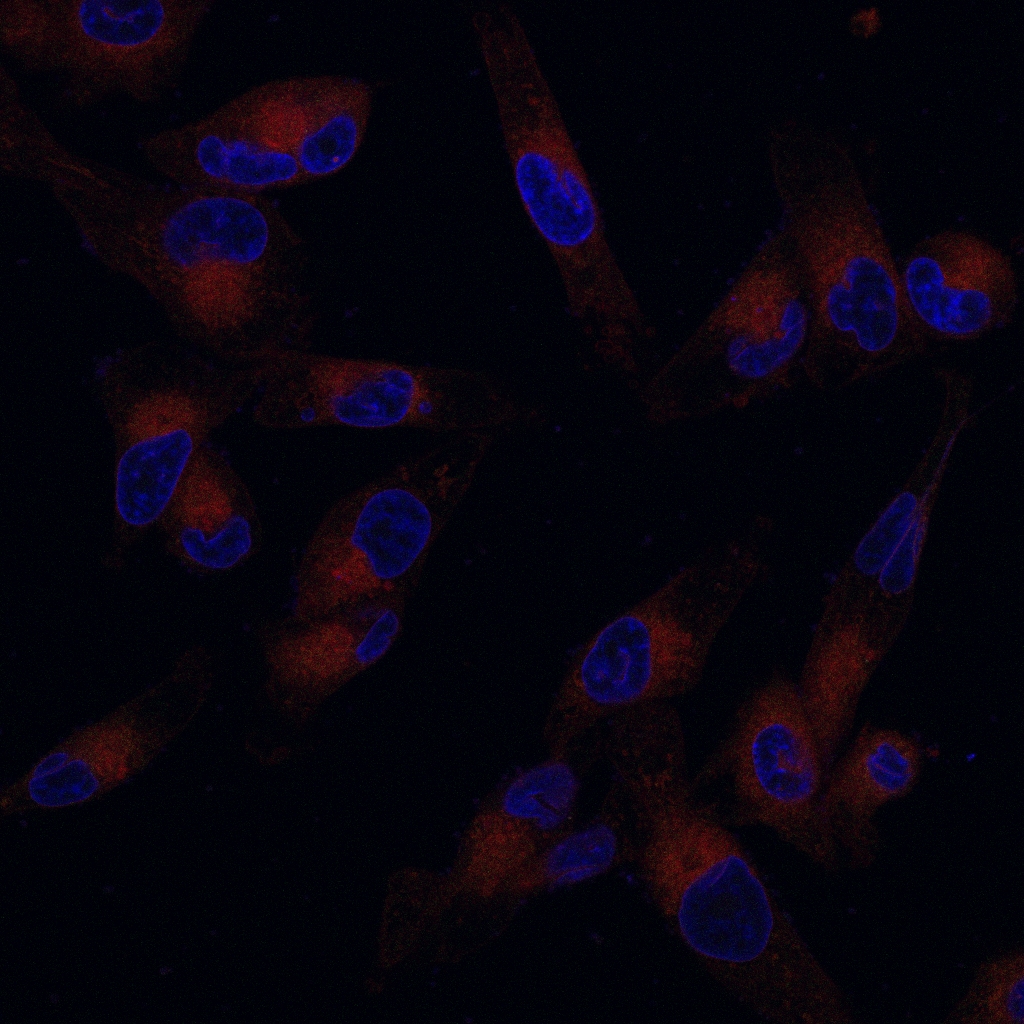

Supplement: Supplementary file 8 — Source data Fig. 4 [file 44321_2024_90_MOESM8_ESM.zip › Figure 4/4H/MDA-MB-231 CP-24879_C11.jpg]

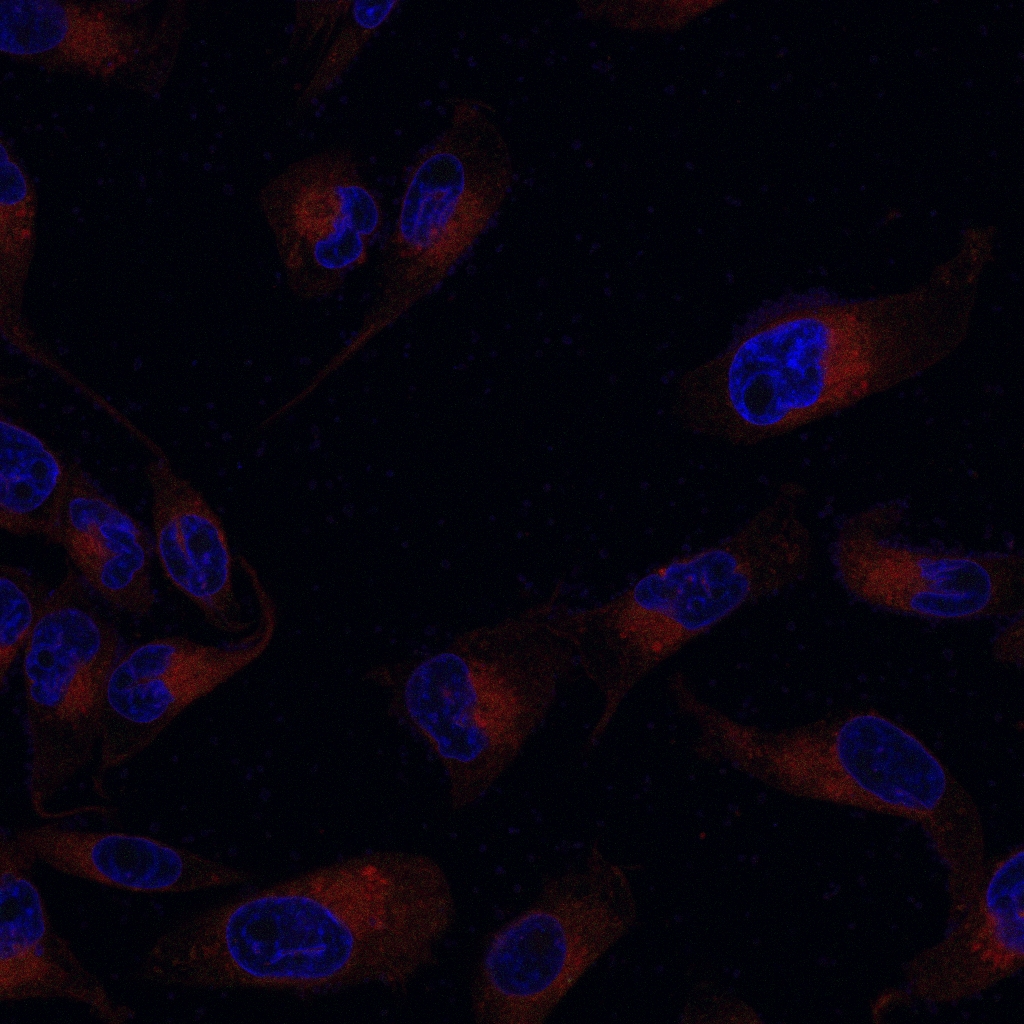

Supplement: Supplementary file 8 — Source data Fig. 4 [file 44321_2024_90_MOESM8_ESM.zip › Figure 4/4H/MDA-MB-231 SC-26196_C11.jpg]

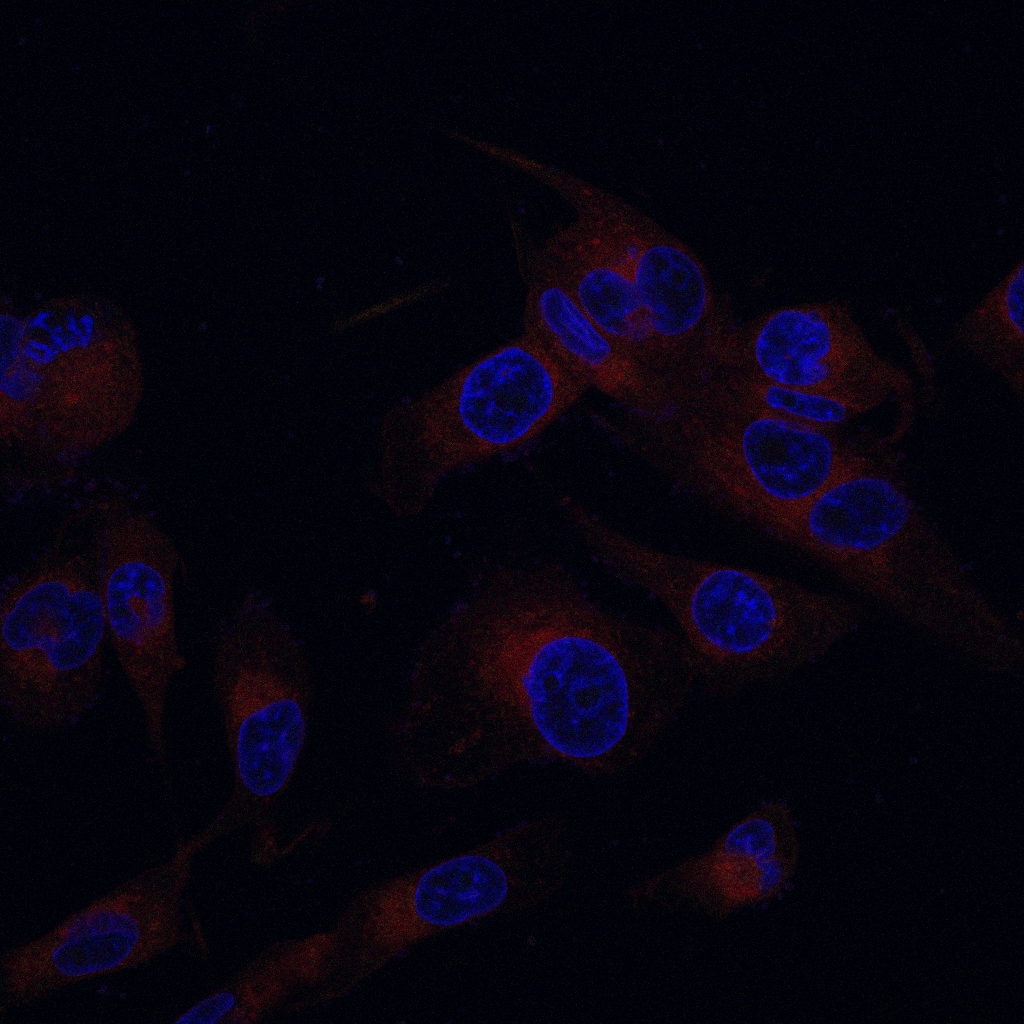

Supplement: Supplementary file 8 — Source data Fig. 4 [file 44321_2024_90_MOESM8_ESM.zip › Figure 4/4H/MDA-MB-231 NT_C11.jpg]

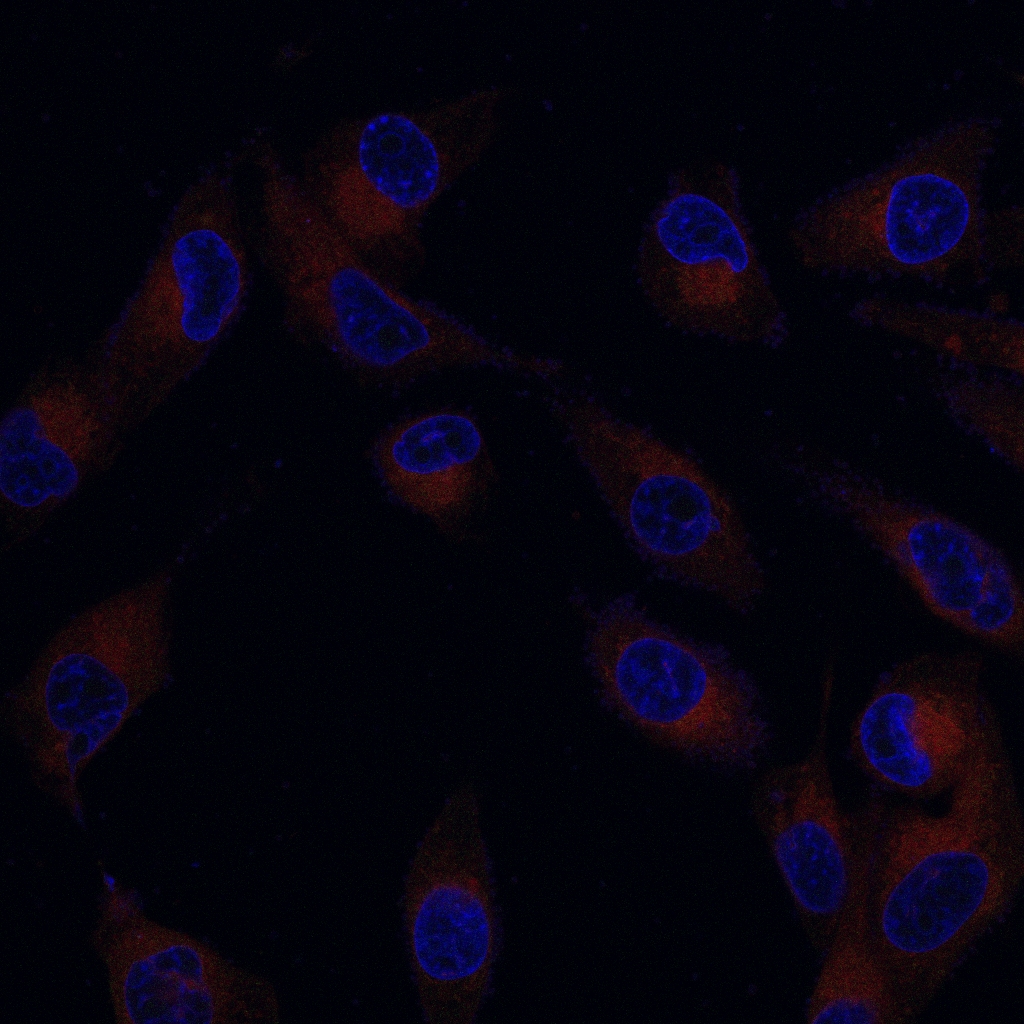

Supplement: Supplementary file 8 — Source data Fig. 4 [file 44321_2024_90_MOESM8_ESM.zip › Figure 4/4H/MDA-MB-231 CP-24879 + RSL3_C11.jpg]

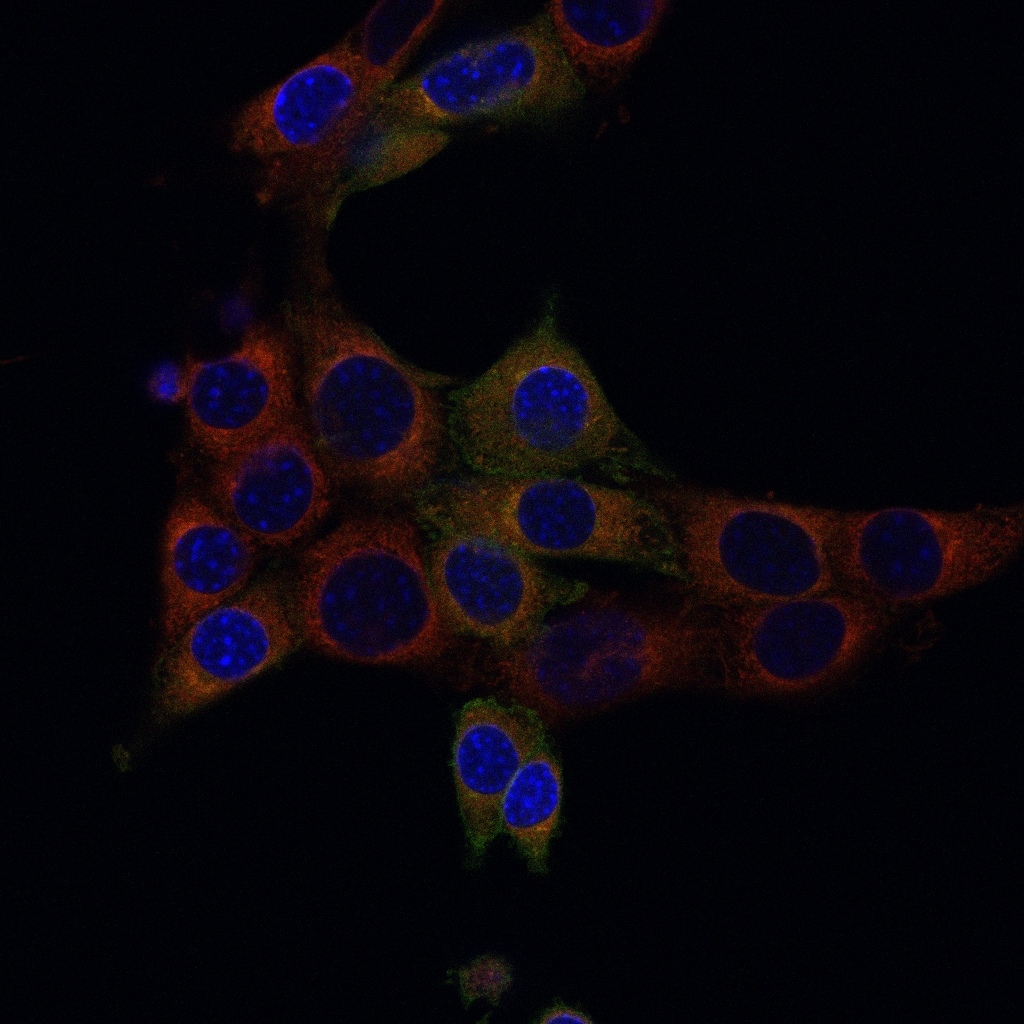

Supplement: Supplementary file 10 — Source data Fig. 6 [file 44321_2024_90_MOESM10_ESM.zip › Figure 6/6B/67NR + RSL3_C11.jpg]

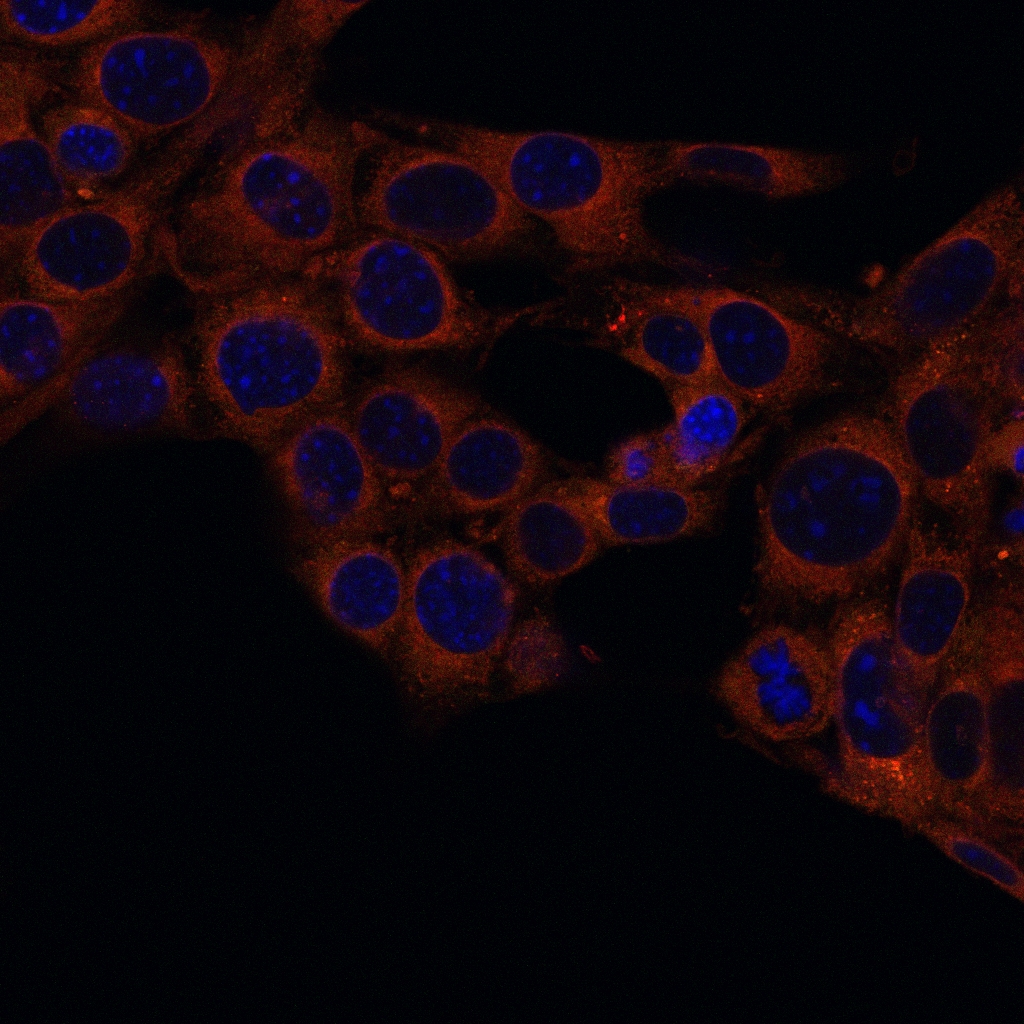

Supplement: Supplementary file 10 — Source data Fig. 6 [file 44321_2024_90_MOESM10_ESM.zip › Figure 6/6B/67NR_C11.jpg]

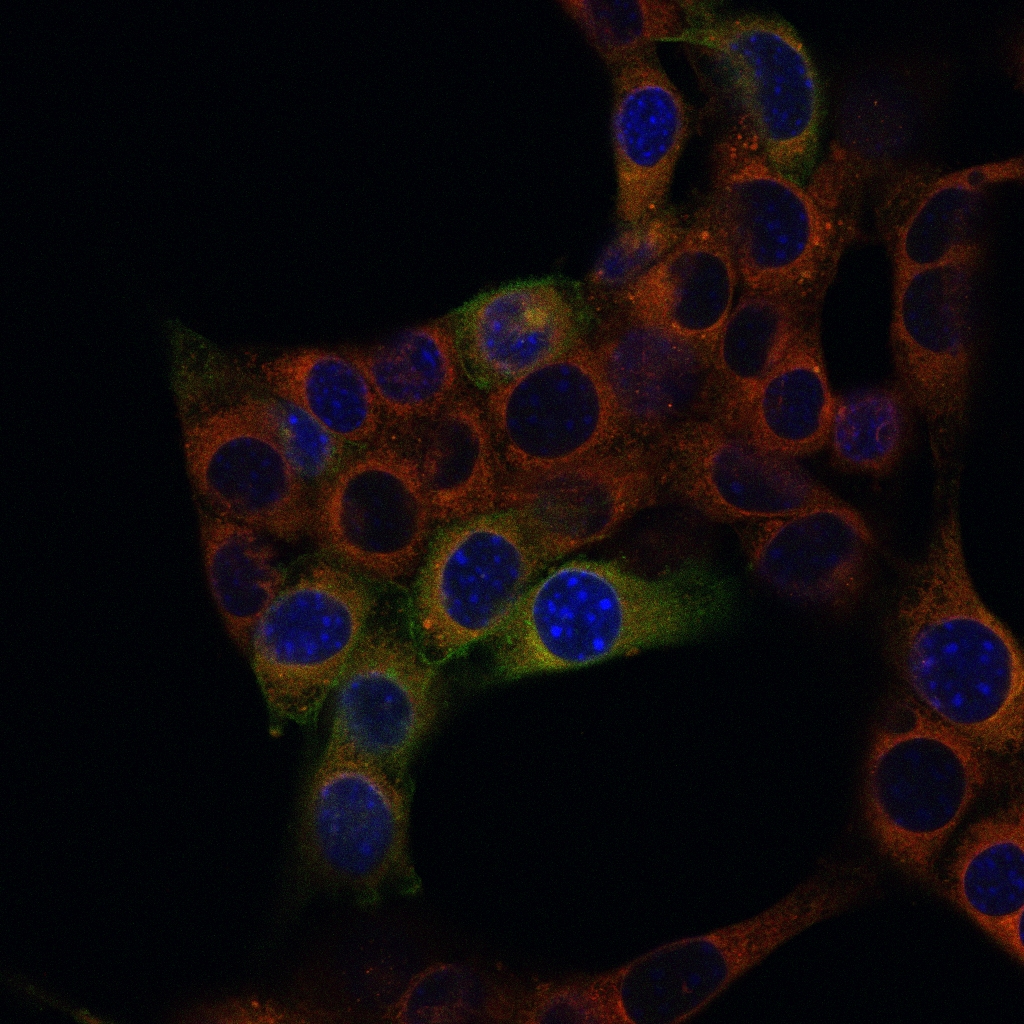

Supplement: Supplementary file 10 — Source data Fig. 6 [file 44321_2024_90_MOESM10_ESM.zip › Figure 6/6B/67NR + 22.4 + RSL3_C11.jpg]

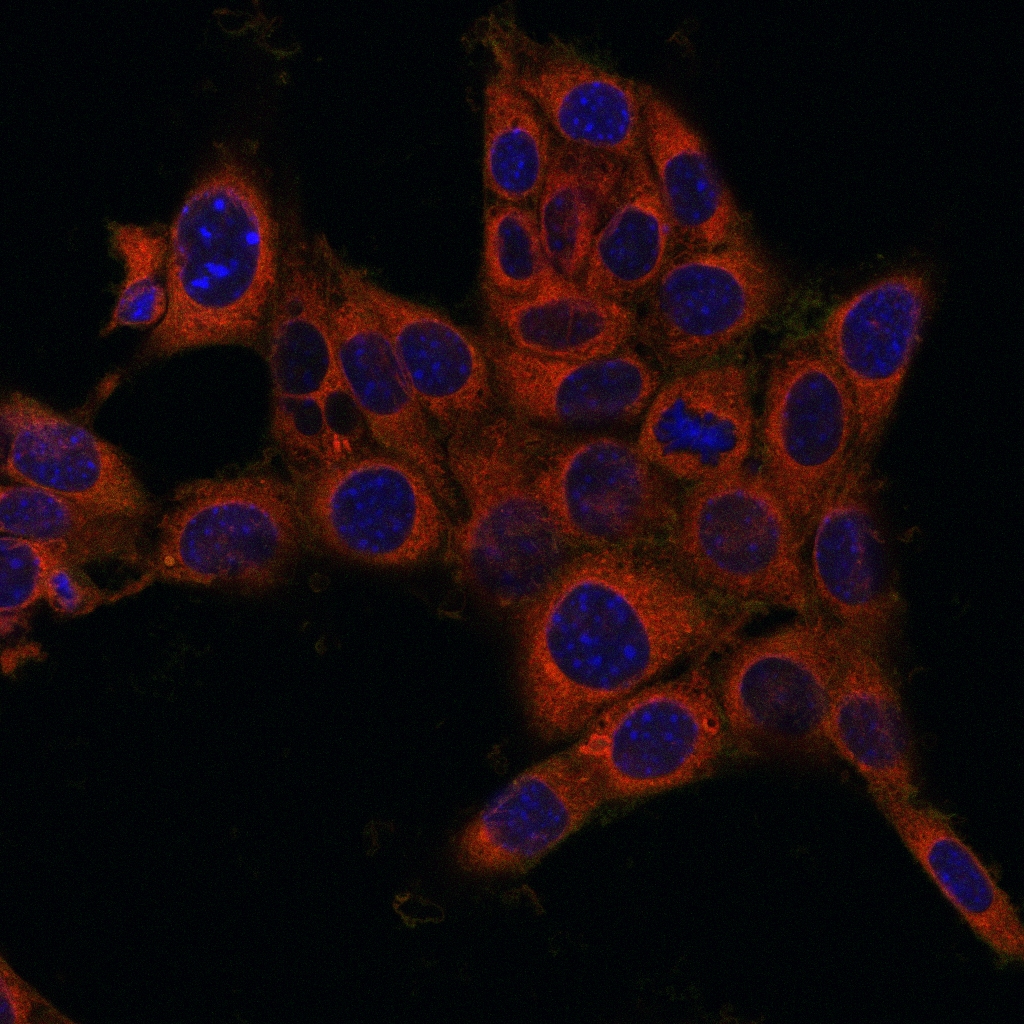

Supplement: Supplementary file 10 — Source data Fig. 6 [file 44321_2024_90_MOESM10_ESM.zip › Figure 6/6B/67NR + 22.4.jpg]

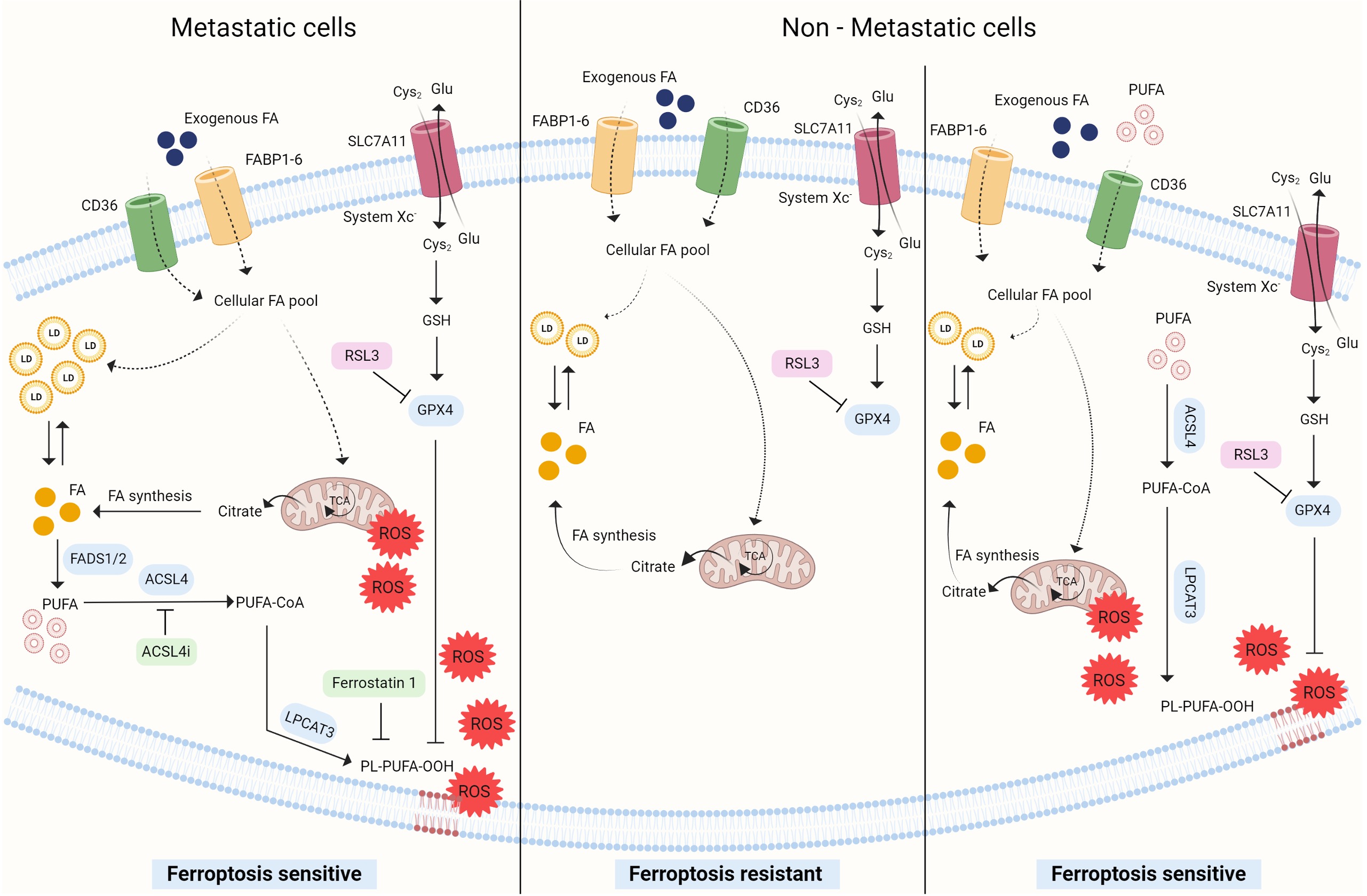

Supplement: Supplementary file 11 — Source data Fig. 7 [file 44321_2024_90_MOESM11_ESM.zip › Figure 7/Graphical abstract.jpg]

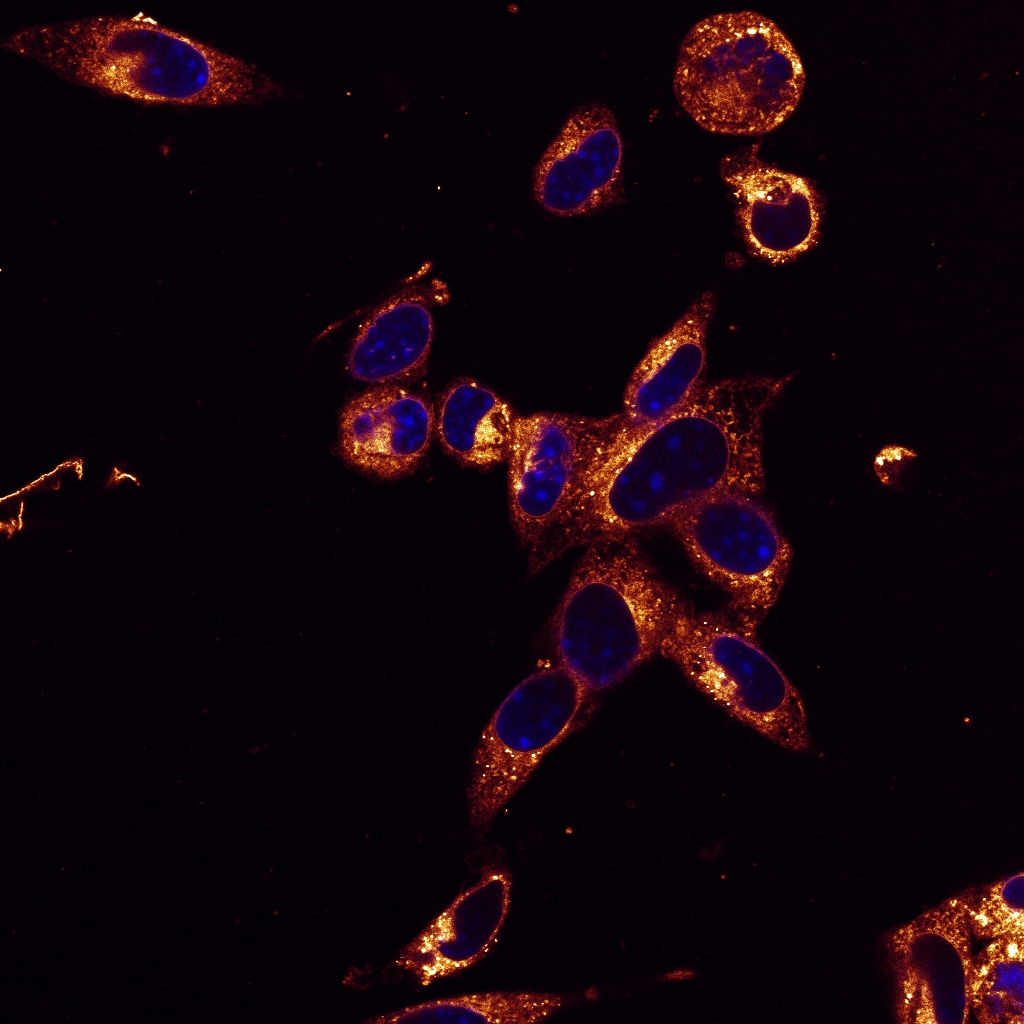

Supplement: Supplementary file 11 — Source data Fig. 7 [file 44321_2024_90_MOESM11_ESM.zip › Figure 7/7B/4T07 + RSL3 LDs.jpg]

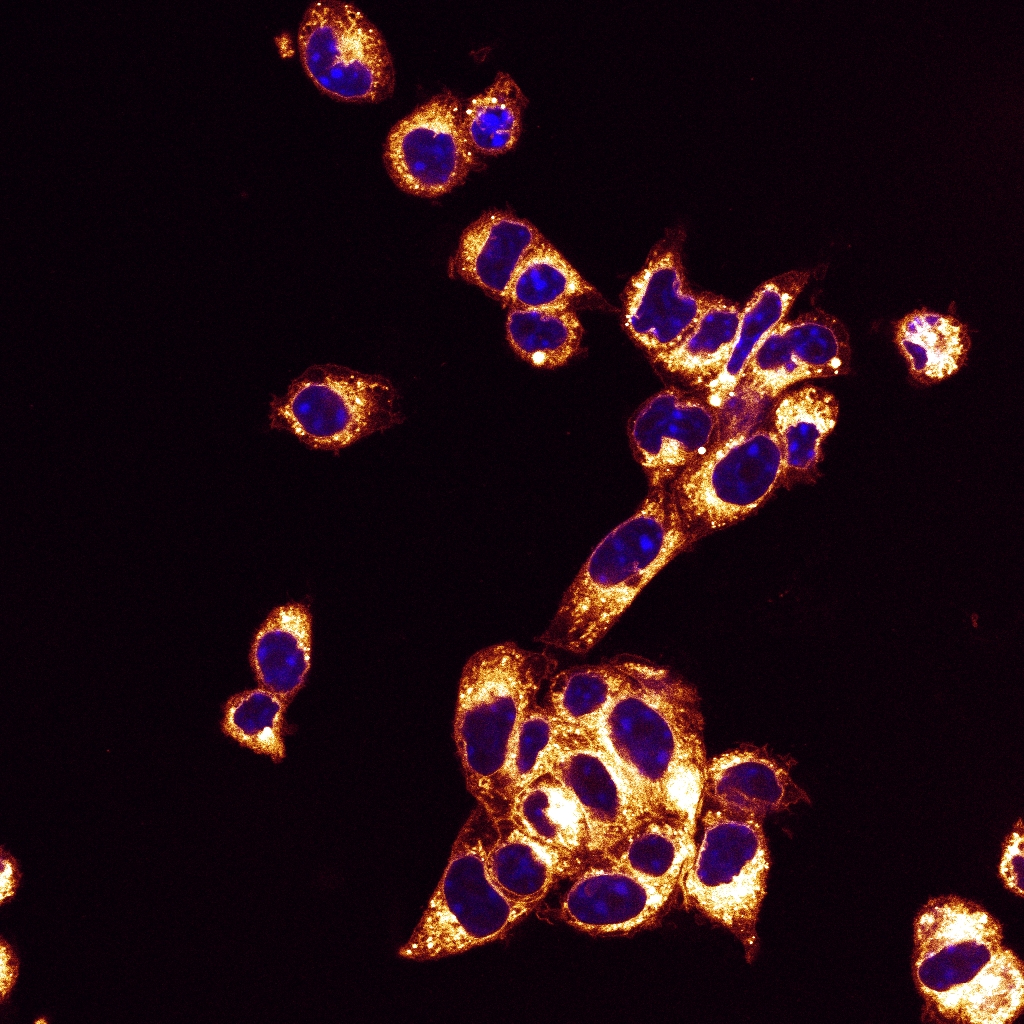

Supplement: Supplementary file 11 — Source data Fig. 7 [file 44321_2024_90_MOESM11_ESM.zip › Figure 7/7B/4T1 + RSL3 LDs.jpg]

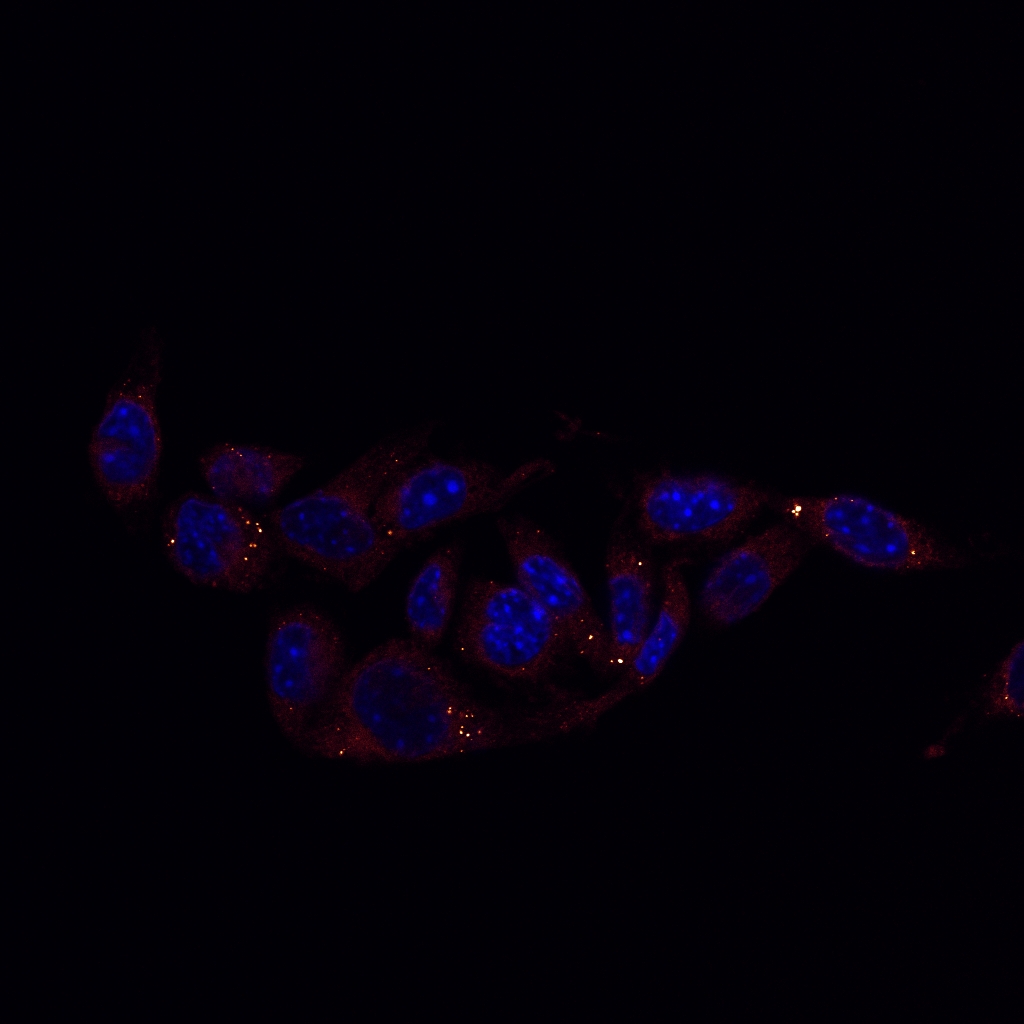

Supplement: Supplementary file 11 — Source data Fig. 7 [file 44321_2024_90_MOESM11_ESM.zip › Figure 7/7B/67NR NT LDs.jpg]

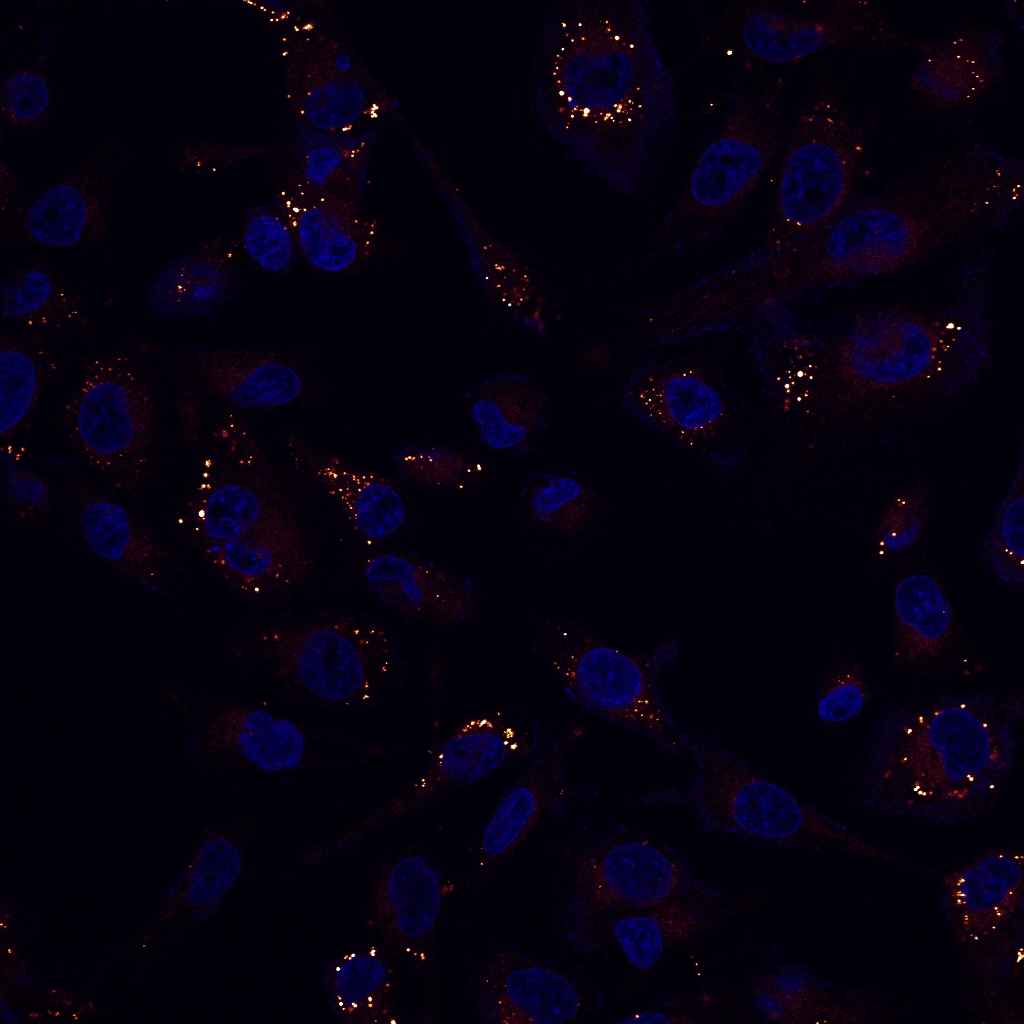

Supplement: Supplementary file 11 — Source data Fig. 7 [file 44321_2024_90_MOESM11_ESM.zip › Figure 7/7B/MDA-MB-231 NT LDs.jpg]

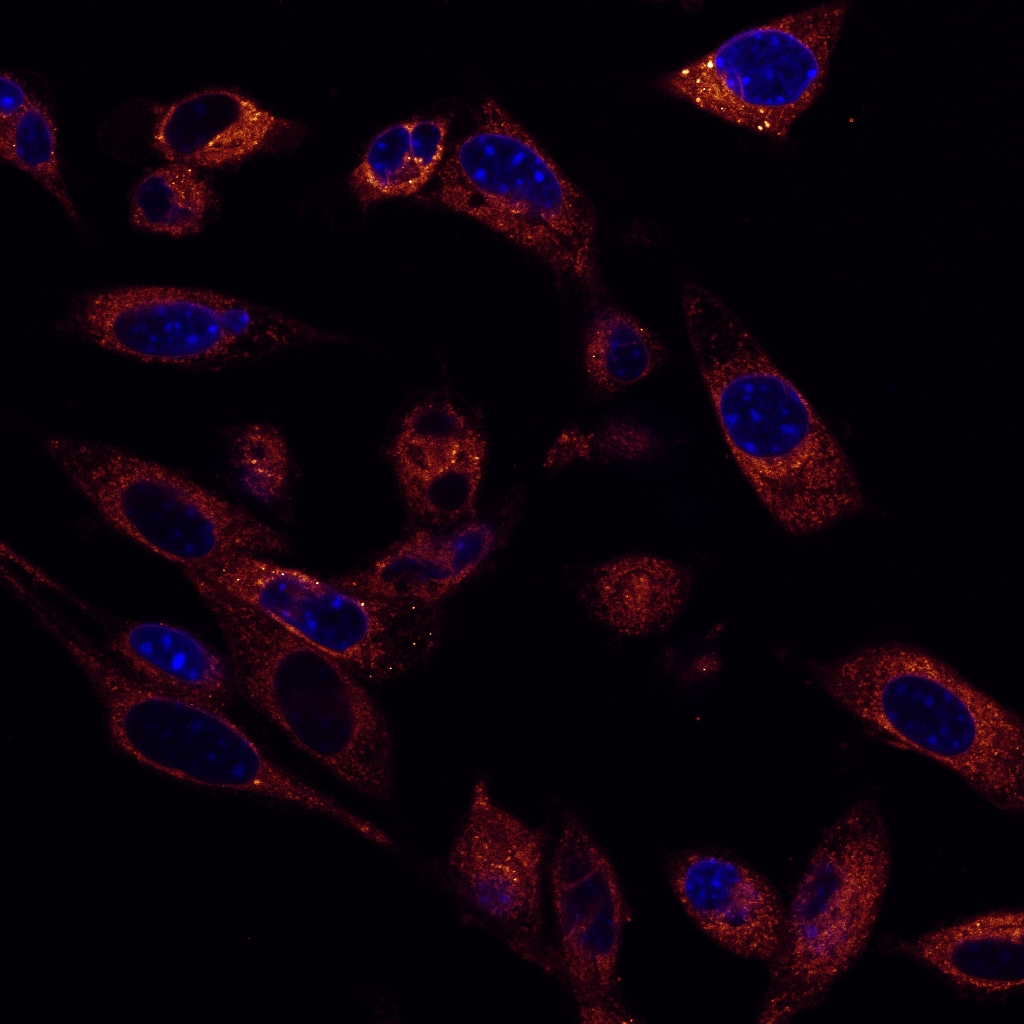

Supplement: Supplementary file 11 — Source data Fig. 7 [file 44321_2024_90_MOESM11_ESM.zip › Figure 7/7B/4T07 NT LDs.jpg]

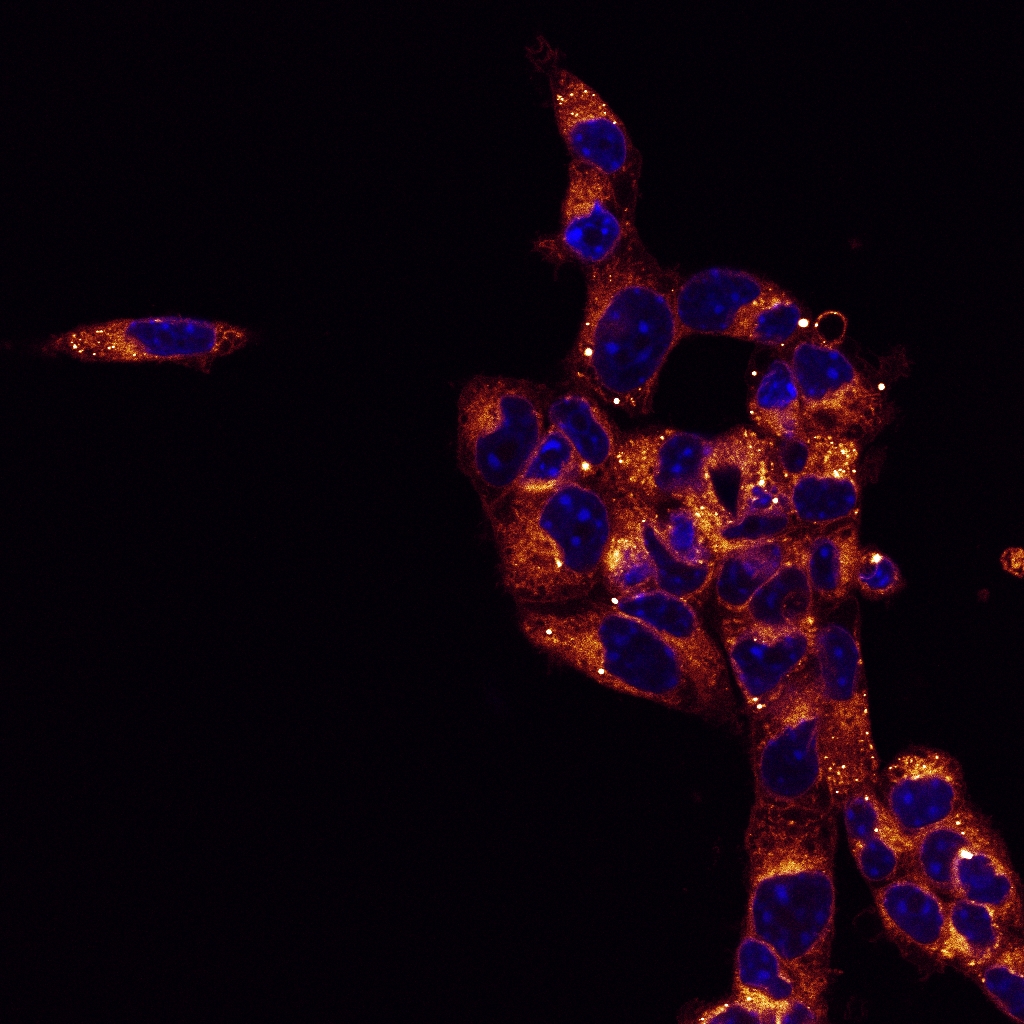

Supplement: Supplementary file 11 — Source data Fig. 7 [file 44321_2024_90_MOESM11_ESM.zip › Figure 7/7B/4T1 NT LDs.jpg]

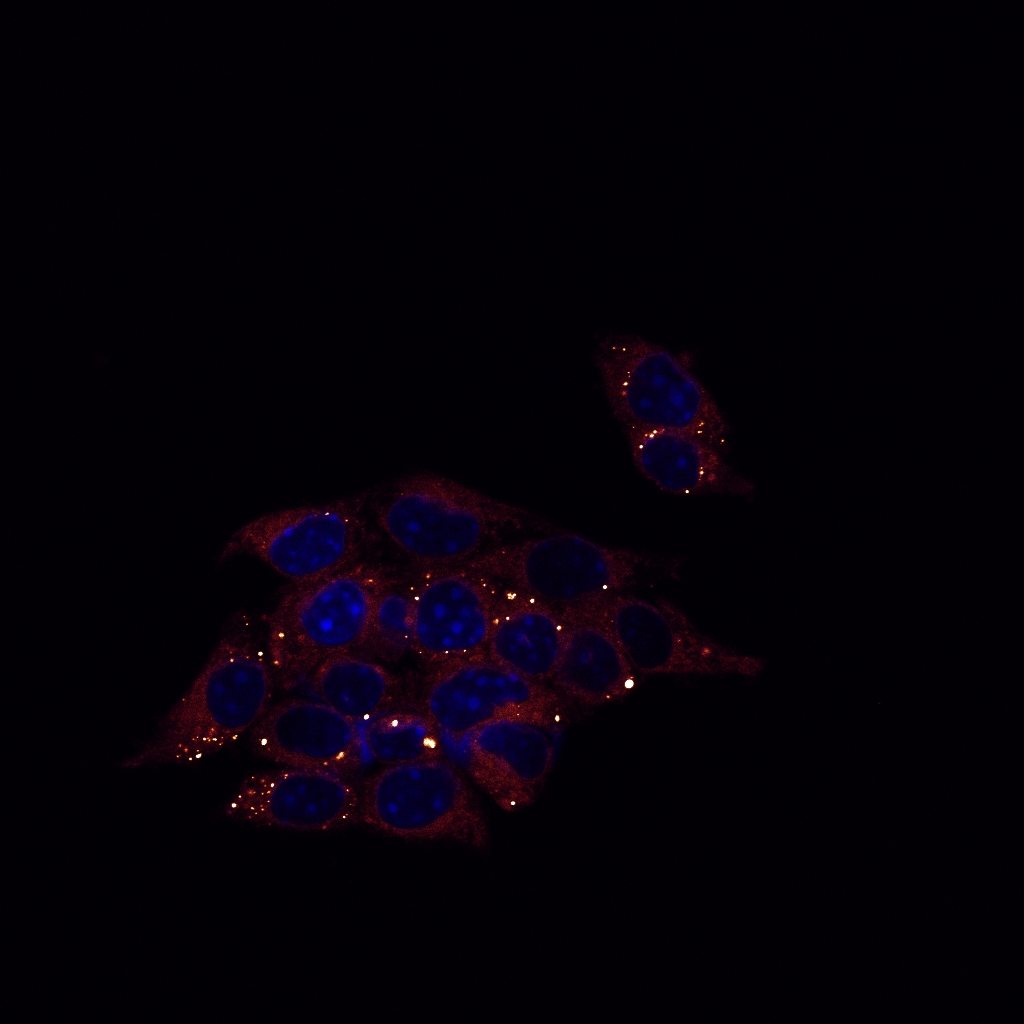

Supplement: Supplementary file 11 — Source data Fig. 7 [file 44321_2024_90_MOESM11_ESM.zip › Figure 7/7B/67NR + RSL3 LDs.jpg]

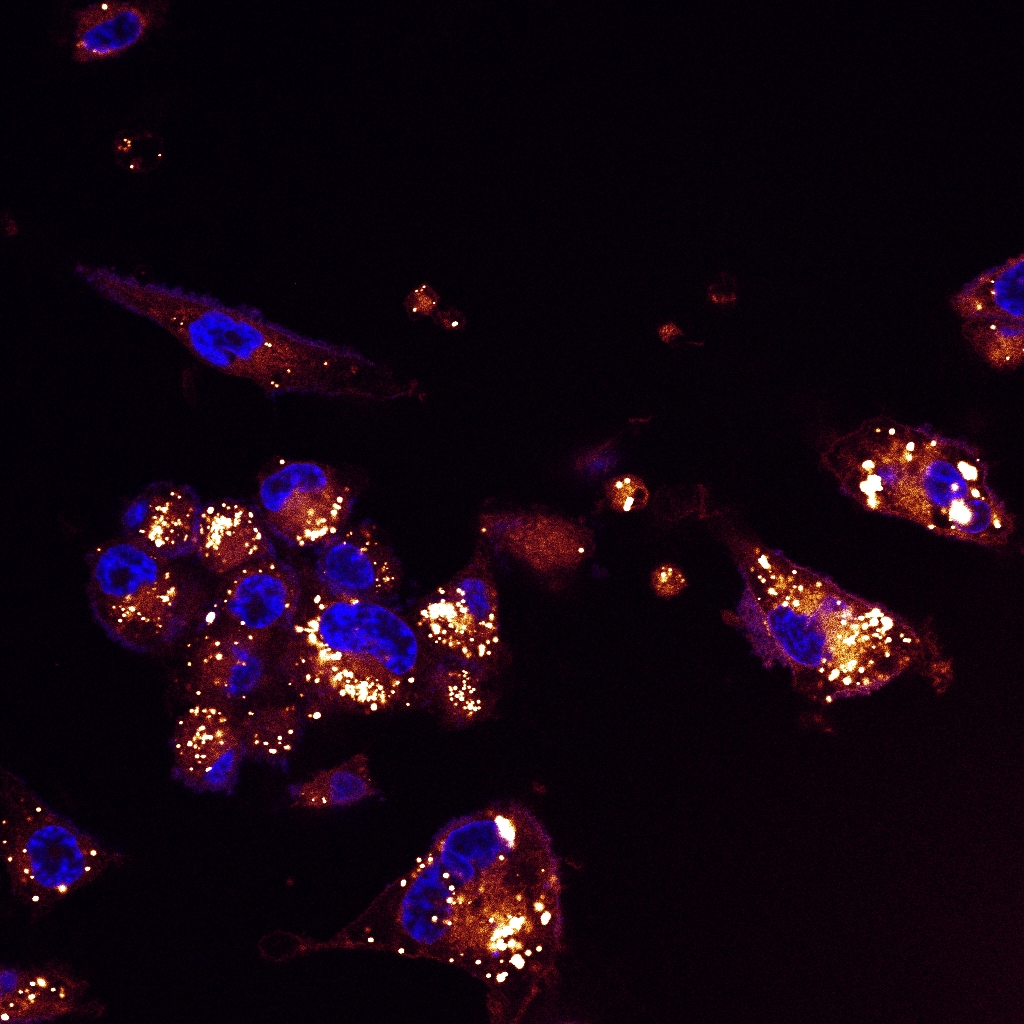

Supplement: Supplementary file 11 — Source data Fig. 7 [file 44321_2024_90_MOESM11_ESM.zip › Figure 7/7B/MDA-MB-231 + RSL3 LDs.jpg]

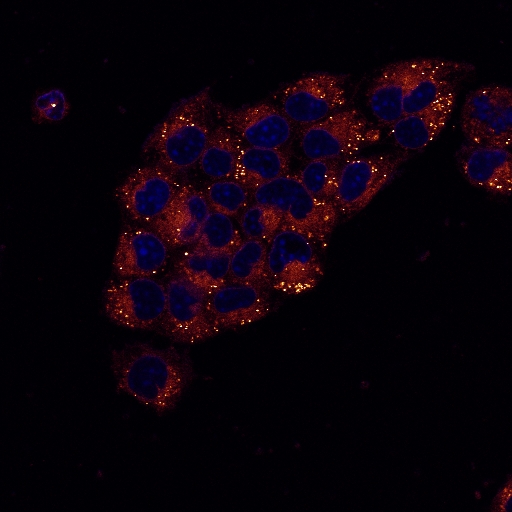

Supplement: Supplementary file 11 — Source data Fig. 7 [file 44321_2024_90_MOESM11_ESM.zip › Figure 7/7A/4T1 + SC-26196 LDs.jpg]

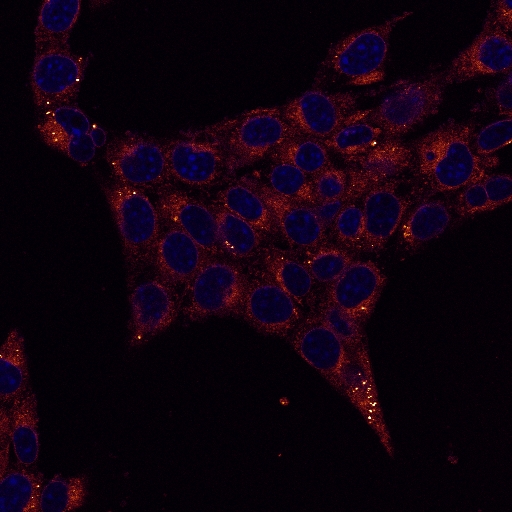

Supplement: Supplementary file 11 — Source data Fig. 7 [file 44321_2024_90_MOESM11_ESM.zip › Figure 7/7A/4T1 + CP-24879 LDs.jpg]

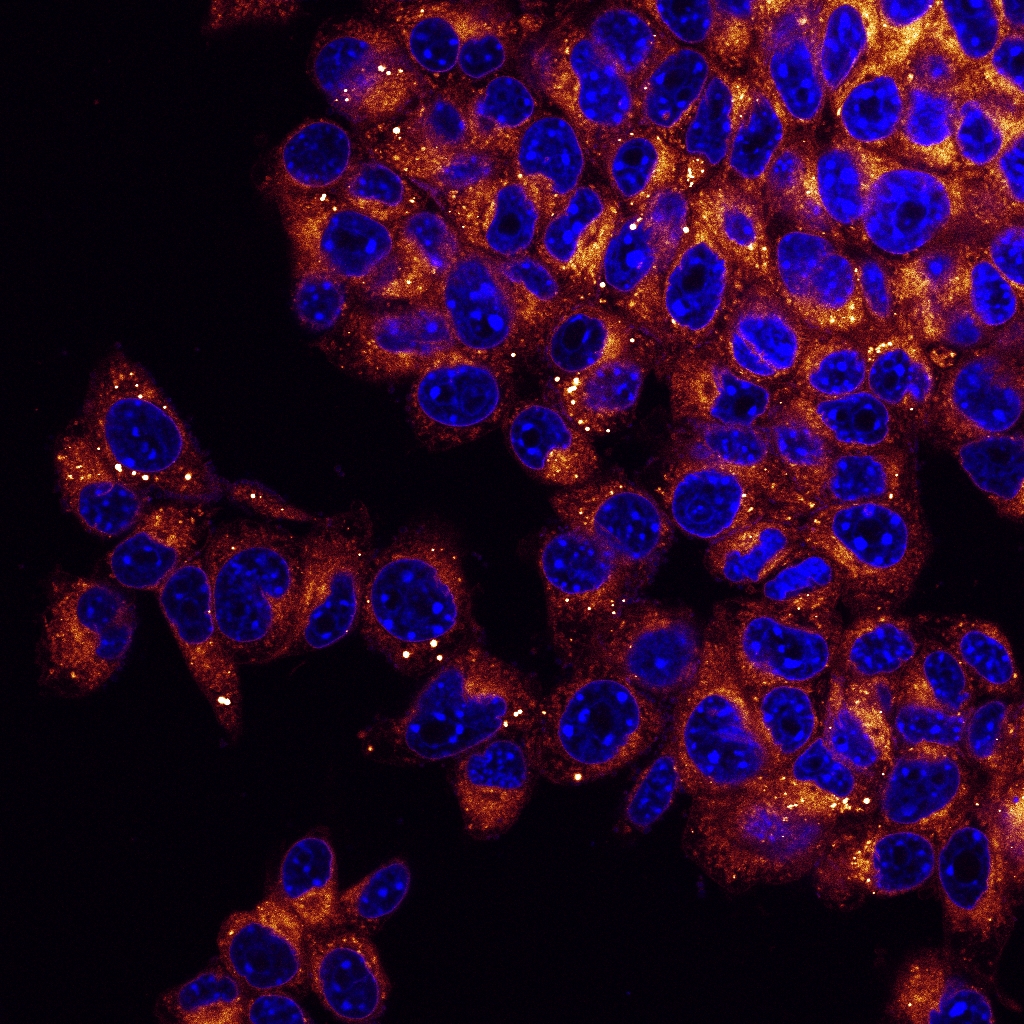

Supplement: Supplementary file 11 — Source data Fig. 7 [file 44321_2024_90_MOESM11_ESM.zip › Figure 7/7A/4T1 NT LDs.jpg]
