# Supplementary material for: FADS1/2 control lipid metabolism and ferroptosis susceptibility in triple-negative breast cancer
Source: EMBO Mol Med. 2024 Jun 26;16(7):5. doi: 10.1038/s44321-024-00090-6 (PMC11251055; doi:10.1038/s44321-024-00090-6)
Supplement: Supplementary file 6 — Source data Fig. 2 [file 44321_2024_90_MOESM6_ESM.zip › Figure 2/2F.pptx]

## Slide 1
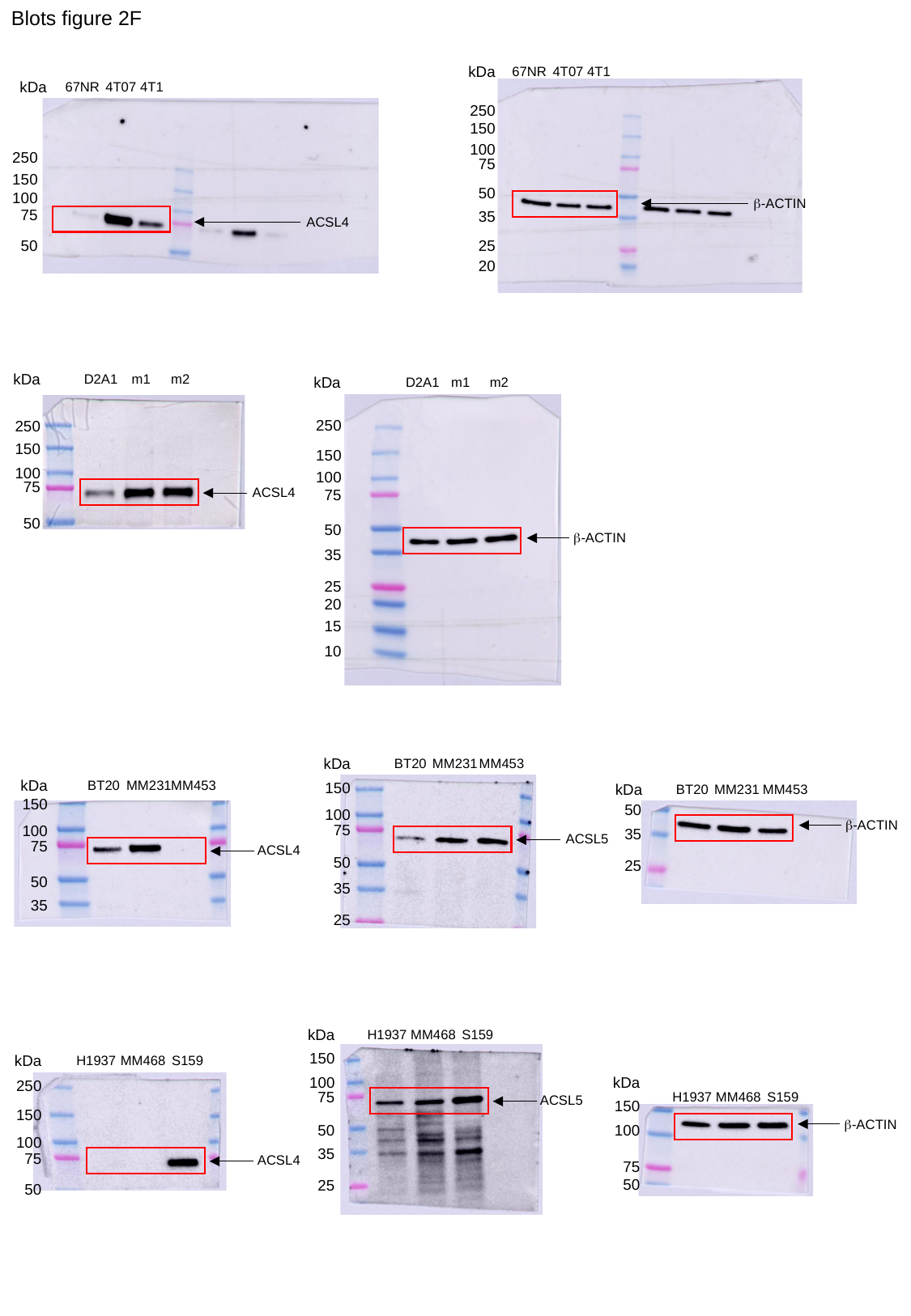

Blots figure 2F
kDa
67NR
4T07
4T1
kDa
67NR
4T07
4T1
250
150
100
250
75
150
50
100
β-ACTIN
75
35
ACSL4
50
25
20
kDa
D2A1
m1
m2
kDa
D2A1
m1
m2
250
250
150
150
100
100
75
ACSL4
75
50
50
β-ACTIN
35
25
20
15
10
kDa
BT20
MM231
MM453
kDa
BT20
MM231
MM453
150
kDa
BT20
MM231
MM453
150
50
100
β-ACTIN
75
100
35
ACSL5
75
ACSL4
50
25
50
35
35
25
kDa
H1937
MM468
S159
150
kDa
H1937
MM468
S159
100
kDa
250
75
H1937
MM468
S159
ACSL5
150
150
β-ACTIN
50
100
100
35
75
ACSL4
75
50
25
50
