# Supplementary material for: FADS1/2 control lipid metabolism and ferroptosis susceptibility in triple-negative breast cancer
Source: EMBO Mol Med. 2024 Jun 26;16(7):5. doi: 10.1038/s44321-024-00090-6 (PMC11251055; doi:10.1038/s44321-024-00090-6)
Supplement: Supplementary file 6 — Source data Fig. 2 [file 44321_2024_90_MOESM6_ESM.zip › Figure 2/2A.pptx]

## Slide 1
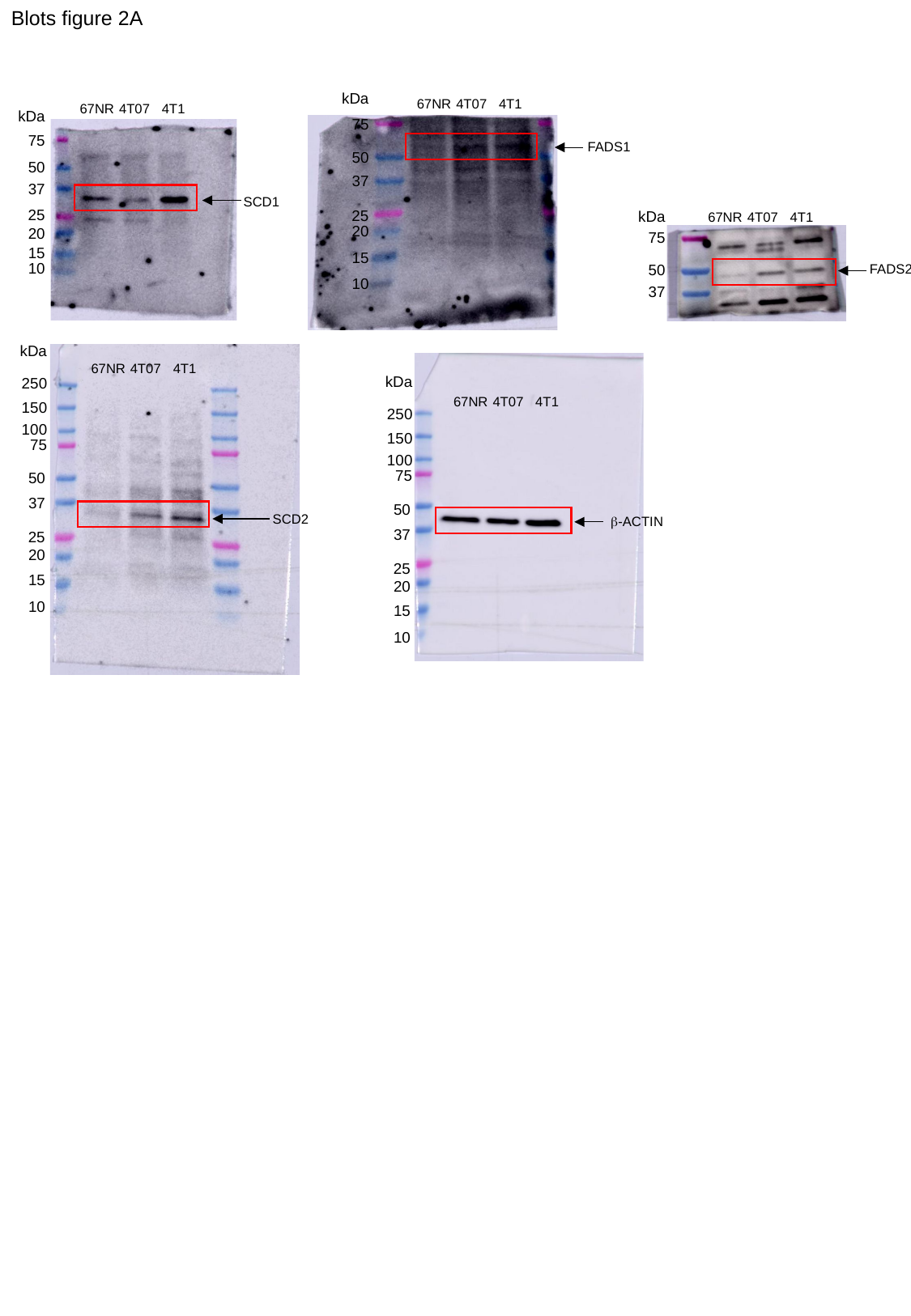

Blots figure 2A
kDa
67NR
4T07
4T1
67NR
4T07
4T1
kDa
75
75
FADS1
50
50
37
37
SCD1
25
25
kDa
67NR
4T07
4T1
20
20
75
15
15
10
50
FADS2
10
37
kDa
67NR
4T07
4T1
kDa
250
67NR
4T07
4T1
150
250
100
150
75
100
75
50
37
50
SCD2
β-ACTIN
37
25
20
25
15
20
10
15
10

## Slide 2
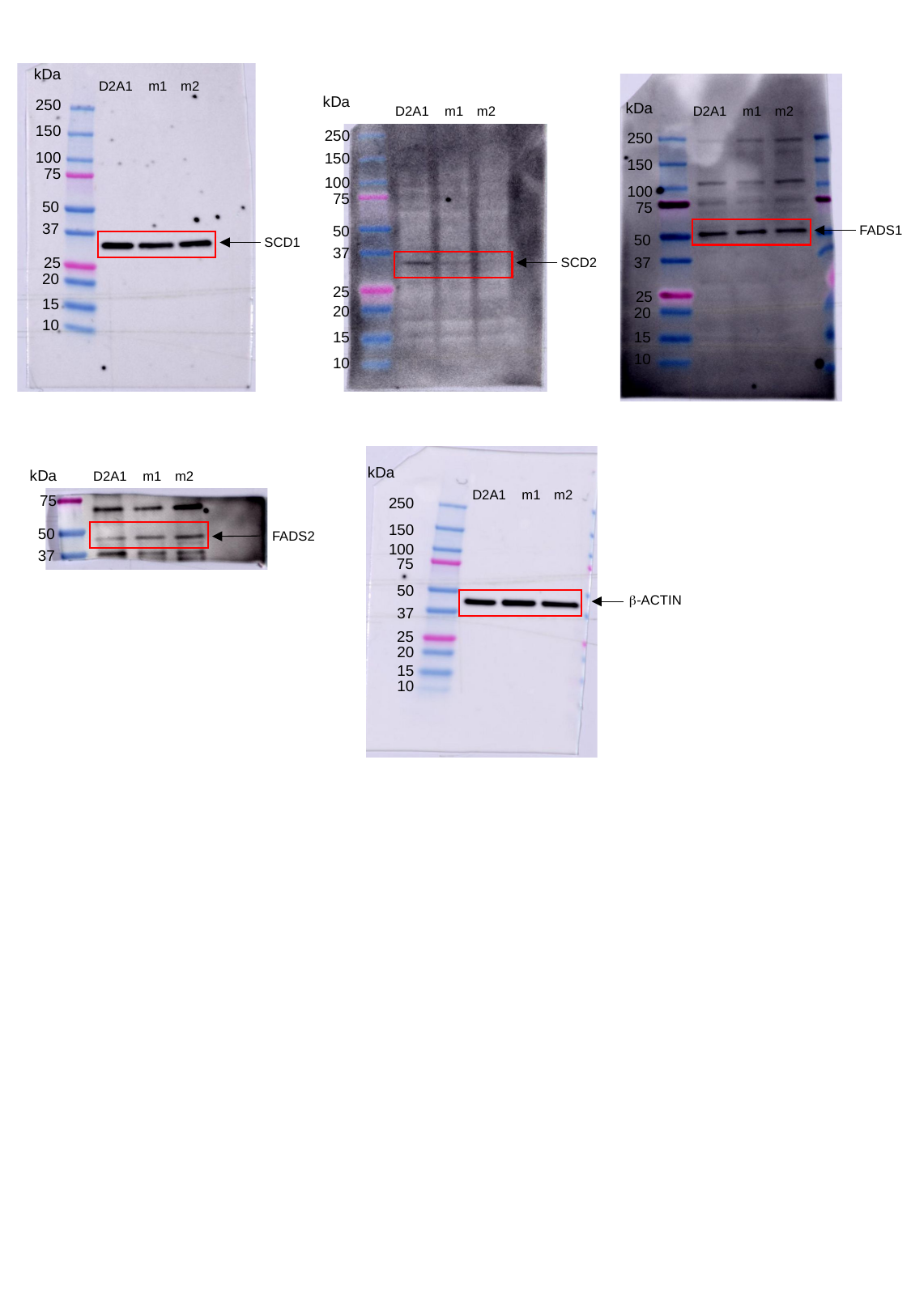

kDa
D2A1
m1
m2
kDa
250
kDa
D2A1
m1
m2
D2A1
m1
m2
150
250
250
100
150
150
75
100
100
75
50
75
37
50
FADS1
50
SCD1
37
37
25
SCD2
20
25
25
15
20
20
10
15
15
10
10
kDa
kDa
D2A1
m1
m2
D2A1
m1
m2
75
250
150
50
FADS2
100
37
75
50
β-ACTIN
37
25
20
15
10

## Slide 3
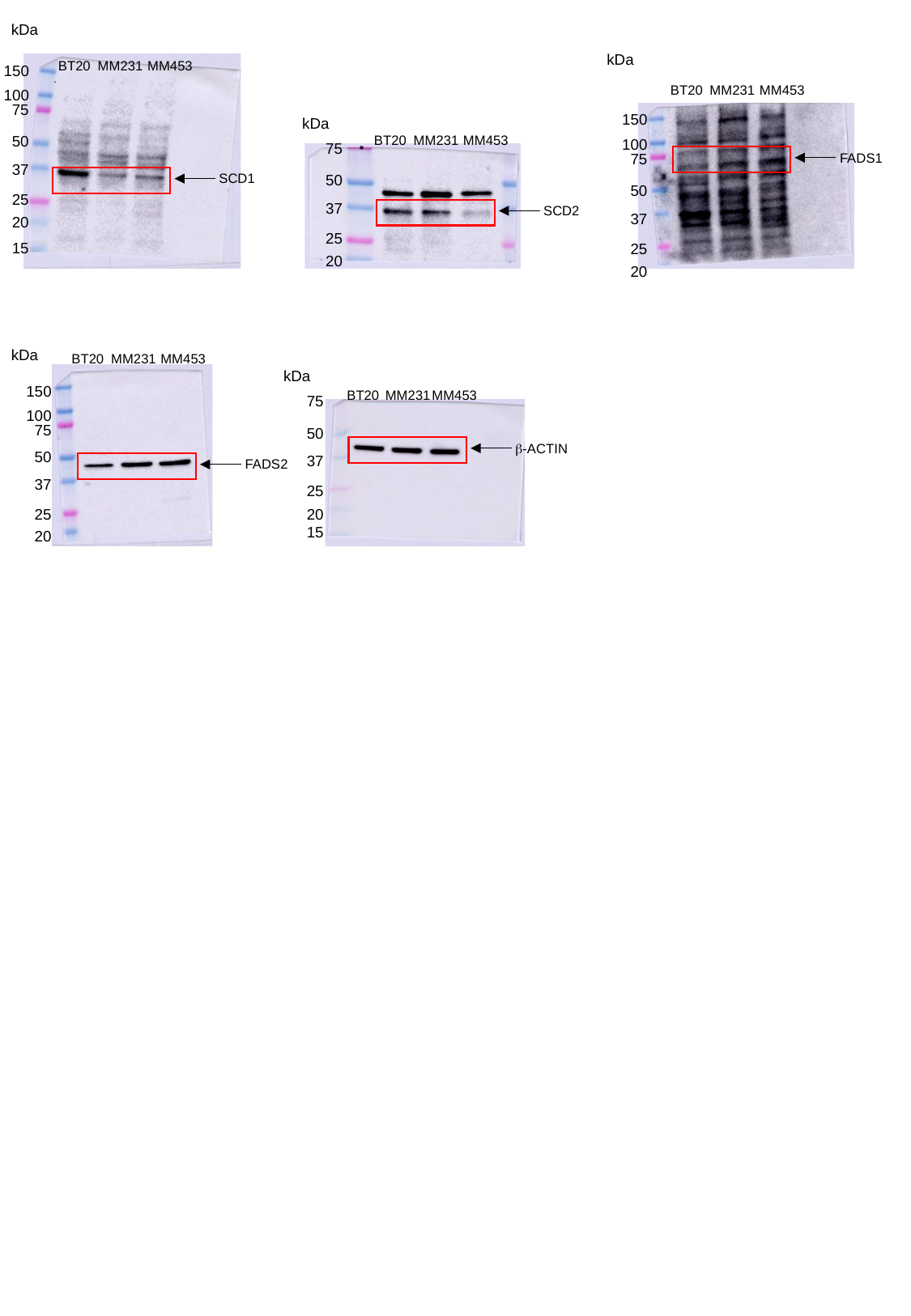

kDa
kDa
BT20
MM231
MM453
150
BT20
MM231
MM453
100
75
150
kDa
50
BT20
MM231
MM453
100
75
FADS1
75
37
SCD1
50
50
25
37
SCD2
37
20
25
15
25
20
20
kDa
BT20
MM231
MM453
kDa
150
BT20
MM231
MM453
75
100
75
50
β-ACTIN
50
37
FADS2
37
25
20
25
15
20

## Slide 4
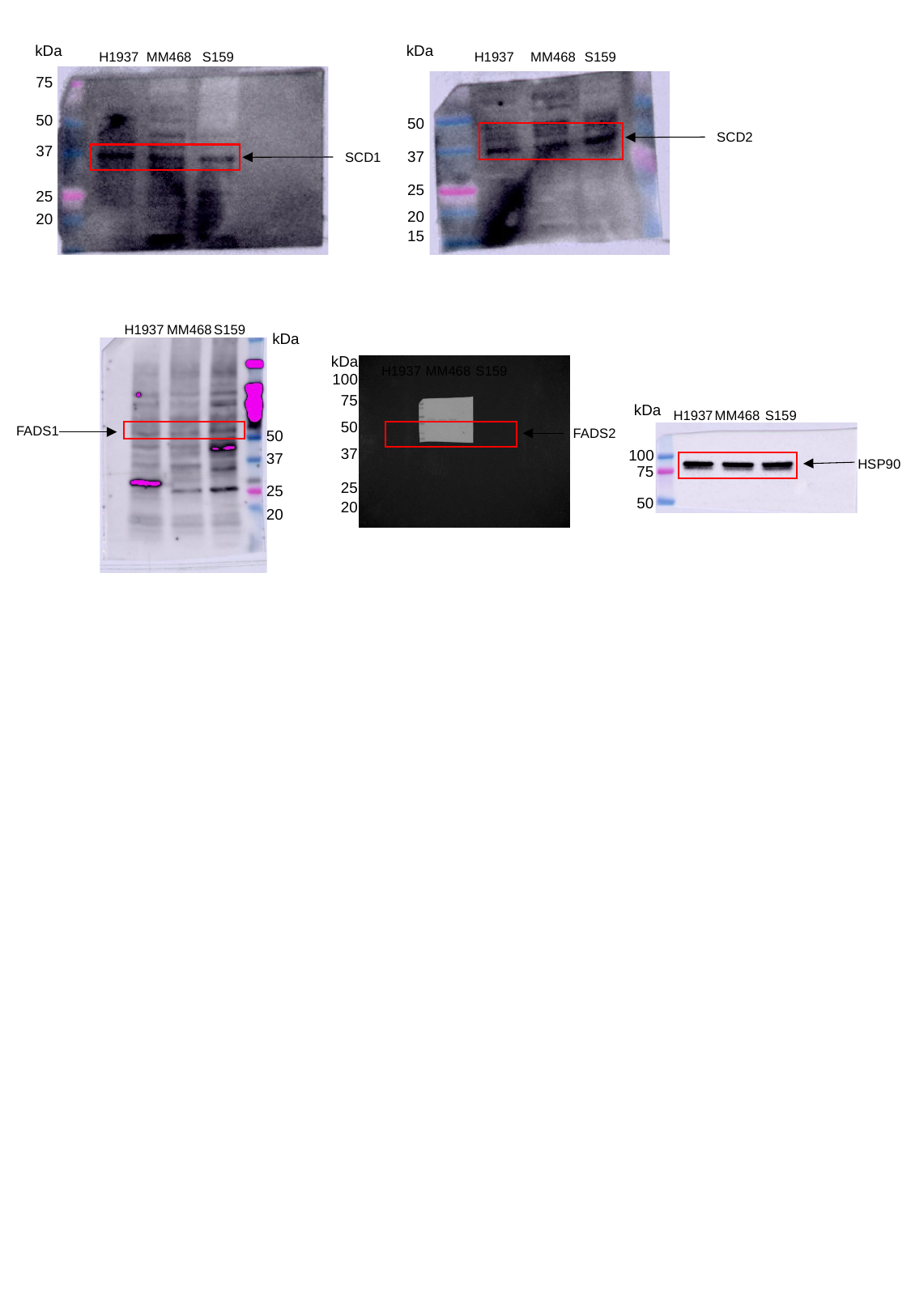

kDa
kDa
H1937
MM468
S159
H1937
MM468
S159
75
50
50
SCD2
37
37
SCD1
25
25
20
20
15
H1937
MM468
S159
kDa
kDa
H1937
MM468
S159
100
75
kDa
H1937
MM468
S159
50
FADS1
FADS2
50
37
100
37
HSP90
75
25
25
50
20
20
