# Supplementary material for: FADS1/2 control lipid metabolism and ferroptosis susceptibility in triple-negative breast cancer
Source: EMBO Mol Med. 2024 Jun 26;16(7):5. doi: 10.1038/s44321-024-00090-6 (PMC11251055; doi:10.1038/s44321-024-00090-6)
Supplement: Supplementary file 13 — Expanded View Figures [file 44321_2024_90_MOESM13_ESM.pdf]

Expanded View Figures

Figure EV1. Details of altered lipid metabolism in aggressive TNBC cells, related to Fig. 1.

(**A**) 67NR, 4T07, and 4T1 breast cancer cells were cultured ON in a medium containing ^{14}C-U-(uniformly) radioactively labeled glucose, lactate, acetate, or glutamine. Lipids were extracted and the radioactive signal was measured to monitor the amount of each metabolite that is incorporated into lipids, as described in the Materials and Methods section. Each value was normalized on protein content ($n = 3$ biological replicates in either single, duplicate, triplicate or more than three technical replicates). The 67NR cell line was used as comparator in the statistical analysis. (**B**, **C**) Murine 4T1 (**B**) and D2A1 (**C**) breast cancer cell series were analyzed by quantitative real-time polymerase chain reaction (qRT-PCR) analysis using the assays described in the figure. Fold relative enrichment is shown using the non-metastatic cells as comparator ($n = 3$ biological replicates in either single or technical duplicate). The 67NR or the D2A1 cells were used as comparator in the statistical analysis. (**D**) The 4T1, D2A1, and human TNBC cells were subjected to cytofluorimetric analysis. FACS analysis of the mean fluorescence intensity (MFI) of the populations positive for BODIPY$^{493/503}$ was reported ($n = 3$ biological replicates in either single or technical triplicate). The non-metastatic cells were used as comparators in the statistical analysis. (**E**) Murine 4T1 and D2A1 series were analyzed by qRT-PCR analysis using the assay described in the figures. Fold relative enrichment is shown using the 67NR or the D2A1 cells as comparator ($n = 3$ biological replicates in either single or technical duplicate). The 67NR or the D2A1 cells were used as comparator in the statistical analysis. (**F**, **G**) Seahorse XFe96 Mito Stress Test was performed on 4T1 cell line series treated with 2 μM UK5099 (mitochondrial pyruvate carrier, MPC, inhibitor), 3 μM BPTES (glutaminase 1, GLS-1, inhibitor), or 4 μM Etomoxir (CPT1A inhibitor) for 30 min in the presence of standard condition (full medium), and oxygen consumption rate (OCR) was calculated in real-time after the administration of the ATP synthase inhibitor oligomycin, the proton uncoupler carbonyl cyanide p-triflouromethoxyphenylhydrazone (FCCP), and the respiratory complex I inhibitor rotenone together with the respiratory complex III inhibitor antimycin A (Rot/AA) (**F**). Basal and maximal respiration was calculated as described in the Materials and Methods section and normalized on protein content (**G**) ($n = 3$ biological replicates in either single, duplicate, triplicate, or more than three technical replicates). The untreated (NT) condition was used as comparator in the statistical analysis. (**H**) Murine TNBC cells were treated with 40 μM of Etomoxir for 30 min. After detachment, cells were subjected to high-resolution respirometry analysis by the Oroboros-O2K instrument. Left: Representative graphs of cell respirometry analysis in the control (up) and treatment (down) conditions. The blue curve represents the oxygen concentration, whereas the red slope shows the oxygen consumption before and after the serial injections of oligomycin (O), uncoupler CCCP (C), and Antimycin A (A). Right: Bar chart graph of basal oxygen consumption (Routine), proton leak (Leak), and maximal oxygen consumption (E) values subtracted from residual oxygen consumption (ROX) in control and Etomoxir treated cells ($n = 3$ biological replicates). Data information: data are presented as mean ± SEM. Statistical analysis was performed using one-way ANOVA followed by Tukey's correction (**A**, **G**) or Dunnett's correction (**B**–**E**), two-way ANOVA followed Bonferroni's correction (**F**) or Tukey's correction (**H**). Source data are available online for this figure.

© The Author(s)

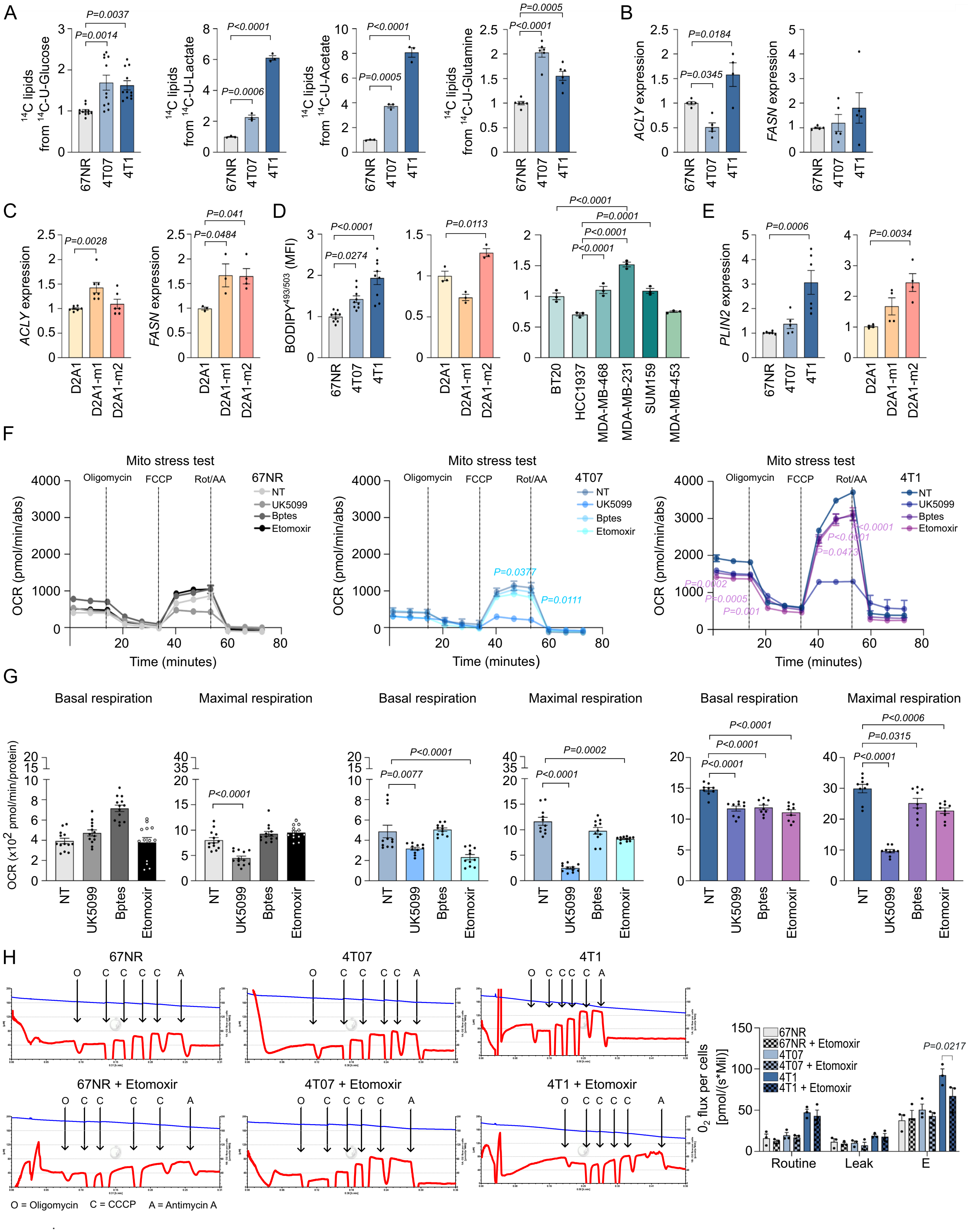
A
^{14}C lipids from ^{14}C-U-Glucose
^{14}C lipids from ^{14}C-U-Lactate
^{14}C lipids from ^{14}C-U-Acetate
^{14}C lipids from ^{14}C-U-Glutamine
67NR 4T07 4T1
P=0.0037
P=0.0014
P<0.0001
P=0.0006
P=0.0005
P=0.0005
P<0.0001
B
ACLY expression
FASN expression
P=0.0184
P=0.0345
C
ACLY expression
FASN expression
D2A1 D2A1-m1 D2A1-m2
P=0.0028
P=0.041
P=0.0484
D
BODIPY$^{493/503}$ (MFI)
P<0.0001
P=0.0274
P=0.0113
BT20 HCC1937 MDA-MB-468 MDA-MB-231 SUM159 MDA-MB-453
E
PLIN2 expression
P=0.0006
P=0.0034
F
Mito stress test
Oligomycin FCCP Rot/AA
67NR
4T07
4T1
NT
UK5099
Bptes
Etomoxir
OCR (pmol/min/abs)
Time (minutes)
P=0.0377
P=0.0111
G
Basal respiration
Maximal respiration
OCR (x10^2 pmol/min/protein)
NT UK5099 Bptes Etomoxir
P<0.0001
P=0.0077
P=0.0002
P=0.0006
P=0.0315
H
67NR
4T07
4T1
67NR + Etomoxir
4T07 + Etomoxir
4T1 + Etomoxir
O = Oligomycin C = CCCP A = Antimycin A
O$_2$ flux per cells [pmol/(s*Mill)]
Routine
Leak
E
P=0.0217

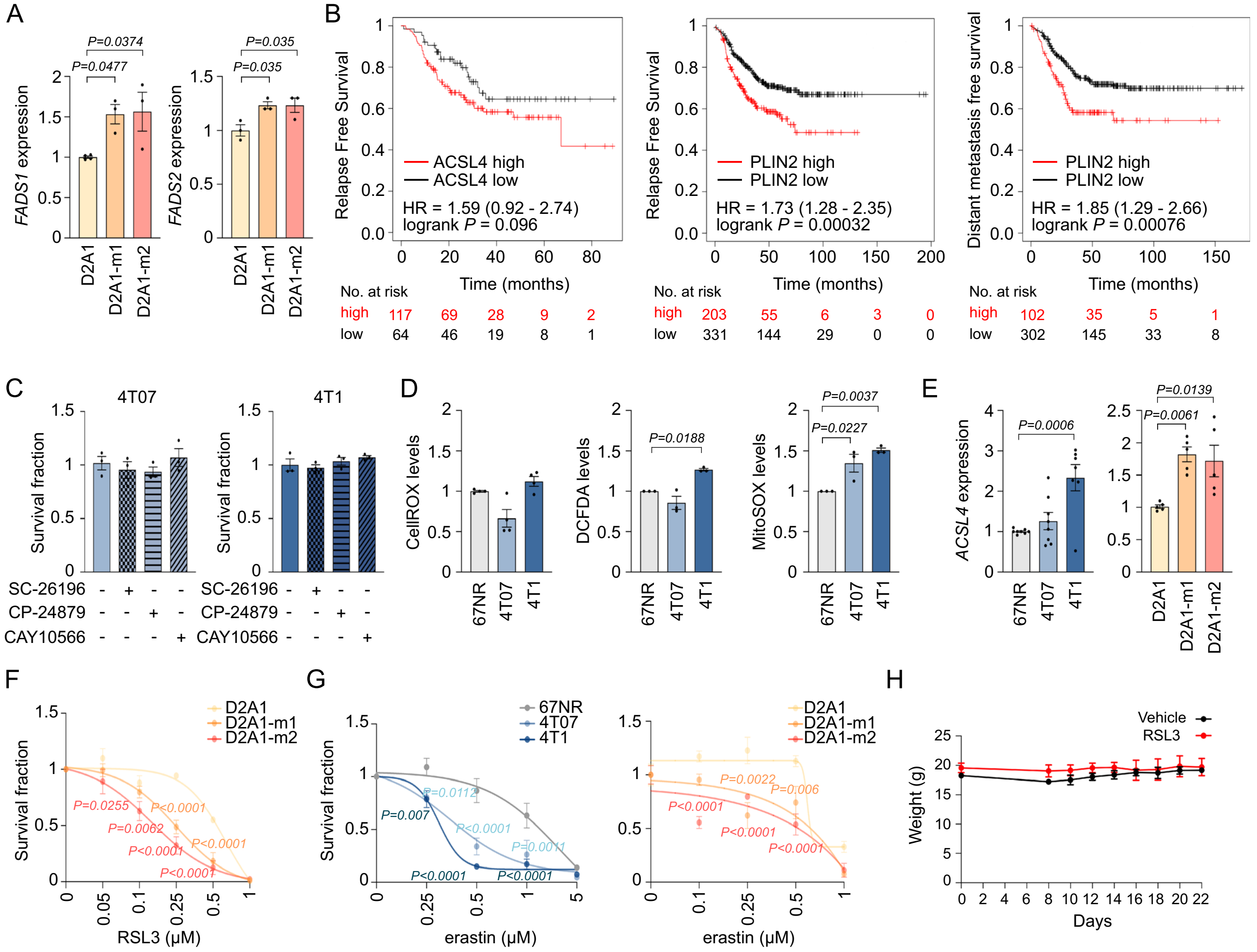

Figure EV2. Details of FADS1 and FADS2 expression in aggressive TNBC and their susceptibility to ferroptosis induction, related to Fig. 2.

(**A**) D2A1 cell line series were analyzed by qRT-PCR analysis using the assays described in the figure. Fold relative enrichment is shown using the D2A1 cells as comparator ($n=3$ biological replicates in either single or technical duplicate). The D2A1 cells were used as comparator in the statistical analysis. (**B**) Kaplan–Meier analysis of RFS and DMFS of a curated cohort of TNBC patients divided into high and low for *ACSL4* or *PLIN2* expression as described in the Materials and Methods section. HR and log-rank Mantel-Cox *P* values are shown. (**C**) TNBC metastatic 4T07 and 4T1 cells were treated with 10 μM FADS2i (SC-26196), FADS1/2i (CP-24879), or SCD1i (CAY10566) for 24 h and subjected to cell viability assay ($n=3$ biological replicates). (**D**) Intracellular ROS levels were measured by CellROX and DCFDA staining while mitochondrial ROS levels by MitoSOX in TNBC 4T1 series' cells ($n=3$ biological replicates in either single or technical duplicate). The 67NR cell line was used as comparator in the statistical analysis. (**E**) Murine TNBC cells were analyzed by qRT-PCR analysis using the assay described in the figure ($n=3$ biological replicates in either single, duplicate, or technical triplicate). Fold relative enrichment and statistical analysis are shown using the non-metastatic (67NR or D2A1) cells as comparator. (**F**, **G**) 24-h dose-response curve of RSL3 (**F**) and erastin (**G**) showed a differential effect between less aggressive and metastatic cells of the D2A1 or 4T1 series ($n=3$ biological replicates). Statistics is shown using the non-metastatic (67NR or D2A1) cells as comparator. (**H**) Weight of BALB/c mice exposed for 15 days to 40 mg/kg RSL3 ($n=4$–6 mice/group). Data information: data are presented as mean ± SEM. Statistical analysis was performed using one-way ANOVA followed by Dunnett's correction (**A**, **C**, **E**) or Tukey's correction (**D**), two-way ANOVA followed by Bonferroni's correction (**F**–**H**). Source data are available online for this figure.

© The Author(s)

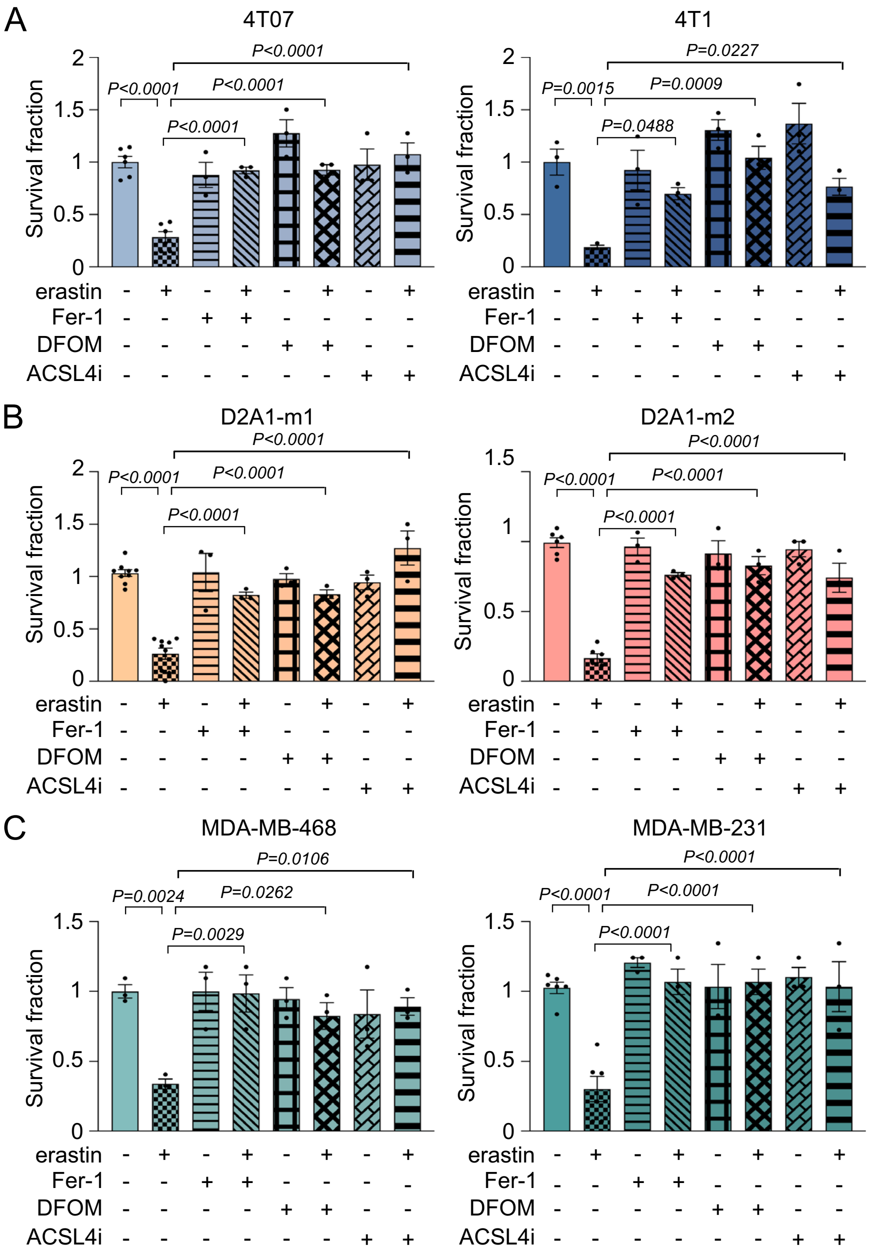

Figure EV3. Details of Ferrostatin-1, Deferoxamine, and ACSL4 inhibition ability to prevent RSL3-induced cell death in TNBC cells, related to Fig. 3.

(**A–C**) Metastatic 4T07 and 4T1 (**A**), D2A1-m1 and D2A1-m2 (**B**), MDA-MB-468 and MDA-MB-231 (**C**) cells were pre-treated with 15 μM Fer-1, 5 μM DFOM, or 10 μM ACSL4i for 4 h and then exposed ON to 0.5 μM erastin. After 24 h cells were subjected to cell viability assay ($n = 3$ biological replicates in either single, duplicate, or technical triplicate). The erastin-treated condition was used as comparator in the statistical analysis. Data information: data are presented as mean ± SEM. Statistical analysis was performed using one-way ANOVA, Dunnett corrected. Source data are available online for this figure.

 © The Author(s)

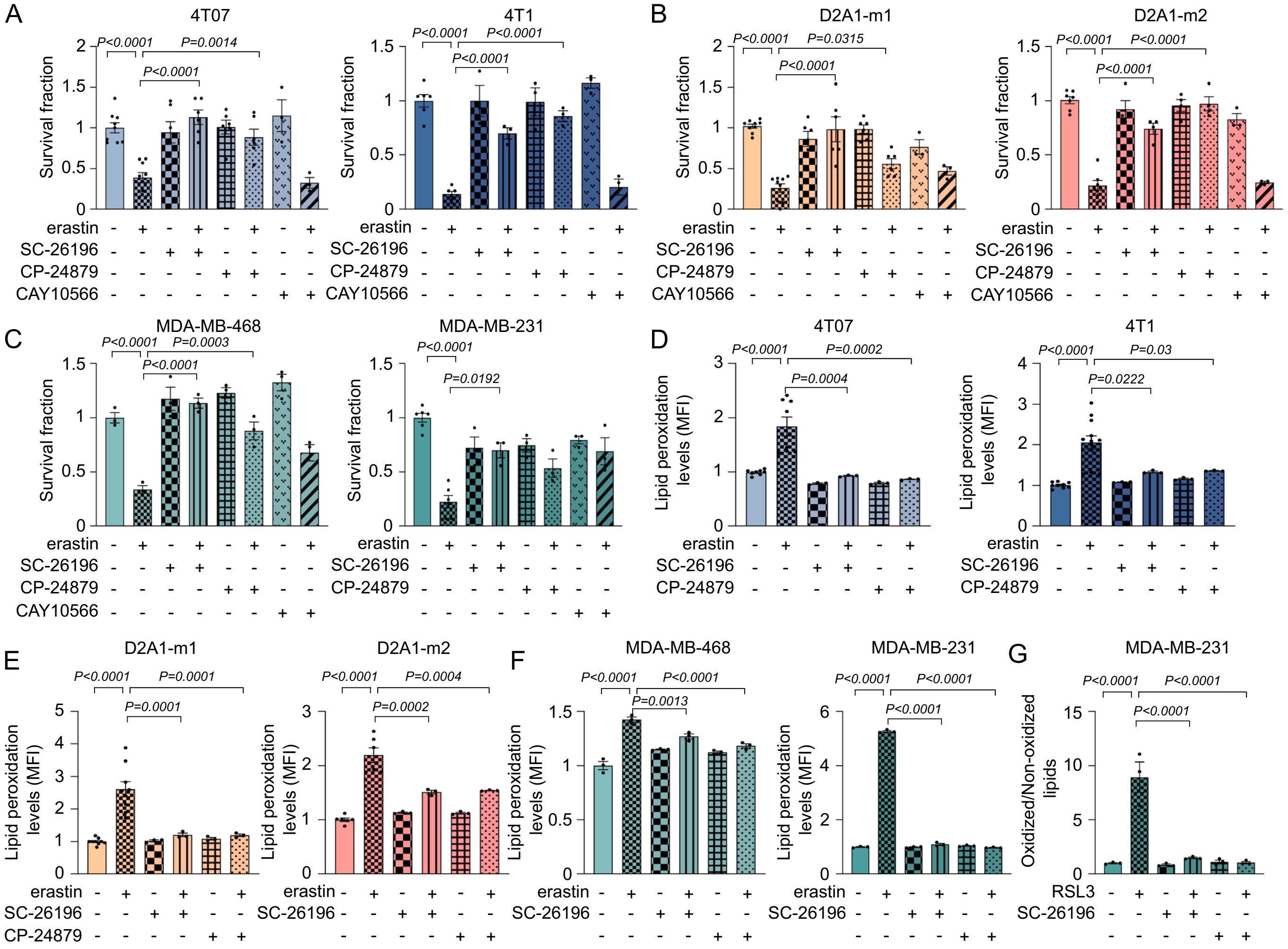

Figure EV4. Details of the ability of FADS1/2 targeting in preventing ferroptosis induction in aggressive TNBC, related to Fig. 4.

(**A–C**) TNBC metastatic 4T07 and 4T1 (**A**), D2A1-m1 and D2A1-m2 (**B**), human MDA-MB-468 and MDA-MB-231 (**C**) cells were pre-treated with 10 μM FADS2i (SC-26196), FADS1/2i (CP-24879), and SCD1i (CAY10566) for 4 h, exposed ON to 0.5 μM erastin, and subjected to cell viability assay ($n = 3$ biological replicates in either single, duplicate or technical triplicate). The erastin-treated condition was used as comparator in the statistical analysis. (**D–F**) TNBC metastatic 4T07 and 4T1 (**D**), D2A1-m1 and D2A1-m2 (**E**), human MDA-MB-468 and MDA-MB-231 (**F**) cells were pre-treated with 10 μM FADS2i (SC-26196), FADS1/2i (CP-24879), and SCD1i (CAY10566) for 4 h, exposed for 2 h to 5 μM erastin, and subjected to cytofluorimetric analysis to measure lipid peroxidation ($n = 3$ biological replicates in either single, duplicate or technical triplicate). The erastin-treated condition was used as comparator in the statistical analysis. (**G**) Highly metastatic MDA-MB-231 cells were pre-treated with 10 μM FADS2i or FADS1/2i for 4 h, exposed for 2 h to 1 μM RSL3, and subjected to confocal analysis to measure lipid peroxidation. Relative quantification is shown ($n = 3$ biological replicates). The RSL3-treated condition was used as comparator in the statistical analysis. Data information: data are presented as mean ± SEM. Statistical analysis was performed using one-way ANOVA followed by Dunnett's correction (**A–F**) or Tukey's correction (**G**). Source data are available online for this figure.

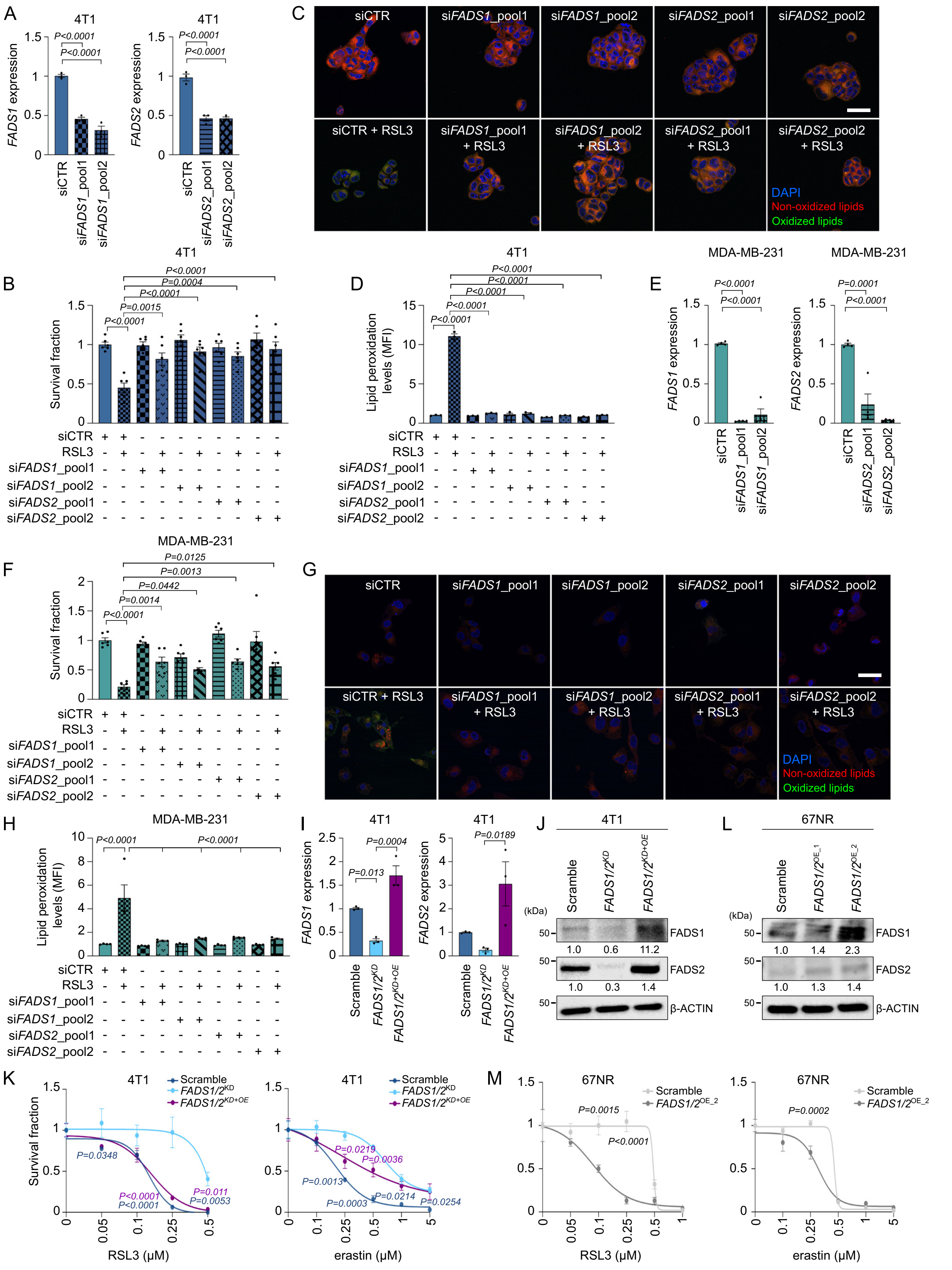

A
4T1
FADS1 expression
FADS2 expression
P<0.0001
siCTR
siFADS1_pool1
siFADS1_pool2
siFADS2_pool1
siFADS2_pool2
C
siCTR + RSL3
siFADS1_pool1 + RSL3
siFADS1_pool2 + RSL3
siFADS2_pool1 + RSL3
siFADS2_pool2 + RSL3
DAPI
Non-oxidized lipids
Oxidized lipids
B
Survival fraction
P=0.0004
P=0.0015
RSL3
D
Lipid peroxidation levels (MFI)
E
MDA-MB-231
P=0.0001
F
P=0.0125
P=0.0013
P=0.0442
P=0.0014
G
H
I
Scramble
FADS1/2KD
FADS1/2KD+OE
P=0.0004
P=0.013
P=0.0189
J
(kDa)
FADS1
FADS2
β-ACTIN
L
67NR
FADS1/2OE_1
FADS1/2OE_2
K
P=0.0348
P<0.0001
P=0.011
P=0.0053
RSL3 (μM)
P=0.0219
P=0.0036
P=0.0013
P=0.0003
P=0.0214
P=0.0254
erastin (μM)
M
P=0.0015
P=0.0002

 © The Author(s)

Figure EV5. Details of FADS1/2 knockdown effect on lipidomic profile of TNBC cells and their susceptibility to ferroptosis induction, related to Fig. 5.

(**A–D**) 4T1 cells transfected with non-targeting small interfering RNA (siCTR) or 2 different combination pools of 4 individual siRNA for FADS1 (siFADS1_pool1 and pool2) and FADS2 (siFADS2_pool1 and pool2) and assessed by qRT-PCR (**A**) were treated ON with 0.1 μM RSL3 (**B**) or 2 h with 1 μM RSL3 (**C**, **D**) and assayed for cell survival (**B**), confocal analysis (**C**), and cytofluorimetric analysis (**D**) to measure lipid peroxidation. Representative pictures of $BODIPY^{581/591}$-C11 stained cells are shown (oxidized lipids: green; non-oxidized lipids: red; nuclei: blue, DAPI; scale bar, 10 μm) ($n = 3$ biological replicates in either single or technical duplicate). siCTR transfected cells (**A**) and siCTR transfected cells treated with RSL3 (**B**, **D**) were used as comparator in the statistical analysis. (**E–H**) MDA-MB-231 cells transfected with siCTR or 2 different combination pools of 4 individual siRNA for FADS1 (siFADS1_pool1 and pool2) and FADS2 (siFADS2_pool1 and pool2) and assessed by qRT-PCR (**E**) were treated ON with 0.25 μM RSL3 (**F**) or 2 h with 1 μM RSL3 (**G**, **H**) and assayed for cell survival (**F**), confocal analysis (**G**), and cytofluorimetric analysis (**H**) to measure lipid peroxidation. Representative pictures of $BODIPY^{581/591}$-C11 stained cells are shown (oxidized lipids: green; non-oxidized lipids: red; nuclei: blue, DAPI; scale bar, 10 μm) ($n = 3$ biological replicates in either single or technical duplicate). siCTR transfected cells (**E**) and siCTR transfected cells treated with RSL3 (**F**, **H**) were used as comparator in the statistical analysis. (**I–K**) Stable *FADS1/FADS2* double knockdown transfected 4T1 cells ($FADS1/2^{KD}$ 4T1) and the corresponding cells with the ectopic re-introduction of FADS1 and FADS2 ($FADS1/2^{KD+OE}$ 4T1), whose FADS1/2 expression was assessed using qRT-PCR and WB analyses (**I**, **J**), were exposed to increasing concentrations of RSL3 or erastin in a dose-response curve assay (**K**) ($n = 3$ biological replicates). The $FADS1/2^{KD}$ condition was used as comparator in the statistical analysis. (**L**, **M**) 67NR cells over-expressing both FADS1 and FADS2, $FADS1/2^{OE}$, (**L**) were grown for 24 h in the presence of increasing doses of RSL3 and erastin (**M**) before assaying cell viability ($n = 3$ biological replicates). Data information: In (**A**, **B**, **D–F**, **H**, **I**, **K**, **M**) data are presented as mean ± SEM. Statistical analysis was performed using one-way ANOVA followed by Dunnett's correction (**A**, **B**, **D–F**, **H**, **I**) or two-way ANOVA followed by Bonferroni's correction (**K**, **M**). Source data are available online for this figure.

© The Author(s)
